# Global trends, inequalities, and pathogen shifts in infectious diarrhea among children under five: a comprehensive analysis of the global burden of disease study 1990–2021

**DOI:** 10.3389/fnut.2025.1679081

**Published:** 2025-11-14

**Authors:** Qiangqiang Tian, Ya Zheng, Yishudong Li, Rui Wu, Yuyu Lin, Zhaofeng Chen

**Affiliations:** 1 Lanzhou University, Lanzhou, Gansu, China; 2 The First Hospital of Lanzhou University, Lanzhou, Gansu, China

**Keywords:** infectious diarrhea, children under five, global burden of disease, health inequality, epidemiological trends

## Abstract

**Background:**

Infectious diarrhea is a major cause of morbidity and mortality among children under five, particularly in low- and middle-income countries. Despite notable improvements in public health, substantial regional, national, and socioeconomic disparities persist, while the evolving pathogen spectrum presents new challenges for prevention and control.

**Methods:**

We conducted a comprehensive analysis using data from the Global Burden of Disease (GBD) 2021 cycles, covering 204 countries and territories from 1990 to 2021. We assessed incidence, prevalence, mortality, and disability-adjusted life years (DALYs) at global, regional, national, and SDI quintile levels. Analytical methods included calculation of age-standardized rates (ASR), estimation of annual percentage changes (EAPC), joinpoint regression, inequality indices (SII and concentration index), frontier analysis, and pathogen-specific DALY trends.

**Results:**

Globally, incident cases and DALYs increased while ASRs for all burden indicators declined substantially from 1990 to 2021. The heaviest burdens persisted in South Asia and Western Sub-Saharan Africa. Although absolute global inequalities lessened, relative disparities in mortality and DALYs widened. Temporal and geographic heterogeneity was evident, with high-income countries increasingly affected by viral etiologies. Frontier analysis revealed notable inefficiency gaps for several countries. Major pathogen-related DALY reductions were observed for rotavirus and Shigella, yet viral causes gained relative prominence in high-SDI settings.

**Conclusion:**

While marked global progress has been achieved in reducing childhood infectious diarrhea burden, persistent, and sometimes widening inequities remain. Enhanced investment in equitable health systems, renewed pathogen surveillance, and adaptive, targeted interventions are needed to sustain and accelerate progress, particularly in the most affected regions.

## Introduction

Infectious diarrhea remains one of the leading causes of morbidity and mortality among children under 5 years of age, particularly in low- and middle-income countries (1–3). Despite substantial advances in public health, sanitation, and the development of preventive interventions—such as oral rehydration therapy and vaccines—diarrheal diseases continue to impose a considerable health burden on young children worldwide (4–8). The complex etiology of infectious diarrhea, including viral, bacterial, and parasitic pathogens, coupled with socioeconomic disparities, contributes to a persistently high disease burden in vulnerable populations (9–11).

While global initiatives have targeted reduction in childhood diarrhea-related mortality, the epidemiological landscape of infectious diarrhea has evolved considerably over recent decades (12–15). There is growing recognition of the substantial heterogeneity in incidence, mortality, and disability-adjusted life years (DALYs) across regions and countries, reflecting differences in healthcare infrastructure, access to safe water and sanitation, socioeconomic status, and the success of specific interventions (16–18). Moreover, the distribution of causative pathogens and the relative importance of different etiological agents appear to be shifting, influencing the impact of preventive strategies (19, 20). Existing research has primarily addressed either single-country trends or selected regions, often failing to capture global, regional, and national disparities, temporal changes, and the impact of demographic and socioeconomic development. Comprehensive, comparative assessments across time and geographies are therefore needed to inform targeted policy and guide ongoing efforts (21–23).

This study aims to provide a systematic and up-to-date assessment of the global burden and temporal trends of infectious diarrhea in children under 5 years old from 1990 to 2021, utilizing data from the Global Burden of Disease (GBD) Study. We analyzed incidence, prevalence, mortality, and DALY metrics at global, regional, and national levels, disaggregated by sex and Socio-demographic Index (SDI). Our approach included estimation of annual percentage changes, joinpoint regression to identify critical shifts in disease trends, and inequality analyses to explore the relationship between socioeconomic development and disease burden. Additionally, we performed frontier analysis to benchmark country-level performance relative to development, and examined the evolving distribution of pathogen-specific DALY rates. By providing a comprehensive overview of spatiotemporal dynamics and determinants, our findings aim to inform strategic actions for further reducing the global burden of childhood infectious diarrhea.

## Methods

### Data source and study population

This study leveraged data from the GBD 2021 cycles, an initiative coordinated by the Institute for Health Metrics and Evaluation (IHME), which provides systematically collected, validated, and standardized global health estimates. We extracted yearly estimates from 1990 to 2021 on infectious diarrhea among children under 5 years of age, encompassing 204 countries and territories disaggregated by sex, age, region, and SDI. Infectious diarrhea was defined according to the GBD protocol (ICD codes and case definitions available in Supplementary Table 1). The population of interest included all children aged under 5 years, with no exclusion on sex or region.

### Outcome measures

The primary outcome metrics included the annual number and age-standardized rates (ASR) per 100,000 population for (1) incidence, (2) prevalence, (3) deaths, and (4) disability-adjusted life years (DALYs) attributable to infectious diarrhea. ASRs were computed using the direct method, applying the GBD world standard population to facilitate temporal and cross-geographical comparisons. Cause-specific DALY rates were estimated as the sum of years of life lost (YLLs) and years lived with disability (YLDs) for each country-year (24, 25).

### Socio-demographic index

The SDI is a composite, annually updated indicator incorporating measures of lag-distributed income per capita, educational attainment, and fertility rates among women under 25 years. SDI ranges from 0 (lowest development) to 1 (highest development) and was used to group countries into quintiles (low, low-middle, middle, high-middle, high) in all analyses. SDI values and quintile thresholds for each year were obtained from GBD metadata (26).

### Descriptive and comparative analyses

We first described temporal trends and geographical variation in total and sex-disaggregated burden of infectious diarrhea by global, regional (GBD super-regions and 21 regions), national, and SDI quintile strata. Annual and interval-specific counts and rates for incidence, prevalence, mortality, and DALYs were summarized. Supplementary Table 1 details the GBD regional groupings and regional boundaries.

### Trend analysis: EAPC estimation

To quantify temporal trends, we estimated the Estimated Annual Percentage Change (EAPC) and corresponding 95% confidence intervals (CIs) for ASRs using regression models of the form: log(*ASR*_*t*_) = α+β*t*+ϵ_*t*_, where *t* is calendar year. The EAPC was calculated as 100 × (exp(β)−1), with statistical significance defined by exclusion of zero from the 95% CI. This approach was conducted globally, regionally, nationally, and by SDI quintile for all primary burden metrics (27, 28).

### Joinpoint regression

We applied Joinpoint regression analysis (Joinpoint Regression Program, Version 4.9; National Cancer Institute, USA) to identify significant inflection points and periods with distinct trends in ASR trajectories from 1990 to 2021. The model allowed for up to four joinpoints per time series, with permutation tests used to select the optimal model. Average annual percent changes (AAPCs) and interval-specific slopes were reported for each identified segment. Analyses were conducted for the global population and within each SDI quintile (29).

### Inequality analysis

To assess socioeconomic inequalities in disease burden, we examined both absolute and relative disparities. The Slope Index of Inequality (SII) quantified the absolute difference in ASR between the lowest and highest ends of the SDI spectrum, estimated using weighted least-squares regression of ASR on ranked SDI. The Concentration Index (CI), reflecting relative inequality, was calculated as twice the area between the concentration curve and the line of equality, with CI values above zero denoting concentration of disease in higher SDI groups and below zero indicating a burden among lower SDI strata. Analyses were repeated for each burden metric at the global level and within GBD regions. Nonlinear associations between SDI and burden metrics were visualized with locally weighted scatterplot smoothing (LOESS) (30).

### Frontier analysis

Frontier efficiency analysis was performed to benchmark countries' performance relative to optimal outcomes for their SDI level. For each burden metric and SDI value, the data envelope frontier was constructed using stochastic frontier analysis, and each country's distance from the efficient frontier line (i.e., “inefficiency gap”) was quantified. Countries substantially above the frontier were classified as underperformers, while those near or below the frontier represented optimal or exceptionally efficient outcomes for prevailing socio-demographic conditions. Analyses focused on data from 1990 to 2021 and highlighted results for 2021 (31, 32).

### Pathogen-specific analysis

Estimates of DALY rates (per 100,000 children under five) and EAPCs by pathogen were extracted for 22 GBD regions and globally from the GBD cause hierarchy, reflecting contributions by key etiological agents (including rotavirus, Shigella, norovirus, Campylobacter, EPEC, adenovirus, and sapovirus). Temporal trends for pathogen-attributable burden and the effectiveness of region-specific interventions were examined using the same EAPC and joinpoint frameworks as for all-cause burden.

## Results

### Global, regional, and national burden

**Figure 1:**
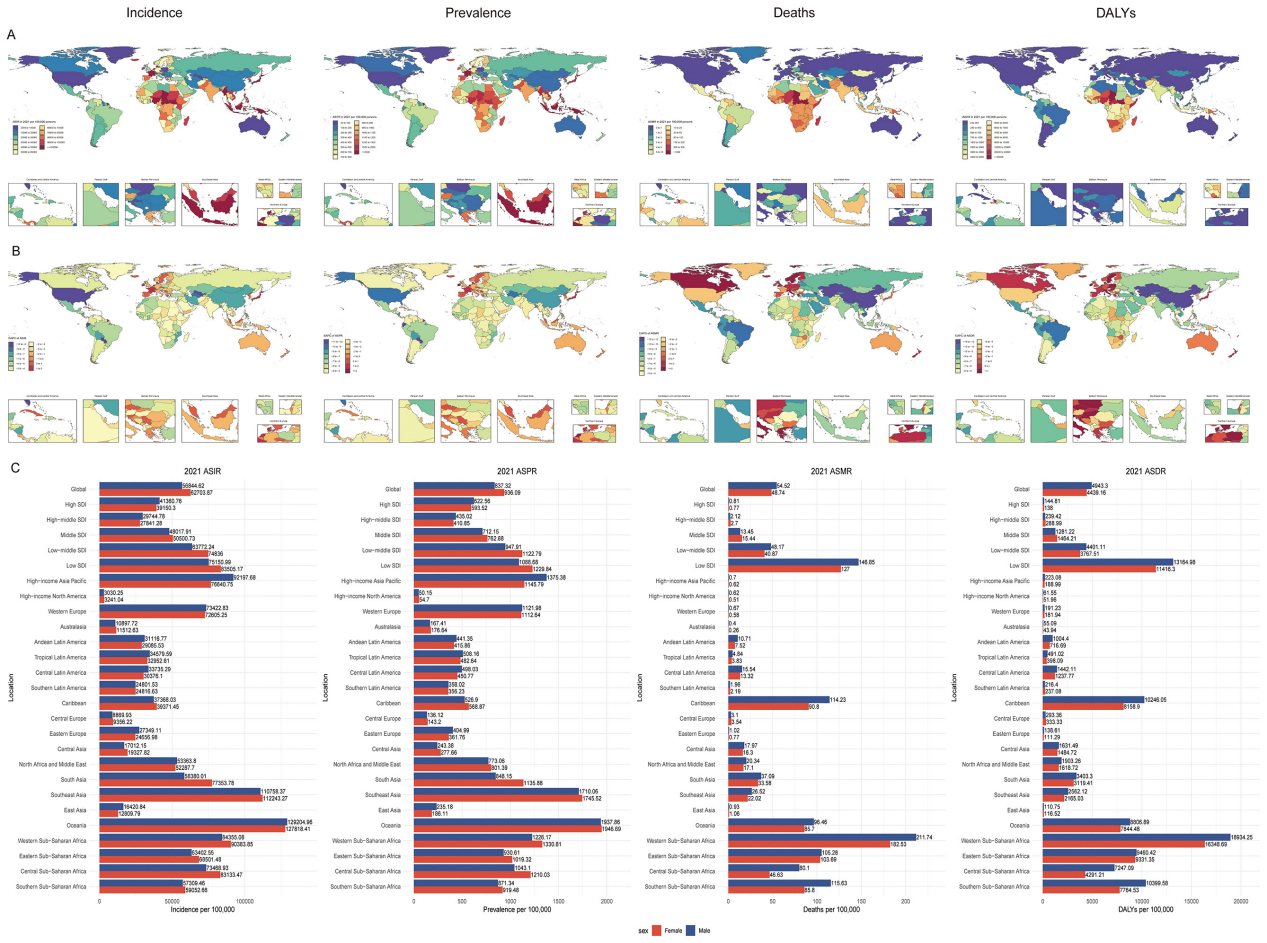
Trends and sex differences in infectious diarrhea among children under five across regions, 1990–2021. **(A)** Age-standardized rate of incidence (ASIR), prevalence (ASPR), mortality (ASMR), disability-adjusted life-year (ASDR) in 204 countries and territories in 2021; **(B)** Estimated annual percentage change (EAPC) of ASIR, ASPR, ASMR, and ASDR in 204 countries and territories from 1990 to 2021; **(C)** ASIR, ASPR, ASMR, ASDR by sex in global and regional populations in 2021. ASR, age-standardized rate; ASIR, Age-standardized incidence rate; ASPR, Age-standardized prevalence rate; ASMR, Age-standardized mortality rate; ASDR, Age-standardized disability-adjusted life-year rate; EAPC, estimated annual percentage change; DALYs, disability-adjusted life-years; SDI, Sociodemographic Index.

From 1990 to 2021, the global number of incident cases and DALYs of infectious diarrhea among children under 5 years increased, while prevalence and mortality counts demonstrated notable declines. Throughout this period, age-standardized rates (ASRs) for incidence, prevalence, mortality, and DALYs all declined significantly, with no marked sex differences observed (Figure 1; Supplementary Tables 1, 2). In 2021, South Asia contributed the highest numbers of incident and prevalent cases. Oceania had the highest ASRs for incidence and prevalence. Western Sub-Saharan Africa bore the highest numbers and rates of deaths and DALYs in this age group (Tables 1, 2).

**Table 1:** The case number and ASR of Incidence and prevalence of infectious diarrhea in children under 5 years of age in 1990 and 2021 for both sexes by SDI quintiles and by GBD regions, with EAPC from 1990 to 2021.

**Location**	**Incidence**					**Prevalence**				
	**Number (95 % UIs).1990**	**Number (95 % UIs).2021**	**ASR (95 % UIs).1990**	**ASR (95 % UIs).2021**	**EAPC (95 % CI) 1990–2021**	**Number (95 % UIs).1990**	**Number (95 % UIs).2021**	**ASR (95 % UIs).1990**	**ASR (95 % UIs).2021**	**EAPC (95 % CI) 1990–2021**
Global	1,178,110,288 (1,000,354,993 to 1,366,541,108)	392,778,890 (324,124,687 to 463,633,235)	190,036.82 (161,363.74 to 220,431.93)	59,677.27 (49,246.22 to 70,442.6)	−3.34 (−3.82 to −2.86)	19,458,645 (1,704,3262 to 22,054,325)	5,825,270 (4,975,326 to 6,775,131)	3,138.81 (2,749.19 to 3,557.51)	885.07 (755.93 to 1,029.39)	−3.7 (−4.18 to −3.22)
High SDI	37,611,903 (27,244,562 to 50,267,709)	21,691,430 (15,513,335 to 28,673,257)	60,947.41 (44,147.87 to 81,455.25)	40,284.05 (28,810.45 to 53,250.29)	−0.67 (−1.14 to −0.21)	579,741 (437,163 to 771,923)	327,606 (245,544 to 430,230)	939.43 (708.39 to 1,250.85)	608.41 (456.01 to 799)	−0.75 (−1.21 to −0.29)
High-middle SDI	96,107,437 (77,466,420 to 11,791,0531)	20,200,475 (15,302,775 to 25,985,679)	103,449.61 (83,384.5 to 126,918.36)	28,839.35 (21,847.11 to 37,098.64)	−4 (−4.38 to −3.61)	1,541,524 (1,269,328 to 1,869,386)	296,655 (234,063 to 371,707)	1,659.29 (1,366.3 to 2,012.2)	423.52 (334.16 to 530.67)	−4.3 (−4.66 to −3.94)
Middle SDI	353,833,117 (294,476,333 to 417,960,640)	86,908,066 (70,658,803 to 104,742,712)	176,443.85 (146,844.76 to 208,421.94)	49,207.02 (40,006.75 to 59,304.93)	−4.15 (−4.45 to −3.85)	5,821,677 (4,972,832 to 6,742,399)	1,300,694 (1,096,498 to 1,546,402)	2,903.06 (2,479.77 to 3,362.19)	736.45 (620.83 to 875.57)	−4.45 (−4.73 to −4.16)
Low-middle SDI	436,526,222 (373,423,256 to 496,662,335)	132,445,052 (111,206,358 to 155,228,966)	251,624.87 (215,250.71 to 286,288.87)	69,133.8 (58,047.6 to 81,026.57)	−3.71 (−4.15 to −3.27)	7,189,322 (6,355,803 to 8,108,130)	1,978,347 (1,724,116 to 2,278,399)	4,144.11 (3,663.65 to 4,673.73)	1,032.66 (899.96 to 1,189.28)	−4.05 (−4.49 to −3.6)
Low SDI	253,349,931 (222,268,561 to 281,911,169)	131,195,837 (110,403,315 to 152,409,748)	279,033.44 (244,801.18 to 310,490.1)	79,236.37 (66,678.62 to 92,048.61)	−3.37 (−3.97 to −2.77)	4,315,111 (3,903,527 to 4,743,250)	1,916,883 (1,679,217 to 2,195,389)	4,752.56 (4,299.25 to 5,224.1)	1,157.71 (1,014.17 to 1,325.92)	−3.89 (−4.5 to −3.28)
High-income Asia Pacific	6,882,911 (4,866,472 to 9,311,078)	5,459,346 (3,902,131 to 7,237,515)	67,374.35 (47,636.16 to 91,142.81)	84,612.72 (60,477.92 to 112,172.01)	1.38 (0.93 to 1.83)	106,258 (78,572 to 141,992)	81,519 (61,484 to 105,829)	1,040.13 (769.11 to 1,389.91)	1,263.44 (952.92 to 1,640.22)	1.28 (0.83 to 1.73)
High-income North America	7,545,115 (5,181,665 to 10,576,161)	642,275 (499,750 to 817,522)	34,799.33 (23,898.7 to 48,779.02)	3,133.27 (2,437.97 to 3,988.19)	−7.84 (−8.85 to −6.82)	121,328 (84,137 to 170,863)	10,735 (8,578 to 13,425)	559.59 (388.05 to 788.05)	52.37 (41.84 to 65.49)	−7.9 (−8.89 to −6.9)
Western Europe	19,407,606 (13,551,032 to 26,345,487)	15,502,480 (11,131,295 to 20,392,869)	84,540.02 (59,028.64 to 114,761.61)	73,024.26 (52,433.84 to 96,060.38)	−0.11 (−0.76 to 0.55)	290,323 (210,869 to 396,536)	237,220 (175,147 to 312,801)	1,264.65 (918.55 to 1,727.32)	1,117.42 (825.03 to 1,473.44)	−0.06 (−0.71 to 0.6)
Australasia	507,880 (365,126 to 671,707)	203,334 (151,079 to 266,500)	32,930.58 (23,674.52 to 43,552.97)	11,196.47 (8,319.09 to 14,674.7)	−1.64 (−2.22 to −1.05)	7,685 (5,794 to 10,336)	3,122 (2,440 to 4,065)	498.27 (375.67 to 670.19)	171.89 (134.37 to 223.82)	−1.53 (−2.14 to −0.91)
Andean Latin America	15,756,871 (14,494,989 to 16,953,387)	1,854,614 (1,468,523 to 2,287,471)	298,341.14 (274,448.62 to 320,996.02)	30,127.88 (23,855.9 to 37,159.56)	−7.4 (−8.14 to −6.64)	285,495 (266,165 to 304,140)	26,405 (21,766 to 31,326)	5,405.56 (5,039.58 to 5,758.6)	428.94 (353.58 to 508.88)	−7.97 (−8.7 to −7.23)
Tropical Latin America	28,730,853 (23,019,747 to 34,934,913)	5,813,628 (4,434,811 to 7,392,157)	168,221.48 (134,782.5 to 204,546.76)	33,785.22 (25,772.38 to 42,958.65)	−6.11 (−6.5 to −5.72)	480,762 (398,954 to 572,411)	85,298 (66,820 to 106,347)	2,814.9 (2,335.91 to 3,351.51)	495.7 (388.32 to 618.03)	−6.52 (−6.9 to −6.14)
Central Latin America	32,395,748 (26,553,639 to 38,804,541)	6,445,992 (5,124,297 to 7,889,307)	140,741.46 (115,360.75 to 168,584.09)	32,084.93 (25,506.19 to 39,269.03)	−5 (−5.25 to −4.75)	508,371 (432,749 to 595,721)	95,392 (79,467 to 113,631)	2,208.59 (1,880.05 to 2,588.08)	474.81 (395.55 to 565.6)	−5.18 (−5.41 to −4.94)
Southern Latin America	5,801,009 (4,745,880 to 7,021,947)	1,061,441 (787,147 to 1,382,548)	112,707.95 (92,207.81 to 136,429.58)	24,808.92 (18,397.9 to 32,314.12)	−3.97 (−5.08 to −2.85)	87,174 (73,826 to 102,068)	15,280 (11,868 to 19,168)	1,693.7 (1,434.36 to 1,983.09)	357.15 (277.4 to 448.02)	−4.03 (−5.16 to −2.88)
Caribbean	4,933,546 (4,205,493 to 5,670,210)	1,483,433 (1,211,874 to 1,786,443)	119,415.3 (101,792.96 to 137,246.08)	38,349.64 (31,329.31 to 46,183.04)	−3.32 (−3.81 to −2.82)	74,428 (66,603 to 82,313)	21,177 (18,054 to 24,894)	1,801.51 (1,612.1 to 1,992.35)	547.47 (466.73 to 643.56)	−3.5 (−3.99 to −3.01)
Central Europe	2,067,612 (1,626,373 to 2,595,319)	508,648 (392,608 to 638,051)	22,642.57 (17,810.52 to 28,421.52)	9,106.3 (7,028.84 to 11,423)	−2.67 (−3.12 to −2.22)	35,503 (29,093 to 42,940)	7,795 (6,306 to 9,596)	388.8 (318.6 to 470.24)	139.56 (112.9 to 171.8)	−3.05 (−3.49 to −2.6)
Eastern Europe	20,106,605 (16,009,831 to 25,047,216)	2,635,020 (1,952,430 to 3,399,970)	116,613.14 (92,852.91 to 145,267.41)	26,040.53 (19,294.85 to 33,600.13)	−4.33 (−4.88 to −3.78)	327,481 (267,796 to 401,272)	38,854 (29,901 to 49,232)	1,899.31 (1,553.15 to 2,327.27)	383.97 (295.49 to 486.54)	−4.77 (−5.31 to −4.22)
Central Asia	13,569,353 (11,987,381 to 15,283,989)	1,812,181 (1,489,983 to 2,160,246)	142,467.3 (125,857.87 to 160,469.6)	18,127.2 (14,904.27 to 21,608.89)	−6.68 (−7.05 to −6.3)	230,193 (211,932 to 251,344)	25,981 (22,206 to 30,146)	2,416.84 (2,225.11 to 2,638.9)	259.89 (222.13 to 301.55)	−7.29 (−7.64 to −6.93)
North Africa and Middle East	106,411,110 (92,650,054 to 120,824,076)	32,305,754 (26,760,750 to 37,972,863)	207,714.03 (180,852.51 to 235,848.08)	52,841.49 (43,771.71 to 62,111)	−3.62 (−4.11 to −3.12)	1,834,656 (1,652,365 to 2,027,464)	481,035 (417,791 to 555,114)	3,581.24 (3,225.41 to 3,957.6)	786.81 (683.37 to 907.98)	−4.05 (−4.58 to −3.52)
South Asia	385,246,785 (316,264,985 to 453,056,743)	106,994,261 (88,613,169 to 12,745,5070)	245,339.91 (201,409.66 to 288,523.89)	67,464.36 (55,874.31 to 80,365.75)	−3.58 (−4 to −3.16)	5,969,095 (5,059,963 to 6,980,626)	1,563,595 (1,331,634 to 1,845,412)	3,801.35 (3,222.38 to 4,445.53)	985.91 (839.65 to 1,163.61)	−3.73 (−4.15 to −3.31)
Southeast Asia	144,558,581 (123,914,858 to 163,794,575)	62,745,187 (51,400,445 to 76,002,865)	248,000.47 (212,584.7 to 281,001.18)	111,478.52 (91,322.47 to 135,033.26)	−2.52 (-2.75 to −2.29)	2,506,944 (2,213,598 to 2,794,366)	972,181 (824,830 to 1,160,173)	4,300.84 (3,797.58 to 4,793.93)	1,727.26 (1,465.46 to 2,061.26)	−2.89 (−3.12 to −2.66)
East Asia	120,386,256 (94,929,796 to 151,869,249)	11,805,173 (8,509,481 to 15,840,150)	104,006.49 (82,013.64 to 131,205.9)	14,742.9 (10,627.07 to 19,781.98)	−7.09 (−7.5 to −6.68)	1,995,900 (1,578,934 to 2,480,065)	170,060 (126,489 to 223,611)	1,724.34 (1,364.1 to 2,142.63)	212.38 (157.97 to 279.26)	−7.59 (−8 to −7.18)
Oceania	1,884,484 (1,645,382 to 2,140,065)	2,486,583 (2,074,341 to 2,914,336)	187,663.02 (163,852.48 to 213,114.6)	128,542.07 (107,231.53 to 150,654.43)	−1.49 (−1.7 to −1.28)	30,525 (27,543 to 33,730)	37,569 (32,589 to 43,113)	3,039.81 (2,742.84 to 3,358.9)	1,942.08 (1,684.67 to 2,228.69)	−1.73 (-−1.94 to −1.52)
Western Sub-Saharan Africa	108,088,161 (96715647 to 117,694,048)	69,823,406 (58,810,866 to 81,693,193)	302,392.13 (270,575.89 to 329,265.97)	87,324.99 (73,552.1 to 102,170)	−3.5 (−3.99 to −3.02)	1,881,143 (1,695,781 to 2,050,173)	1,021,638 (895,315 to 1,177,219)	5,262.77 (4,744.19 to 5,735.65)	1,277.72 (1,119.73 to 1,472.29)	−4.12 (−4.6 to −3.64)
Eastern Sub-Saharan Africa	103,603,831 (91,552,402 to 113,911,597)	42,046,037 (36,246,261 to 48,452,569)	287,099.13 (253,703.12 to 315,663.24)	65,906.62 (56,815.54 to 75,948.77)	−4.28 (−5.07 to −3.49)	1,814,230 (1,651,113 to 1,988,477)	621,489 (552,903 to 703,275)	5,027.46 (4,575.44 to 5,510.32)	974.18 (866.67 to 1,102.37)	−4.9 (-5.67 to −4.12)
Central Sub-Saharan Africa	29,582,048 (26,117,743 to 32,538,833)	16,479,501 (13,759,554 to 19,269,154)	284,869.93 (251,509.28 to 313,343.25)	782,26.09 (65,314.85 to 91,468.22)	−3.52 (−4.35 to −2.69)	527,632 (478,257 to 575,017)	237,055 (204,775 to 275,899)	5,081 (4,605.53 to 5,537.31)	1,125.27 (972.04 to 1,309.66)	−4.34 (−5.23 to −3.44)
Southern Sub-Saharan Africa	20,643,922 (17,798,334 to 22,955,093)	4,670,596 (3,873,116 to 5,540,730)	276,250.38 (238,171.64 to 307,177.75)	58,170.76 (4,8238.4 to 69,008)	−4.85 (−5.1 to −4.6)	343,520 (298,063 to 387,092)	71,871 (61,907 to 83,339)	4,596.87 (3,988.59 to 5,179.94)	895.13 (771.03 to 1,037.96)	−5.09 (−5.35 to −4.82)

ASR, age-standardized rate; SDI, sociodemographic index; GBD, Global Burden of Diseases, Injuries, and Risk Factors Study; EAPC, estimated annual percentage change; UIs, uncertainty intervals; CI, confidence interval.

**Table 2:** The case number and ASR of Death and DALYs of infectious diarrhea in children under 5 years of age in 1990 and 2021 for both sexes by SDI quintiles and by GBD regions, with EAPC from 1990 to 2021.

**Location**	**Deaths**					**DALYs**				
	**Number (95 % UIs).1990**	**Number (95 % UIs).2021**	**ASR (95 % UIs).1990**	**ASR (95 % UIs).2021**	**EAPC (95 % CI) 1990–2021**	**Number (95 % UIs).1990**	**Number (95 % UIs).2021**	**ASR (95 % UIs).1990**	**ASR (95 % UIs).2021**	**EAPC (95 % CI) 1990–2021**
Global	1,636,314 (1,285,402 to 1,930,943)	340,429 (250,952 to 464,258)	263.95 (207.34 to 311.47)	51.72 (38.13 to 70.54)	−5.06 (−5.36 to −4.76)	147,785,002 (116,823,041 to 173,680,475)	30,931,280 (23,118,226 to 41,966,936)	23,838.68 (18,844.31 to 28,015.78)	4,699.58 (3,512.49 to 6,376.29)	−5.04 (−5.34 to −4.73)
High SDI	2,089 (1,618 to 2,821)	425 (367 to 480)	3.39 (2.62 to 4.57)	0.79 (0.68 to 0.89)	−4.14 (−4.32 to −3.96)	254,083 (202,409 to 321,586)	76,190 (61,696 to 95,371)	411.72 (327.99 to 521.11)	141.5 (114.58 to 177.12)	−2.8 (−3.02 to −2.59)
High-middle SDI	33,085 (26,377 to 39,776)	1,678 (1,305 to 2,104)	35.61 (28.39 to 42.82)	2.4 (1.86 to 3)	−8.82 (−9.01 to −8.63)	3,124,387 (2,506,993 to 3,709,927)	184,219 (147,038 to 222,584)	3,363.08 (2,698.52 to 3,993.35)	263 (209.92 to 317.77)	−8.28 (−8.43 to −8.14)
Middle SDI	288,482 (222,302 to 342,110)	25,439 (19,432 to 33,425)	143.86 (110.85 to 170.6)	14.4 (11 to 18.93)	−7.13 (−7.28 to −6.99)	26,366,427 (20,552,056 to 31,139,227)	2,417,638 (1,881,869 to 3,098,180)	13,147.99 (10,248.57 to 15,528.01)	1,368.86 (1,065.51 to 1,754.18)	−7.02 (−7.17 to −6.87)
Low-middle SDI	739331 (596347 to 863031)	85502 (64732 to 114557)	426.17 (343.75 to 497.47)	44.63 (33.79 to 59.8)	−6.87 (−7.16 to −6.57)	66,632,099 (53,795,719 to 77,682,088)	7,843,322 (5,981,529 to 10,443,268)	38408.45 (31009.23 to 44777.94)	4094.07 (3122.24 to 5451.19)	−6.81 (−7.1 to −6.51)
Low SDI	572,316 (417,109 to 717,601)	227,079 (160,302 to 315,667)	630.33 (459.39 to 790.35)	137.15 (96.81 to 190.65)	−4.73 (−5.02 to −4.43)	51,316,662 (37,628,480 to 64,249579)	20,382,047 (14,491,345 to 28,195,005)	56,518.92 (41,443.09 to 70,762.92)	12,309.84 (8,752.12 to 17,028.51)	−4.72 (−5.01 to −4.42)
High-income Asia Pacific	173 (144 to 217)	43 (37 to 49)	1.69 (1.41 to 2.12)	0.66 (0.58 to 0.75)	−2.19 (−2.55 to −1.83)	27,819 (22,284 to 35,487)	13,321 (9,777 to 18,648)	272.31 (218.13 to 347.37)	206.46 (151.52 to 289.02)	−0.22 (−0.58 to 0.14)
High-income North America	224 (211 to 238)	116 (101 to 132)	1.03 (0.97 to 1.1)	0.57 (0.49 to 0.65)	−2.03 (−2.6 to −1.46)	34,274 (28,625 to 42,552)	11,656 (10,141 to 13,266)	158.08 (132.02 to 196.26)	56.86 (49.47 to 64.72)	−3.07 (−3.58 to −2.55)
Western Europe	168 (154 to 183)	134 (113 to 155)	0.73 (0.67 to 0.8)	0.63 (0.53 to 0.73)	0.65 (−0.19 to 1.5)	48,915 (35,765 to 68,693)	39,636 (29,568 to 54,280)	213.08 (155.79 to 299.23)	186.7 (139.28 to 255.69)	0.14 (−0.5 to 0.78)
Australasia	14 (12 to 16)	6 (5 to 7)	0.89 (0.78 to 1.01)	0.33 (0.27 to 0.41)	−0.39 (−1.58 to 0.8)	2,118 (1,731 to 2,624)	902 (735 to 1,133)	137.34 (112.21 to 170.14)	49.68 (40.5 to 62.37)	−0.89 (−1.69 to −0.09)
Andean Latin America	7,873 (6,580 to 9,425)	564 (368 to 799)	149.07 (124.59 to 178.45)	9.16 (5.99 to 12.99)	−9.06 (−9.31 to −8.8)	733,006 (616,412 to 866,680)	53,206 (36,050 to 74,222)	13,878.76 (11,671.16 to 16,409.75)	864.33 (585.62 to 1,205.73)	−8.99 (−9.21 to −8.77)
Tropical Latin America	31,553 (26,931 to 36,374)	748 (584 to 946)	184.75 (157.68 to 212.97)	4.35 (3.4 to 5.5)	−11.63 (−11.86 to −11.4)	2,877,930 (2,457,687 to 3,307,394)	76,685 (61,910 to 94,174)	16,850.51 (14,389.96 to 19,365.06)	445.65 (359.78 to 547.28)	−11.38 (−11.58 to −11.17)
Central Latin America	39,758 (36,665 to 43,414)	2,903 (2,109 to 3,872)	172.73 (159.29 to 188.61)	14.45 (10.5 to 19.28)	−7.62 (−8.05 to −7.2)	3,601,608 (3,324,906 to 3,932,793)	269,557 (198,644 to 357,488)	15,646.98 (14,444.86 to 17,085.79)	1,341.72 (988.75 to 1,779.4)	−7.56 (−7.97 to −7.14)
Southern Latin America	860 (790 to 937)	89 (70 to 113)	16.71 (15.34 to 18.2)	2.07 (1.63 to 2.64)	−5.81 (−6.15 to −5.46)	86,979 (79,550 to 94,439)	9,692 (7,797 to 12,151)	1,689.92 (1,545.58 to 1,834.86)	226.52 (182.24 to 284.02)	−5.44 (−5.79 to −5.08)
Caribbean	12,534 (10,280 to 14,893)	3,974 (2,659 to 5,528)	303.39 (248.83 to 360.48)	102.75 (68.75 to 142.91)	−3.13 (−3.61 to −2.65)	1,125,813 (926,617 to 1,335,056)	356,778 (240,071 to 495,586)	27,250.03 (22,428.55 to 32,314.72)	9,223.41 (6,206.31 to 12,811.86)	−3.13 (−3.61 to −2.65)
Central Europe	772 (687 to 868)	185 (151 to 218)	8.46 (7.53 to 9.51)	3.31 (2.7 to 3.9)	−2.65 (−3.82 to −1.47)	73,196 (65,724 to 81,210)	17,471 (14,469 to 20,434)	801.57 (719.75 to 889.34)	312.79 (259.04 to 365.82)	−2.66 (−3.68 to −1.63)
Eastern Europe	1,386 (1,315 to 1,462)	91 (81 to 101)	8.04 (7.62 to 8.48)	0.9 (0.8 to 1)	−8.27 (−8.88 to −7.65)	161,898 (147,089 to 179,379)	12,682 (10,960 to 14,855)	938.96 (853.08 to 1,040.35)	125.33 (108.31 to 146.8)	−7.2 (−7.69 to −6.7)
Central Asia	13,675 (12,328 to 15,244)	1,716 (1,241 to 2,307)	143.58 (129.43 to 160.05)	17.17 (12.41 to 23.07)	−7.69 (−7.99 to −7.39)	1,246,316 (1,123,608 to 1,387,721)	156,035 (112,985 to 208,347)	13,085.32 (11,796.98 to 14,569.95)	1,560.82 (1,130.19 to 2,084.09)	−7.68 (−7.97 to −7.39)
North Africa and Middle East	90,385 (67,646 to 115,927)	11,473 (8,080 to 17,690)	176.43 (132.04 to 226.29)	18.77 (13.22 to 28.93)	−7.16 (−7.42 to −6.89)	8,271,492 (6,236,549 to 10,554,251)	1,079,163 (782,635 to 1,625,428)	16,145.92 (12,173.72 to 20,601.85)	1,765.15 (1,280.13 to 2,658.66)	−7.03 (−7.29 to −6.77)
South Asia	613,477 (489,340 to 736,962)	56,161 (34,503 to 81,505)	390.69 (311.63 to 469.33)	35.41 (21.76 to 51.39)	−7.21 (−7.56 to −6.87)	55,270,939 (44,154,166 to 66,228228)	5,181856 (3,276,948 to 7,440,275)	35,198.65 (28,119.06 to 42,176.67)	3,267.38 (2,066.25 to 4,691.4)	−7.14 (−7.48 to −6.8)
Southeast Asia	170,426 (106,086 to 221,428)	13,698 (10,285 to 17,941)	292.38 (182 to 379.88)	24.34 (18.27 to 31.88)	−7.78 (−7.88 to −7.68)	15,463,620 (9,786,917 to 19,970,273)	1,333,684 (1,019,066 to 1,709,205)	26,528.93 (16,790.15 to 34,260.42)	2,369.54 (1,810.56 to 3,036.72)	−7.57 (−7.66 to −7.47)
East Asia	72,550 (53,672 to 91,813)	795 (602 to 1,091)	62.68 (46.37 to 79.32)	0.99 (0.75 to 1.36)	−13.69 (−14.16 to −13.21)	6,683,248 (5,027,005 to 8,392,858)	90,830 (70,729 to 117,457)	5,773.92 (4,343.03 to 7,250.92)	113.43 (88.33 to 146.69)	−13.1 (−13.56 to −12.63)
Oceania	1,920 (1,337 to 2,819)	1,766 (1,064 to 2,754)	191.18 (133.17 to 280.73)	91.31 (55.02 to 142.36)	−1.82 (−2.05 to −1.59)	174,375 (122,706 to 254,996)	161,464 (99,610 to 249,857)	17,364.81 (12,219.49 to 25,393.35)	8,346.77 (5,149.29 to 12,916.15)	−1.82 (−2.04 to −1.6)
Western Sub-Saharan Africa	289,819 (202,388 to 360,182)	157,796 (106,245 to 230,123)	810.81 (566.21 to 1,007.66)	197.35 (132.88 to 287.8)	−4.36 (−4.75 to −3.96)	25,924,733 (18,175,757 to 32,135,520)	14,121,036 (9,586,422 to 20,521,000)	72,528.16 (50,849.28 to 89,903.72)	17,660.54 (11,989.31 to 25,664.69)	−4.35 (−4.74 to −3.96)
Eastern Sub-Saharan Africa	204,487 (128,877 to 280,209)	66,667 (46,246 to 96,386)	566.66 (357.13 to 776.49)	104.5 (72.49 to 151.08)	−5.33 (−5.5 to −5.16)	18,376,539 (11,668,580 to 25,086,522)	5,994,967 (4,175,442 to 8,646,755)	50,923.68 (32,335.09 to 69,517.88)	9,397.03 (6,544.95 to 13,553.68)	−5.32 (−5.5 to −5.15)
Central Sub-Saharan Africa	60,617 (42,180 to 76,496)	13,404 (8,218 to 20,759)	583.73 (406.19 to 736.64)	63.63 (39.01 to 98.54)	−6.73 (−7.69 to −5.77)	5,452,145 (3,802,717 to 6,858,692)	1,220,197 (757,095 to 1,880,530)	52,503.2 (36,619.49 to 66,048)	5,792.12 (3,593.83 to 8,926.64)	−6.69 (−7.65 to −5.73)
Southern Sub-Saharan Africa	23,642 (20,416 to 27,215)	8,101 (5,953 to 10,797)	316.37 (273.2 to 364.18)	100.89 (74.14 to 134.47)	−3.31 (−3.82 to −2.8)	2,148,040 (1,855,248 to 2,468,975)	730,458 (539,617 to 969,537)	28,744.38 (24,826.34 to 33,039.04)	9,097.62 (6,720.75 to 12,075.27)	−3.34 (−3.85 to −2.83)

ASR, age-standardized rate; DALYs, disability-adjusted life-years; SDI, sociodemographic index; GBD, Global Burden of Diseases, Injuries, and Risk Factors Study; EAPC, estimated annual percentage change; UIs, uncertainty intervals; CI, confidence interval.

Nationally, substantial variation in disease burden was evident. India reported the most incident and prevalent cases, while Nigeria recorded the highest deaths and DALYs. The Netherlands showed the highest ASRs for incidence and prevalence, whereas Chad had the highest mortality and DALY ASRs for children under five (Figure 1, Supplementary Table 1). When grouped by SDI quintiles, in 2021, the low-middle SDI group had the greatest numbers of incident and prevalent cases, while the low SDI group recorded the highest deaths and DALYs. ASRs for all four indicators peaked in the low SDI quintile (Tables 1, 2).

### Temporal trends and EAPC estimates

Globally, significant reductions in ASRs for childhood infectious diarrhea were observed from 1990 to 2021. The estimated annual percentage changes (EAPCs) for incidence and prevalence ASRs were −3.34 (95% CI: −3.82 to −2.86) and −3.7 (95% CI: −4.18 to −3.22), respectively, while mortality and DALY ASRs declined at EAPCs of −5.06 (95% CI: −5.36 to −4.76) and −5.04 (95% CI: −5.34 to −4.73). Regionally, the largest drop in incidence ASR occurred in High-income North America, while High-income Asia Pacific experienced minimal or even increasing trends. Prevalence ASR fell most rapidly in Andean Latin America and least in High-income Asia Pacific. East Asia saw the steepest declines in mortality and DALY ASRs; Western Europe had the smallest reductions in these metrics (Tables 1, 2).

At the country level, Paraguay had the greatest decline in incidence (EAPC: −9.9), and Taiwan (Province of China) had the largest increase (EAPC: 3.23). For prevalence, Paraguay again led the decline (EAPC: −10.82), with Taiwan posting the greatest increase (EAPC: 2.88). The steepest drop in mortality was recorded in Kazakhstan (EAPC: −14.55), while Sweden exhibited an increase (EAPC: 8.33). Kazakhstan also had the fastest reduction in DALY ASR (EAPC: −14.08), while the Netherlands saw the highest increase (EAPC: 2.09). Across all SDI groups, ASRs for mortality and DALYs declined, with the greatest decreases in the high-middle SDI quintile, and the smallest in the high SDI quintile.

### Joinpoint regression analysis

**Figure 2:**
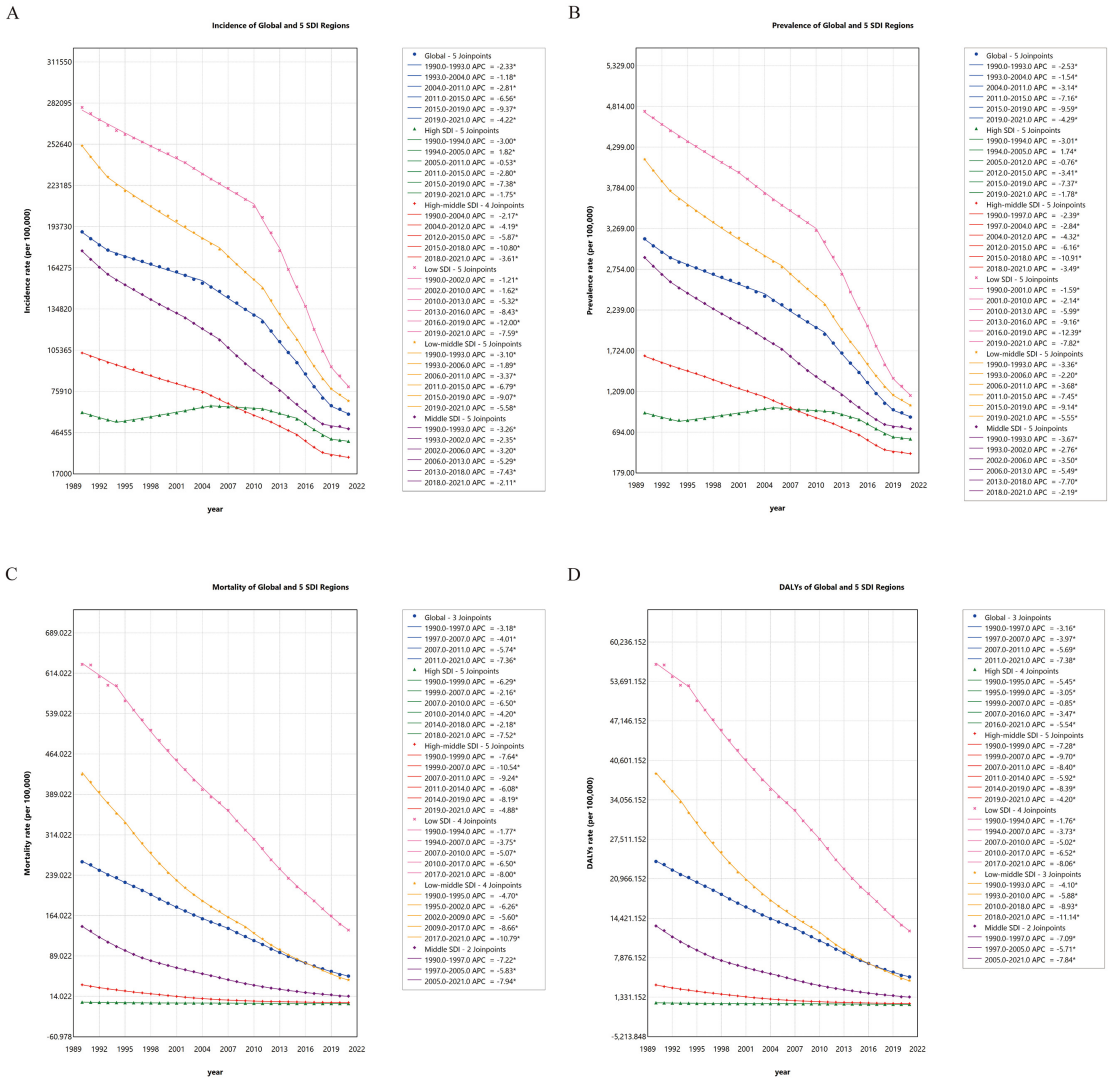
**(A)** Joinpoint regression analysis of ASIR in global and different SDI regions from 1990 to 2021; **(B)** Joinpoint regression analysis of ASPR in global and different SDI regions from 1990 to 2021; **(C)** Joinpoint regression analysis of ASMR in global and different SDI regions from 1990 to 2021; **(D)** Joinpoint regression analysis of ASDR in global and different SDI regions from 1990 to 2021. ASIR, Age-standardized incidence rate; ASPR, Age-standardized prevalence rate; ASMR, Age-standardized mortality rate; ASDR, Age-standardized disability-adjusted life-year rate; APC, Annual Percent Change; AAPC, Average Annual Percent Change; SDI, Sociodemographic Index.

Joinpoint regression indicated that global incidence, prevalence, mortality, and DALY rates in children under five consistently decreased from 1990 to 2021, with the most rapid reductions in incidence and prevalence during 2015–2019, and in mortality and DALYs during 2011–2021 (Figure 2). Regional analysis revealed temporal heterogeneity in these trends. In low and low-middle SDI regions, the sharpest drops in incidence and prevalence occurred between 2015–2019, and for mortality and DALYs in 2017–2021. Middle SDI regions experienced the greatest declines in incidence and prevalence between 2013–2018, and in mortality and DALYs from 2005–2021. High-middle SDI areas had significant reductions from 2015–2018 (incidence and prevalence) and 1999–2007 (mortality and DALYs). High SDI regions saw increases in incidence and prevalence between 1994–2005, followed by marked decreases in 2015–2019, and mortality and DALY rates fell sharply from 2018–2021 (Supplementary Tables 3–6).

### Inequality analysis

**Figure 3:**
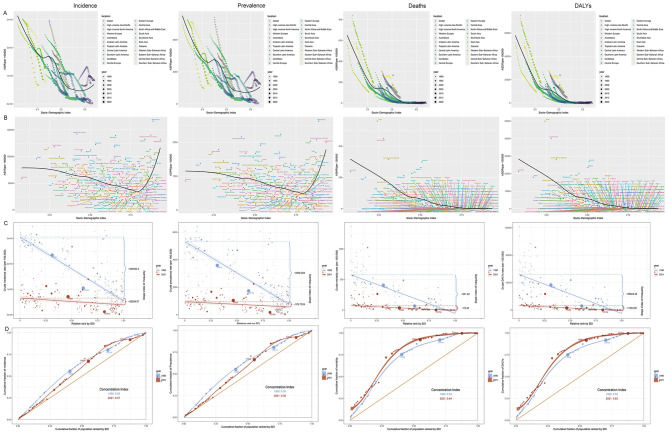
Global burden of infectious diarrhea among children under five in relation to sociodemographic index (SDI), 1990–2021. **(A)** Relationship between SDI and ASIR, ASPR, ASMR, and ASDR by world regions in 2021; **(B)** Relationship between SDI and ASIR, ASPR, ASMR, and ASDR by countries in 2021; **(C)** SDI-related health inequality regression curves for incidence, prevalence, mortality and DALYs, 1990 and 2021; **(D)** SDI-related concentration curves for incidence, prevalence, mortality, and DALYs, 1990 and 2021. ASR, age-standardized rate; ASIR, Age-standardized incidence rate; ASPR, Age-standardized prevalence rate; ASMR, Age-standardized mortality rate; ASDR, Age-standardized disability-adjusted life-year rate; EAPC, estimated annual percentage change; DALYs, disability-adjusted life-years; SDI, Sociodemographic Index.

Globally and across 21 GBD regions, nonlinear relationships were evident between SDI and age-standardized incidence, prevalence, mortality, and DALY rates of infectious diarrhea in young children (Figure 3). Below an SDI of 0.8, increasing SDI corresponded to markedly lower incidence and prevalence; above 0.8, these rates trended upward as SDI increased. Mortality and DALYs, by contrast, consistently declined with rising SDI. At the national level, ASMR and ASDR generally increased with SDI, but incidence and prevalence also rose with SDI above 0.8, while mortality and DALYs continued to drop. The majority of deaths and DALYs persisted in countries of lower sociodemographic development.

Measures of inequality further emphasized these trends. The 2021 Slope Index of Inequality (SII) for incidence, prevalence, mortality, and DALYs were −26,298.97, −378.75, −79.45, and −7,189.58, respectively, all notably lower than in 1990. The concentration indices of incidence and prevalence showed slight reductions, while those for mortality and DALYs increased, highlighting persistent and sometimes widening disparities in disease burden across SDI levels.

### Frontier analysis

**Figure 4:**
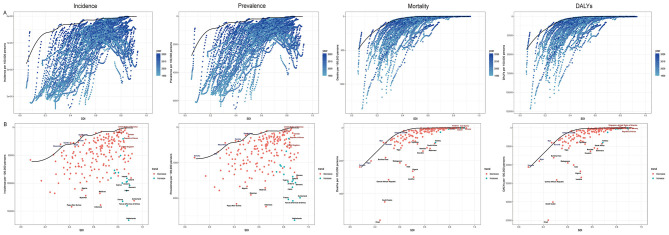
**(A)** Frontier analysis of SDI and infectious diarrhea among children under five incidence, prevalence, mortality and DALYs rate from 1990 to 2021; **(B)** Frontier analysis of SDI and infectious diarrhea among children under five incidence, prevalence, mortality and DALYs rate in 1990 and 2021. ASR, age-standardized rate; ASIR, Age-standardized incidence rate; ASPR, Age-standardized prevalence rate; ASMR, Age-standardized mortality rate; ASDR, Age-standardized disability-adjusted life-year rate; EAPC, estimated annual percentage change; DALYs, disability-adjusted life-years; SDI, Sociodemographic Index.

Frontier analysis across 204 countries and territories from 1990 to 2021 showed decreasing trends in incidence, prevalence, mortality, and DALYs, regardless of SDI. Mortality and DALY rates converged with increasing SDI, whereas incidence and prevalence converged up to an SDI of 0.7 but diverged at higher SDI levels (Figure 4). In 2021, several countries, such as Papua New Guinea, the Netherlands, and Indonesia, featured notably high incidence and prevalence relative to their SDI, while Bangladesh, Gambia, and Mozambique were closer to the efficiency frontier, indicating better-than-expected performance. For mortality and DALYs, Chad, South Sudan, and the Central African Republic were among the poorest performers, whereas the Solomon Islands, Burundi, and Mali achieved outcomes near the frontier.

### Pathogen-specific DALY trends

**Figure 5:**
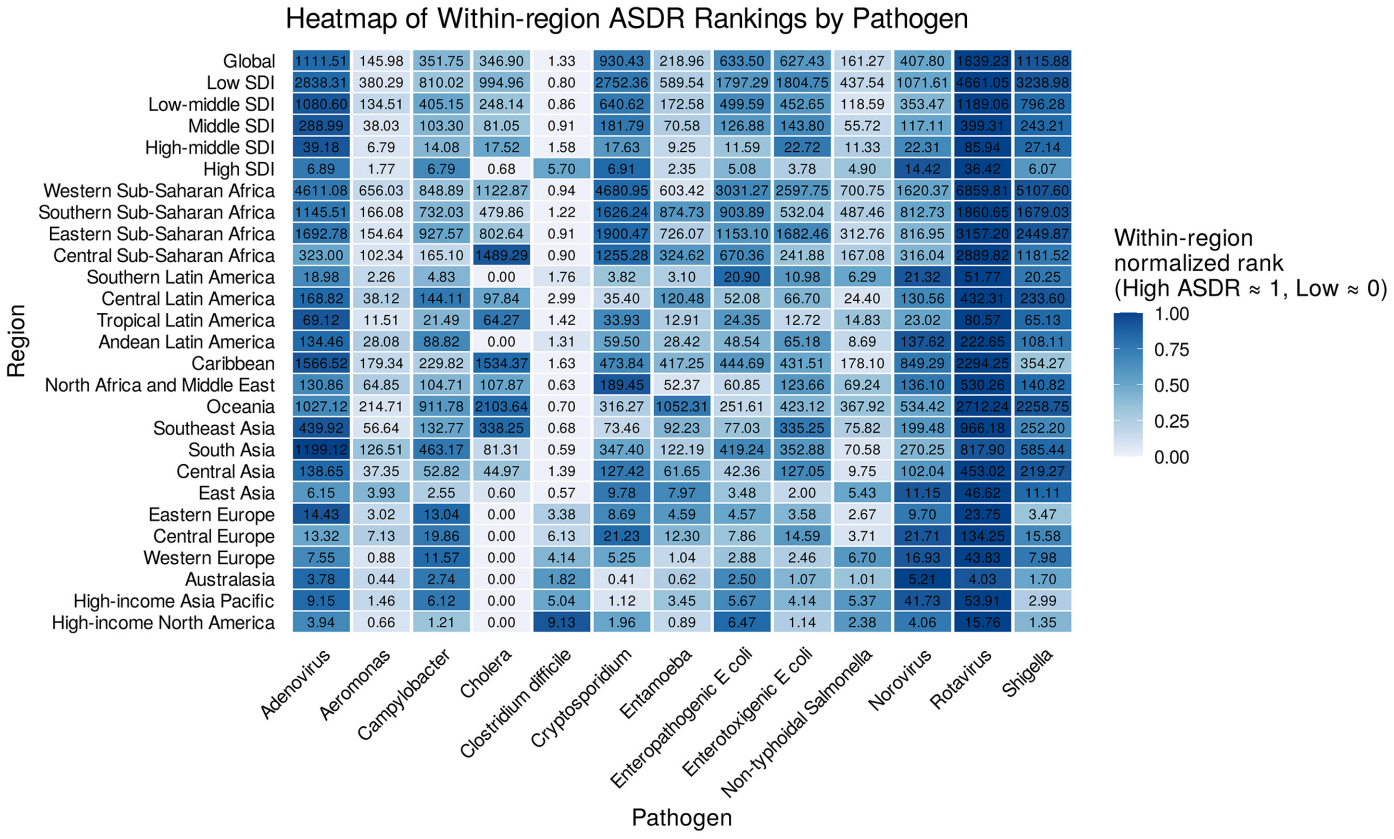
Heatmap of within-region DALY rankings by pathogen. DALYs, disability-adjusted life-years; SDI, Sociodemographic Index.

Assessment of DALY rates by pathogen showed an overall decrease worldwide from 1990 to 2021, with strong regional and etiological variation (Figure 5). The highest DALY rates were observed in South Asia, Sub-Saharan Africa, and Southeast Asia, where rotavirus, Shigella, Campylobacter, and EPEC dominated. In higher income regions, overall DALY rates were much lower, and viral etiologies, such as norovirus, rotavirus, and sapovirus, predominated. Adenovirus was a significant contributor in some regions, notably South Asia.

Regionally, rotavirus, Shigella, norovirus, and Campylobacter were consistently among the leading pathogens. Rotavirus was typically ranked first in developing regions, while norovirus emerged as the top pathogen in high-income contexts. Bacterial causes posed major threats in lower-income settings, and certain agents—like adenovirus—had prominent roles in specific geographies (Supplementary Table 7).

In terms of temporal trends, EAPCs for DALY rates due to most pathogens were negative, reflecting declines, particularly in locations with improved vaccination and sanitation (e.g., South Asia, Sub-Saharan Africa). Declines were marked for rotavirus and Shigella. However, in some high-income areas, DALY rates for viral pathogens (norovirus, sapovirus) remained stable or increased slightly, possibly as bacterial diarrhea became better controlled. In selected settings—such as South Asia—the decrease in adenovirus DALY rates was limited or even reversed in some countries (Supplementary Table 8).

## Discussion

This comprehensive assessment of the burden and trends of infectious diarrhea among children under five highlights both remarkable global progress and persistent, complex challenges. Our findings demonstrate marked declines in ASRs for incidence, prevalence, mortality, and DALYs from 1990 to 2021, underscoring the impact of intensified interventions in public health, expanded immunization programs, and improved sanitation. However, the overall case numbers and DALYs have increased, largely driven by population growth in low- and middle-income settings. These contrasting trends point to the dual necessity of sustaining current progress and addressing the unmet needs generated by demographic expansion.

Critically, our analysis reveals pronounced geographic, socioeconomic, and temporal disparities. South Asia and Western Sub-Saharan Africa continue to bear the heaviest burden, with the former leading in case counts and the latter showing the highest mortality and DALY rates. At the national level, India and Nigeria remain epicenters for cases and deaths, while certain high-income countries such as the Netherlands demonstrate the highest ASRs for morbidity—an observation that may reflect differences in healthcare-seeking behavior, reporting practices, and diagnostic capacity.

The observed nonlinear associations between SDI and burden indicators add depth to our understanding (Figure 3). Below an SDI of 0.8, increasing SDI corresponded with sharply reduced incidence and prevalence, likely a result of improved water, sanitation, and immunization coverage. Yet, at higher SDI levels, incidence and prevalence display a rise—possibly attributable to more sensitive case ascertainment, greater health system utilization, or genuine increases related to lifestyle and childcare environments in affluent societies. Importantly, mortality and DALY rates continue a steady decline with increasing SDI, reflecting the paramount importance of health service access and quality in mitigating the most severe outcomes. These patterns are further linked to pathogen-specific trends: in low-SDI regions, bacterial pathogens like Shigella and Campylobacter dominate DALYs, potentially exacerbated by confounding factors such as limited diagnostic capacity, inadequate WASH infrastructure, and socioeconomic barriers to care, which hinder timely interventions. In contrast, high-SDI settings show a shift toward viral etiologies (e.g., norovirus), where confounders like advanced surveillance and higher healthcare-seeking rates may inflate reported morbidity without corresponding mortality increases. Such variations underscore the need to account for these factors in interpreting SDI-burden relationships, as they may mask true epidemiological shifts or amplify apparent disparities.

Temporal trend analysis—particularly joinpoint regression—revealed periods of accelerated progress, often aligning with key interventions. Notably, the mid-2010s saw rapid declines in incidence and prevalence, likely driven by the global rollout of the rotavirus vaccine, expansion of integrated management of childhood illness (IMCI) programs, and increased investment in WASH (water, sanitation, and hygiene). While encouraging, our findings of variable trends within high-income regions, including increases in incidence in places like the High-income Asia Pacific and certain European countries, emphasize the changing epidemiological landscape and the need for ongoing surveillance and adaptable intervention strategies.

Despite absolute declines in global inequality—the gap in rates—our data show a persistent, and in some cases widening, relative disparity for mortality and DALY burden as measured by concentration indices. This suggests that while overall improvements have been achieved, children in the least developed environments continue to shoulder a disproportionate risk, underscoring the inadequacy of mean-based progress as a measure of equity. These patterns highlight the need for policies prioritizing the poorest and most marginalized groups, and for tracking both absolute and relative measures of health equity.

Frontier analyses provided an innovative benchmarking approach, revealing outliers both above and below expected performance for a given SDI (Figure 4). Countries such as the Netherlands and Papua New Guinea, with higher-than-expected incidence and prevalence, may warrant further investigation into healthcare reporting, pathogen profiles, or health system factors. Conversely, nations like Bangladesh and the Gambia perform near the theoretical optimum for their development context, providing potential models for best practice. For mortality and DALYs, convergence among higher SDI countries demonstrates the attainability of reductions; persistent outliers among lower SDI nations, such as Chad and South Sudan, highlight urgent need for targeted intervention.

Pathogen-specific analyses reinforce the dynamic nature of the diarrheal disease landscape. The decline of rotavirus- and Shigella-related DALY rates in many regions reflects the efficacy of vaccines and hygiene improvements. Yet, the persistence or even increase of viral pathogens such as norovirus and sapovirus—especially in high-income settings—signals a shift in etiologic dominance, likely as bacterial diarrhea becomes better controlled. The persistent burden of adenovirus in South Asia points to gaps in hygiene or the absence of effective vaccines, and highlights the importance of continued pathogen surveillance and the need for next-generation vaccine and prevention strategies.

A key challenge amplifying morbidity and mortality in LMICs, particularly in high-burden regions like Sub-Saharan Africa and South Asia, is the overuse of antibiotics for childhood diarrhea, often without prior use of evidence-based treatments such as oral rehydration therapy (33–36). This practice, driven by factors including over-the-counter availability and limited regulatory oversight, contributes to antimicrobial resistance (AMR), prolonged illness, and poorer outcomes, especially in settings with inadequate WASH infrastructure. For instance, in countries like Kenya and Ethiopia, inappropriate antibiotic prescribing exacerbates bacterial pathogen burdens, as seen in our pathogen-specific DALY trends for Shigella and Campylobacter. To address AMR globally, with emphasis on LMICs in Africa and Asia, strategies should prioritize antibiotic stewardship programs, community education on rational use, and integration with WASH initiatives to reduce infection incidence at the source.

These findings have direct implications for health policies, particularly in low-SDI regions where burdens remain highest. For key LMICs such as India, Nigeria, and those in Western Sub-Saharan Africa, targeted interventions should include scaling up rotavirus vaccination to sustain EAPC-driven declines, enhancing WASH infrastructure to address nonlinear SDI patterns, and implementing pathogen-specific surveillance to counter shifts toward persistent bacterial and emerging viral etiologies. In low-SDI settings, policies could integrate IMCI with community-based antibiotic stewardship to mitigate overuse and AMR risks, while frontier analysis highlights the value of emulating efficient performers like Bangladesh through equitable resource allocation. For middle-SDI Asian countries facing rising absolute burdens, adaptive strategies might focus on urban sanitation improvements and vaccine equity. Limited emphasis on high-income countries should center on surveillance for viral shifts, but overall, LMIC-focused investments—such as multilateral funding for WASH and AMR control—could accelerate progress and reduce inequalities.

Our study has several strengths, notably the comprehensive scope spanning 204 countries and multiple decades, the use of harmonized GBD methodology, and detailed disaggregation by SDI and pathogen. Nonetheless, certain limitations warrant consideration. GBD estimates rely on available data, which may be sparse or of variable quality in many low-resource settings, potentially leading to uncertainty in rates and trends. Attribution of burden by pathogen is constrained by diagnostic capacity and surveillance data. Additionally, SDI—though a valuable proxy—may not fully capture all context-specific determinants of diarrheal disease burden, including conflict, migration, or local health policies.

In conclusion, while the global fight against childhood infectious diarrhea has achieved substantial success, this progress remains fragile and uneven. Sustaining gains will require continued investment in immunization, WASH, and health systems strengthening, with renewed emphasis on equity to close persistent gaps. Emerging shifts in pathogen patterns and heterogeneous national trajectories argue for adaptive, data-informed strategies. Policymakers should prioritize reaching the most disadvantaged, invest in pathogen surveillance, and support research into novel vaccines and interventions, ensuring that recent progress translates into sustained and inclusive child health gains worldwide.

## Data Availability

Publicly available datasets were analyzed in this study. This data can be found here: https://vizhub.healthdata.org/gbd-results.
