# Supplementary material for: Global trends, inequalities, and pathogen shifts in infectious diarrhea among children under five: a comprehensive analysis of the global burden of disease study 1990–2021
Source: Front Nutr. 2025 Nov 14;12:1679081. doi: 10.3389/fnut.2025.1679081 (PMC12661344; doi:10.3389/fnut.2025.1679081)
Supplement: Supplementary file 1 [file Table_1.docx]

**Table S1. The case number and ASR of Incidence and prevalence of infectious diarrhea in children under 5 years of age in 1990 and 2021 for both sexes by SDI quintiles, by GBD regions and 204 countries and regions, with EAPC from 1990 to 2021.**

|  | **SDI** | | **Incidence** | | | | | **Prevalence** | | | | |
| --- | --- | --- | --- | --- | --- | --- | --- | --- | --- | --- | --- | --- |
| **Location** | **SDI.Index.Value** | **SDI.Quintile** | **Number (95 % UIs).1990** | **Number (95 % UIs).2021** | **ASR (95 % UIs) .1990** | **ASR (95 % UIs) .2021** | **EAPC (95 % CI) 1990–2021** | **Number (95 % UIs).1990** | **Number (95 % UIs).2021** | **ASR (95 % UIs) .1990** | **ASR (95 % UIs) .2021** | **EAPC (95 % CI) 1990–2021** |
| **Global** | **0.666368** | **NA** | **1178110288 (1000354993 to 1366541108)** | **392778890 (324124687 to 463633235)** | **190036.82 (161363.74 to 220431.93)** | **59677.27 (49246.22 to 70442.6)** | **-3.34 (-3.82 to -2.86)** | **19458645 (17043262 to 22054325)** | **5825270 (4975326 to 6775131)** | **3138.81 (2749.19 to 3557.51)** | **885.07 (755.93 to 1029.39)** | **-3.7 (-4.18 to -3.22)** |
| **High SDI** | **NA** | **NA** | **37611903 (27244562 to 50267709)** | **21691430 (15513335 to 28673257)** | **60947.41 (44147.87 to 81455.25)** | **40284.05 (28810.45 to 53250.29)** | **-0.67 (-1.14 to -0.21)** | **579741 (437163 to 771923)** | **327606 (245544 to 430230)** | **939.43 (708.39 to 1250.85)** | **608.41 (456.01 to 799)** | **-0.75 (-1.21 to -0.29)** |
| **High-middle SDI** | **NA** | **NA** | **96107437 (77466420 to 117910531)** | **20200475 (15302775 to 25985679)** | **103449.61 (83384.5 to 126918.36)** | **28839.35 (21847.11 to 37098.64)** | **-4 (-4.38 to -3.61)** | **1541524 (1269328 to 1869386)** | **296655 (234063 to 371707)** | **1659.29 (1366.3 to 2012.2)** | **423.52 (334.16 to 530.67)** | **-4.3 (-4.66 to -3.94)** |
| **Middle SDI** | **NA** | **NA** | **353833117 (294476333 to 417960640)** | **86908066 (70658803 to 104742712)** | **176443.85 (146844.76 to 208421.94)** | **49207.02 (40006.75 to 59304.93)** | **-4.15 (-4.45 to -3.85)** | **5821677 (4972832 to 6742399)** | **1300694 (1096498 to 1546402)** | **2903.06 (2479.77 to 3362.19)** | **736.45 (620.83 to 875.57)** | **-4.45 (-4.73 to -4.16)** |
| **Low-middle SDI** | **NA** | **NA** | **436526222 (373423256 to 496662335)** | **132445052 (111206358 to 155228966)** | **251624.87 (215250.71 to 286288.87)** | **69133.8 (58047.6 to 81026.57)** | **-3.71 (-4.15 to -3.27)** | **7189322 (6355803 to 8108130)** | **1978347 (1724116 to 2278399)** | **4144.11 (3663.65 to 4673.73)** | **1032.66 (899.96 to 1189.28)** | **-4.05 (-4.49 to -3.6)** |
| **Low SDI** | **NA** | **NA** | **253349931 (222268561 to 281911169)** | **131195837 (110403315 to 152409748)** | **279033.44 (244801.18 to 310490.1)** | **79236.37 (66678.62 to 92048.61)** | **-3.37 (-3.97 to -2.77)** | **4315111 (3903527 to 4743250)** | **1916883 (1679217 to 2195389)** | **4752.56 (4299.25 to 5224.1)** | **1157.71 (1014.17 to 1325.92)** | **-3.89 (-4.5 to -3.28)** |
| **East Asia** | **0.722912** | **NA** | **120386256 (94929796 to 151869249)** | **11805173 (8509481 to 15840150)** | **104006.49 (82013.64 to 131205.9)** | **14742.9 (10627.07 to 19781.98)** | **-7.09 (-7.5 to -6.68)** | **1995900 (1578934 to 2480065)** | **170060 (126489 to 223611)** | **1724.34 (1364.1 to 2142.63)** | **212.38 (157.97 to 279.26)** | **-7.59 (-8 to -7.18)** |
| **China** | **0.718679** | **NA** | **117027013 (92087701 to 147730219)** | **9246102 (6612630 to 12615856)** | **104669.68 (82363.81 to 132130.82)** | **11904.6 (8513.94 to 16243.25)** | **-7.97 (-8.48 to -7.46)** | **1939330 (1531088 to 2413677)** | **132918 (97626 to 179397)** | **1734.55 (1369.41 to 2158.81)** | **171.14 (125.7 to 230.98)** | **-8.48 (-9 to -7.96)** |
| **Democratic People's Republic of Korea** | **0.569455** | **Low-middle SDI** | **2144450 (1729712 to 2613019)** | **1307667 (1027168 to 1631158)** | **91787.62 (74035.81 to 111843.48)** | **86421.92 (67884.07 to 107800.96)** | **0.56 (-0.24 to 1.36)** | **35964 (29810 to 43162)** | **18622 (15142 to 22672)** | **1539.35 (1275.95 to 1847.43)** | **1230.68 (1000.69 to 1498.36)** | **0.05 (-0.76 to 0.86)** |
| **Taiwan (Province of China)** | **0.87514** | **High SDI** | **1214793 (929923 to 1569101)** | **1251404 (879560 to 1678912)** | **75619.51 (57886.66 to 97674.74)** | **140255.15 (98579.6 to 188169.55)** | **3.23 (2.71 to 3.75)** | **20607 (16273 to 25675)** | **18519 (13121 to 24855)** | **1282.74 (1012.96 to 1598.22)** | **2075.62 (1470.56 to 2785.73)** | **2.88 (2.32 to 3.44)** |
| **Southeast Asia** | **0.649072** | **NA** | **144558581 (123914858 to 163794575)** | **62745187 (51400445 to 76002865)** | **248000.47 (212584.7 to 281001.18)** | **111478.52 (91322.47 to 135033.26)** | **-2.52 (-2.75 to -2.29)** | **2506944 (2213598 to 2794366)** | **972181 (824830 to 1160173)** | **4300.84 (3797.58 to 4793.93)** | **1727.26 (1465.46 to 2061.26)** | **-2.89 (-3.12 to -2.66)** |
| **Cambodia** | **0.474** | **Low-middle SDI** | **4701155 (4005315 to 5328958)** | **1225387 (1042554 to 1424519)** | **257591.64 (219464.27 to 291990.98)** | **70080.36 (59624.06 to 81468.79)** | **-4.31 (-5.06 to -3.55)** | **84079 (73332 to 94083)** | **18074 (15787 to 20854)** | **4606.95 (4018.12 to 5155.11)** | **1033.66 (902.89 to 1192.65)** | **-5.06 (-5.81 to -4.3)** |
| **Indonesia** | **0.657935** | **NA** | **59659692 (49730308 to 68740795)** | **30472024 (24631346 to 36968093)** | **267283.17 (222798.24 to 307967.69)** | **139099.98 (112438.21 to 168753.51)** | **-1.72 (-1.99 to -1.45)** | **1048424 (896721 to 1196040)** | **490117 (415832 to 585200)** | **4697.08 (4017.43 to 5358.42)** | **2237.31 (1898.21 to 2671.34)** | **-2 (-2.27 to -1.73)** |
| **Lao People's Democratic Republic** | **0.489281** | **Low-middle SDI** | **2027098 (1880265 to 2163843)** | **822099 (695023 to 956620)** | **285123.21 (264470.24 to 304357.15)** | **99103.79 (83784.83 to 115320.33)** | **-3.31 (-3.59 to -3.03)** | **38773 (35775 to 41261)** | **12844 (11338 to 14548)** | **5453.58 (5031.97 to 5803.6)** | **1548.34 (1366.75 to 1753.72)** | **-4.02 (-4.27 to -3.78)** |
| **Malaysia** | **0.742553** | **High-middle SDI** | **2058379 (1662464 to 2491540)** | **2233559 (1584596 to 2979018)** | **86401.51 (69782.75 to 104583.67)** | **90853.59 (64455.98 to 121176.32)** | **0.74 (0.44 to 1.03)** | **29674 (24705 to 35519)** | **30942 (22536 to 41013)** | **1245.58 (1037.02 to 1490.92)** | **1258.62 (916.68 to 1668.28)** | **0.66 (0.34 to 0.98)** |
| **Maldives** | **0.657665** | **Middle SDI** | **106491 (92311 to 118304)** | **37570 (31205 to 44491)** | **254875.65 (220938.01 to 283149.63)** | **118298.84 (98257.73 to 140093.93)** | **-2.72 (-2.83 to -2.61)** | **1946 (1709 to 2178)** | **543 (466 to 635)** | **4657.35 (4090.05 to 5213.22)** | **1710.27 (1467.95 to 1998.78)** | **-3.48 (-3.64 to -3.32)** |
| **Myanmar** | **0.528492** | **Low-middle SDI** | **14006231 (12872989 to 15030712)** | **6393490 (5495108 to 7333879)** | **277779.12 (255304.06 to 298097.17)** | **122330.42 (105141.14 to 140323.42)** | **-3.11 (-3.44 to -2.78)** | **269647 (249501 to 289732)** | **99772 (88944 to 111966)** | **5347.79 (4948.23 to 5746.12)** | **1908.99 (1701.83 to 2142.32)** | **-3.92 (-4.29 to -3.56)** |
| **Philippines** | **0.65192** | **NA** | **27485347 (23617024 to 30680678)** | **10921647 (8602752 to 13572289)** | **297101.98 (255287.47 to 331641.82)** | **97398.34 (76718.62 to 121036.54)** | **-3.99 (-4.37 to -3.62)** | **473307 (414891 to 530523)** | **169798 (137707 to 208401)** | **5116.2 (4484.76 to 5734.67)** | **1514.24 (1228.06 to 1858.5)** | **-4.32 (-4.67 to -3.97)** |
| **Sri Lanka** | **0.701372** | **Middle SDI** | **2606099 (2168017 to 3062395)** | **1078882 (796404 to 1403488)** | **147417.13 (122636.48 to 173228.06)** | **68911.48 (50868.76 to 89645.03)** | **-2.56 (-2.77 to -2.35)** | **38555 (33653 to 43735)** | **14893 (11116 to 19151)** | **2180.93 (1903.64 to 2473.91)** | **951.28 (710.03 to 1223.25)** | **-2.75 (-2.98 to -2.53)** |
| **Thailand** | **0.682657** | **Middle SDI** | **10432750 (8705904 to 12138378)** | **1857346 (1533032 to 2219426)** | **200619.36 (167412.52 to 233418.21)** | **65699.57 (54227.66 to 78507.35)** | **-3.65 (-3.99 to -3.31)** | **168879 (146663 to 192810)** | **26714 (22758 to 31245)** | **3247.51 (2820.3 to 3707.69)** | **944.96 (805.02 to 1105.24)** | **-3.97 (-4.3 to -3.64)** |
| **Timor-Leste** | **0.450689** | **Low SDI** | **400067 (359519 to 433458)** | **203648 (167830 to 238556)** | **285660.56 (256707.81 to 309502.72)** | **110170.23 (90793.16 to 129054.57)** | **-2.73 (-3.19 to -2.28)** | **7457 (6656 to 8175)** | **3092 (2666 to 3541)** | **5324.84 (4752.3 to 5836.92)** | **1672.45 (1442.52 to 1915.8)** | **-3.44 (-3.91 to -2.98)** |
| **Viet Nam** | **0.621621** | **Middle SDI** | **20618421 (16696458 to 24676433)** | **7347801 (5524218 to 9295533)** | **219126.71 (177445.2 to 262254.09)** | **90248.02 (67850.2 to 114170.69)** | **-2.81 (-2.94 to -2.69)** | **338428 (282381 to 403648)** | **103114 (80713 to 129237)** | **3596.71 (3001.06 to 4289.86)** | **1266.47 (991.34 to 1587.34)** | **-3.27 (-3.42 to -3.13)** |
| **Oceania** | **0.467359** | **NA** | **1884484 (1645382 to 2140065)** | **2486583 (2074341 to 2914336)** | **187663.02 (163852.48 to 213114.6)** | **128542.07 (107231.53 to 150654.43)** | **-1.49 (-1.7 to -1.28)** | **30525 (27543 to 33730)** | **37569 (32589 to 43113)** | **3039.81 (2742.84 to 3358.9)** | **1942.08 (1684.67 to 2228.69)** | **-1.73 (-1.94 to -1.52)** |
| **Fiji** | **0.669069** | **Middle SDI** | **110468 (91447 to 133413)** | **82377 (67963 to 98144)** | **117220.38 (97036.85 to 141567.47)** | **90479.06 (74647.56 to 107796.41)** | **-0.6 (-0.78 to -0.42)** | **1623 (1377 to 1917)** | **1206 (1044 to 1419)** | **1721.71 (1461.69 to 2034.61)** | **1324.17 (1146.17 to 1558.08)** | **-0.58 (-0.77 to -0.4)** |
| **Kiribati** | **0.525958** | **Low-middle SDI** | **24203 (20386 to 28266)** | **9702 (8034 to 11516)** | **205527.69 (173108.79 to 240031.34)** | **67884.87 (56210.01 to 80570.74)** | **-3.76 (-4.03 to -3.48)** | **382 (331 to 443)** | **140 (119 to 161)** | **3247.21 (2809.49 to 3761.98)** | **979.04 (832.63 to 1128.84)** | **-4.06 (-4.34 to -3.79)** |
| **Marshall Islands** | **0.573525** | **Low-middle SDI** | **9022 (7249 to 10876)** | **2516 (2019 to 3066)** | **121308.13 (97464.38 to 146238.91)** | **44346.77 (35581.92 to 54033.5)** | **-4.2 (-4.8 to -3.59)** | **137 (114 to 162)** | **35 (29 to 42)** | **1836.06 (1534.47 to 2173.67)** | **617.1 (512.58 to 733.98)** | **-4.52 (-5.16 to -3.89)** |
| **Micronesia (Federated States of)** | **0.588013** | **Low-middle SDI** | **21270 (17077 to 26111)** | **4137 (3240 to 5127)** | **135834.99 (109056.42 to 166750.03)** | **43687.75 (34212.61 to 54141.37)** | **-4.89 (-5.71 to -4.05)** | **327 (274 to 397)** | **57 (46 to 70)** | **2085.07 (1752.12 to 2536.03)** | **603.05 (487.65 to 738.05)** | **-5.31 (-6.21 to -4.41)** |
| **Papua New Guinea** | **0.418098** | **Low SDI** | **1418216 (1244078 to 1603755)** | **2163380 (1804384 to 2543530)** | **218963.69 (192077.81 to 247609.67)** | **142204.85 (118607.08 to 167193.17)** | **-1.71 (-1.92 to -1.49)** | **23407 (21106 to 25942)** | **32853 (28403 to 37747)** | **3613.86 (3258.56 to 4005.24)** | **2159.55 (1867.02 to 2481.24)** | **-1.99 (-2.2 to -1.78)** |
| **Samoa** | **0.59234** | **Low-middle SDI** | **22640 (18171 to 27716)** | **16278 (12722 to 20334)** | **88815.26 (71284.64 to 108731.49)** | **55661.9 (43502.63 to 69531.16)** | **-1.26 (-1.45 to -1.08)** | **330 (270 to 395)** | **224 (179 to 275)** | **1294.54 (1060.3 to 1550.73)** | **765.81 (610.55 to 939.21)** | **-1.44 (-1.63 to -1.25)** |
| **Solomon Islands** | **0.429542** | **Low SDI** | **80608 (65438 to 98165)** | **44412 (37149 to 52127)** | **135123.95 (109693.68 to 164554.04)** | **46583.62 (38965.27 to 54675.94)** | **-3.55 (-3.8 to -3.29)** | **1229 (1052 to 1457)** | **635 (548 to 725)** | **2060.69 (1763.55 to 2442.69)** | **665.92 (575.11 to 760.35)** | **-3.75 (-4.01 to -3.49)** |
| **Tonga** | **0.629101** | **Middle SDI** | **11546 (9142 to 14158)** | **6857 (4995 to 9135)** | **75590.01 (59849.17 to 92692.22)** | **47590.17 (34671.57 to 63403.83)** | **-1.3 (-1.42 to -1.17)** | **165 (134 to 198)** | **90 (68 to 117)** | **1082.1 (878.96 to 1298.38)** | **627.08 (472.26 to 813.6)** | **-1.57 (-1.7 to -1.44)** |
| **Vanuatu** | **0.472796** | **Low-middle SDI** | **39371 (31709 to 47747)** | **27324 (22152 to 32582)** | **145602.14 (117266.01 to 176578.67)** | **64823.49 (52552.56 to 77296.24)** | **-2.68 (-3.14 to -2.22)** | **599 (502 to 717)** | **394 (334 to 460)** | **2215.42 (1857.1 to 2651.05)** | **935.22 (791.84 to 1091.37)** | **-2.85 (-3.32 to -2.37)** |
| **Central Asia** | **0.674963** | **NA** | **13569353 (11987381 to 15283989)** | **1812181 (1489983 to 2160246)** | **142467.3 (125857.87 to 160469.6)** | **18127.2 (14904.27 to 21608.89)** | **-6.68 (-7.05 to -6.3)** | **230193 (211932 to 251344)** | **25981 (22206 to 30146)** | **2416.84 (2225.11 to 2638.9)** | **259.89 (222.13 to 301.55)** | **-7.29 (-7.64 to -6.93)** |
| **Armenia** | **0.702497** | **Middle SDI** | **455614 (392115 to 533687)** | **35334 (27771 to 43980)** | **118981.81 (102399.31 to 139370.23)** | **18975.74 (14913.67 to 23618.57)** | **-6.02 (-7.07 to -4.94)** | **8332 (7489 to 9335)** | **498 (401 to 607)** | **2175.83 (1955.8 to 2437.78)** | **267.59 (215.58 to 325.81)** | **-6.96 (-7.94 to -5.98)** |
| **Azerbaijan** | **0.695411** | **Middle SDI** | **1010703 (875050 to 1185631)** | **202279 (159950 to 248720)** | **111833.52 (96823.7 to 131189.2)** | **28053.51 (22183.06 to 34494.34)** | **-4.78 (-5.46 to -4.1)** | **18962 (16921 to 21230)** | **2934 (2384 to 3545)** | **2098.16 (1872.26 to 2349.13)** | **406.85 (330.56 to 491.69)** | **-5.67 (-6.25 to -5.09)** |
| **Georgia** | **0.733124** | **High-middle SDI** | **480986 (414627 to 553783)** | **58791 (46170 to 72314)** | **102651.2 (88488.84 to 118187.24)** | **24164.65 (18976.9 to 29722.91)** | **-4.26 (-5.02 to -3.5)** | **7657 (6778 to 8602)** | **813 (651 to 984)** | **1634.18 (1446.56 to 1835.93)** | **334.12 (267.73 to 404.6)** | **-4.69 (-5.43 to -3.94)** |
| **Kazakhstan** | **0.718332** | **High-middle SDI** | **2178069 (1902278 to 2456192)** | **310917 (234730 to 398384)** | **115676.53 (101029.34 to 130447.54)** | **15957.22 (12047.08 to 20446.32)** | **-6.66 (-6.81 to -6.5)** | **37002 (33435 to 41279)** | **4244 (3261 to 5345)** | **1965.19 (1775.7 to 2192.32)** | **217.81 (167.35 to 274.32)** | **-7.36 (-7.53 to -7.2)** |
| **Kyrgyzstan** | **0.609181** | **Low-middle SDI** | **823602 (721051 to 953659)** | **155680 (130607 to 182437)** | **128138.58 (112183.38 to 148373.22)** | **19609.52 (16451.31 to 22979.9)** | **-5.75 (-6.09 to -5.42)** | **13417 (12168 to 14931)** | **2240 (1935 to 2556)** | **2087.47 (1893.1 to 2323.03)** | **282.21 (243.73 to 321.97)** | **-6.19 (-6.5 to -5.89)** |
| **Mongolia** | **0.618744** | **Low-middle SDI** | **421487 (340868 to 517009)** | **40464 (34204 to 47065)** | **124139.59 (100394.91 to 152273.27)** | **10357.03 (8754.55 to 12046.4)** | **-7.25 (-8.46 to -6.01)** | **6816 (5682 to 8231)** | **596 (522 to 684)** | **2007.62 (1673.37 to 2424.13)** | **152.53 (133.51 to 175.15)** | **-7.53 (-8.73 to -6.32)** |
| **Tajikistan** | **0.536613** | **Low-middle SDI** | **2341553 (2048733 to 2649176)** | **618635 (522477 to 700949)** | **247642.15 (216673.55 to 280176.33)** | **46219.12 (39034.98 to 52368.87)** | **-5.58 (-6.1 to -5.05)** | **43204 (38725 to 47730)** | **9270 (8104 to 10449)** | **4569.26 (4095.53 to 5047.86)** | **692.59 (605.49 to 780.67)** | **-6.41 (-6.91 to -5.91)** |
| **Turkmenistan** | **0.68304** | **Middle SDI** | **1216097 (1051184 to 1401885)** | **94942 (81661 to 110760)** | **207671.05 (179509.16 to 239397.75)** | **17591.13 (15130.4 to 20522.04)** | **-8.78 (-9.39 to -8.17)** | **20923 (18737 to 23513)** | **1358 (1204 to 1544)** | **3572.97 (3199.73 to 4015.22)** | **251.58 (223.06 to 286.08)** | **-9.37 (-9.95 to -8.79)** |
| **Uzbekistan** | **0.664965** | **Middle SDI** | **4641242 (3994232 to 5325069)** | **295138 (228558 to 368310)** | **137599.91 (118417.88 to 157873.48)** | **7695.42 (5959.4 to 9603.3)** | **-9.39 (-9.59 to -9.19)** | **73879 (67004 to 81563)** | **4028 (3204 to 4966)** | **2190.29 (1986.49 to 2418.12)** | **105.03 (83.53 to 129.49)** | **-9.9 (-10.13 to -9.66)** |
| **Central Europe** | **0.79578** | **NA** | **2067612 (1626373 to 2595319)** | **508648 (392608 to 638051)** | **22642.57 (17810.52 to 28421.52)** | **9106.3 (7028.84 to 11423)** | **-2.67 (-3.12 to -2.22)** | **35503 (29093 to 42940)** | **7795 (6306 to 9596)** | **388.8 (318.6 to 470.24)** | **139.56 (112.9 to 171.8)** | **-3.05 (-3.49 to -2.6)** |
| **Albania** | **0.706889** | **Middle SDI** | **140834 (115736 to 165705)** | **17449 (13310 to 22169)** | **34879.68 (28663.86 to 41039.28)** | **12236.12 (9333.44 to 15545.73)** | **-3.24 (-3.98 to -2.5)** | **2267 (1949 to 2625)** | **261 (203 to 330)** | **561.56 (482.79 to 650.05)** | **182.69 (142.29 to 231.6)** | **-3.45 (-4.23 to -2.67)** |
| **Bosnia and Herzegovina** | **0.722964** | **High-middle SDI** | **121362 (95944 to 147584)** | **25984 (20107 to 33530)** | **34517.27 (27287.92 to 41974.91)** | **17203.08 (13312.41 to 22199.33)** | **-2.03 (-2.67 to -1.39)** | **1894 (1576 to 2278)** | **397 (313 to 500)** | **538.68 (448.23 to 647.96)** | **262.75 (207.25 to 330.91)** | **-2.09 (-2.77 to -1.4)** |
| **Bulgaria** | **0.764641** | **High-middle SDI** | **112748 (90498 to 139388)** | **30888 (24520 to 37571)** | **21003.25 (16858.32 to 25965.79)** | **10204.84 (8101.06 to 12412.66)** | **-1.93 (-2.11 to -1.76)** | **1920 (1635 to 2242)** | **481 (399 to 578)** | **357.66 (304.6 to 417.62)** | **158.91 (131.98 to 190.88)** | **-2.28 (-2.45 to -2.11)** |
| **Croatia** | **0.799069** | **High-middle SDI** | **39453 (30080 to 51112)** | **17536 (13817 to 21615)** | **13095.33 (9984.32 to 16965.25)** | **9622.11 (7581.6 to 11860.33)** | **-0.51 (-0.65 to -0.37)** | **647 (520 to 808)** | **270 (220 to 332)** | **214.75 (172.64 to 268.15)** | **147.99 (120.65 to 182.43)** | **-0.64 (-0.8 to -0.48)** |
| **Czechia** | **0.82851** | **High SDI** | **74518 (53033 to 100136)** | **60962 (47282 to 76496)** | **11485.29 (8173.9 to 15433.67)** | **10846 (8412.26 to 13609.83)** | **0.02 (-0.38 to 0.42)** | **1121 (812 to 1509)** | **928 (739 to 1174)** | **172.76 (125.18 to 232.51)** | **165.04 (131.45 to 208.87)** | **0.03 (-0.39 to 0.45)** |
| **Hungary** | **0.791025** | **High-middle SDI** | **228196 (163113 to 298606)** | **67620 (51913 to 85258)** | **36854.66 (26343.4 to 48226.05)** | **14880.79 (11424.35 to 18762.45)** | **-3.47 (-3.84 to -3.1)** | **3656 (2636 to 4778)** | **1019 (807 to 1269)** | **590.49 (425.78 to 771.67)** | **224.2 (177.51 to 279.22)** | **-3.74 (-4.14 to -3.33)** |
| **North Macedonia** | **0.750955** | **High-middle SDI** | **140060 (122223 to 161898)** | **17612 (13521 to 22308)** | **82223.5 (71751.79 to 95043.65)** | **17521.22 (13450.86 to 22193.22)** | **-4.42 (-4.91 to -3.92)** | **2877 (2594 to 3196)** | **267 (212 to 331)** | **1688.79 (1522.8 to 1876.47)** | **265.9 (210.61 to 329.01)** | **-5.43 (-5.87 to -4.98)** |
| **Montenegro** | **0.796533** | **High-middle SDI** | **14746 (11030 to 19156)** | **4899 (3494 to 6552)** | **28106.3 (21024.31 to 36511.47)** | **13535.86 (9653.59 to 18104.17)** | **-1.26 (-1.97 to -0.53)** | **233 (180 to 303)** | **74 (53 to 101)** | **443.77 (342.79 to 577.67)** | **204.12 (147.27 to 279.05)** | **-1.3 (-2.07 to -0.52)** |
| **Poland** | **0.812073** | **NA** | **592132 (437629 to 795293)** | **60822 (46881 to 78382)** | **20286.73 (14993.4 to 27247.14)** | **3231.66 (2490.94 to 4164.67)** | **-5.63 (-6.51 to -4.74)** | **9976 (7468 to 13391)** | **979 (774 to 1248)** | **341.78 (255.87 to 458.77)** | **52 (41.15 to 66.31)** | **-5.75 (-6.57 to -4.92)** |
| **Romania** | **0.766321** | **High-middle SDI** | **360505 (307145 to 418690)** | **100190 (80385 to 122421)** | **20372.86 (17357.41 to 23661.03)** | **10682.89 (8571.23 to 13053.4)** | **-1.98 (-2.37 to -1.59)** | **7162 (6324 to 7971)** | **1532 (1271 to 1834)** | **404.71 (357.38 to 450.48)** | **163.4 (135.48 to 195.55)** | **-3.01 (-3.41 to -2.6)** |
| **Serbia** | **0.792213** | **High-middle SDI** | **149152 (109382 to 196194)** | **56599 (41540 to 73080)** | **21910.77 (16068.53 to 28821.43)** | **15352.68 (11267.95 to 19823.26)** | **0.26 (-0.76 to 1.29)** | **2260 (1728 to 2961)** | **853 (649 to 1099)** | **331.93 (253.9 to 435.04)** | **231.47 (176.02 to 298.02)** | **0.38 (-0.75 to 1.52)** |
| **Slovakia** | **0.808329** | **High-middle SDI** | **37011 (27627 to 48640)** | **20554 (15434 to 26799)** | **9045.42 (6751.88 to 11887.35)** | **7183.5 (5394.05 to 9365.87)** | **-0.29 (-0.48 to -0.1)** | **556 (432 to 711)** | **308 (241 to 393)** | **135.94 (105.64 to 173.87)** | **107.81 (84.06 to 137.21)** | **-0.3 (-0.49 to -0.11)** |
| **Slovenia** | **0.842633** | **High SDI** | **23829 (17518 to 31994)** | **20131 (14468 to 26747)** | **19374.28 (14242.87 to 26013.41)** | **20562.83 (14778.67 to 27320.58)** | **0.7 (0.35 to 1.05)** | **367 (277 to 487)** | **313 (232 to 411)** | **298.43 (225.37 to 395.57)** | **320.16 (236.87 to 420.16)** | **0.75 (0.4 to 1.1)** |
| **Eastern Europe** | **0.803414** | **NA** | **20106605 (16009831 to 25047216)** | **2635020 (1952430 to 3399970)** | **116613.14 (92852.91 to 145267.41)** | **26040.53 (19294.85 to 33600.13)** | **-4.33 (-4.88 to -3.78)** | **327481 (267796 to 401272)** | **38854 (29901 to 49232)** | **1899.31 (1553.15 to 2327.27)** | **383.97 (295.49 to 486.54)** | **-4.77 (-5.31 to -4.22)** |
| **Belarus** | **0.784114** | **High-middle SDI** | **707032 (585110 to 836742)** | **208447 (156872 to 270973)** | **87748.9 (72617.32 to 103847.09)** | **44601.06 (33565.58 to 57979.7)** | **-2.29 (-2.49 to -2.09)** | **11097 (9445 to 12868)** | **3066 (2334 to 3928)** | **1377.19 (1172.21 to 1597.03)** | **656.09 (499.34 to 840.52)** | **-2.54 (-2.73 to -2.35)** |
| **Estonia** | **0.845787** | **High SDI** | **86345 (64759 to 108971)** | **39608 (29276 to 52351)** | **71592.46 (53694.41 to 90353.27)** | **57257.92 (42321.16 to 75678.43)** | **-0.12 (-0.51 to 0.28)** | **1297 (1022 to 1618)** | **589 (451 to 765)** | **1074.99 (847.64 to 1341.7)** | **852 (652.51 to 1106.28)** | **-0.15 (-0.55 to 0.25)** |
| **Latvia** | **0.830715** | **High SDI** | **145590 (112737 to 184567)** | **35250 (26426 to 44986)** | **71847.76 (55635.33 to 91083.13)** | **37614.91 (28198.47 to 48003.2)** | **-1.78 (-2.1 to -1.46)** | **2191 (1735 to 2713)** | **520 (400 to 663)** | **1081.31 (855.98 to 1338.63)** | **554.99 (427.23 to 707.07)** | **-1.9 (-2.2 to -1.6)** |
| **Lithuania** | **0.857613** | **High SDI** | **214124 (162633 to 272100)** | **65792 (50306 to 83781)** | **74071.46 (56259.15 to 94126.99)** | **49996.27 (38228.02 to 63666.51)** | **-1.12 (-1.21 to -1.04)** | **3222 (2563 to 3987)** | **970 (756 to 1225)** | **1114.63 (886.53 to 1379.22)** | **737.38 (574.73 to 930.82)** | **-1.23 (-1.32 to -1.14)** |
| **Republic of Moldova** | **0.732393** | **High-middle SDI** | **432160 (366012 to 506987)** | **63723 (52775 to 76161)** | **100302.99 (84950.26 to 117669.98)** | **41318.85 (34220.14 to 49383.85)** | **-2.35 (-2.95 to -1.74)** | **8310 (7358 to 9392)** | **966 (821 to 1137)** | **1928.62 (1707.74 to 2179.74)** | **626.11 (532.49 to 737.41)** | **-3.27 (-3.76 to -2.79)** |
| **Russian Federation** | **0.809111** | **NA** | **15093172 (12026300 to 18700579)** | **1857957 (1378362 to 2398070)** | **129841.84 (103458.5 to 160875.24)** | **24412.58 (18110.95 to 31509.38)** | **-4.69 (-5.34 to -4.02)** | **245460 (200824 to 300133)** | **27593 (21390 to 34892)** | **2111.61 (1727.62 to 2581.95)** | **362.55 (281.05 to 458.46)** | **-5.12 (-5.77 to -4.47)** |
| **Ukraine** | **0.761046** | **NA** | **3428183 (2635480 to 4343333)** | **364242 (254068 to 488184)** | **90958.56 (69926.1 to 115239.84)** | **22876.65 (15957.04 to 30660.96)** | **-4.75 (-5.01 to -4.48)** | **55906 (43621 to 70207)** | **5149 (3775 to 6805)** | **1483.32 (1157.38 to 1862.77)** | **323.41 (237.09 to 427.37)** | **-5.24 (-5.51 to -4.96)** |
| **High-income Asia Pacific** | **0.877157** | **NA** | **6882911 (4866472 to 9311078)** | **5459346 (3902131 to 7237515)** | **67374.35 (47636.16 to 91142.81)** | **84612.72 (60477.92 to 112172.01)** | **1.38 (0.93 to 1.83)** | **106258 (78572 to 141992)** | **81519 (61484 to 105829)** | **1040.13 (769.11 to 1389.91)** | **1263.44 (952.92 to 1640.22)** | **1.28 (0.83 to 1.73)** |
| **Brunei Darussalam** | **0.810289** | **High-middle SDI** | **5026 (3448 to 7001)** | **2826 (1913 to 3922)** | **14572.69 (9998.76 to 20302.24)** | **9146.91 (6190.71 to 12695.57)** | **-1.51 (-2 to -1.01)** | **71 (49 to 98)** | **39 (27 to 53)** | **206.45 (141.58 to 284.17)** | **126.97 (87.67 to 171.76)** | **-1.59 (-2.1 to -1.07)** |
| **Japan** | **0.87146** | **NA** | **6154729 (4345691 to 8369527)** | **5189487 (3715425 to 6871639)** | **92436.97 (65267.3 to 125700.7)** | **113173.26 (81026.65 to 149857.91)** | **1.26 (0.85 to 1.67)** | **95676 (70495 to 129006)** | **77577 (58549 to 100850)** | **1436.95 (1058.75 to 1937.53)** | **1691.82 (1276.85 to 2199.36)** | **1.14 (0.73 to 1.55)** |
| **Republic of Korea** | **0.887196** | **High SDI** | **701795 (498107 to 920616)** | **251140 (172362 to 350478)** | **21144.17 (15007.32 to 27736.98)** | **16203.64 (11120.86 to 22612.98)** | **-0.52 (-1.05 to 0.01)** | **10187 (7601 to 13133)** | **3662 (2611 to 4994)** | **306.93 (229.01 to 395.68)** | **236.29 (168.49 to 322.24)** | **-0.48 (-1.02 to 0.07)** |
| **Singapore** | **0.856235** | **High SDI** | **21362 (16066 to 26975)** | **15893 (11646 to 21442)** | **10469.12 (7873.97 to 13220.36)** | **5558.48 (4073.1 to 7499.14)** | **-1.56 (-1.83 to -1.28)** | **324 (253 to 404)** | **240 (179 to 322)** | **158.65 (123.87 to 198.04)** | **84.09 (62.46 to 112.57)** | **-1.59 (-1.85 to -1.33)** |
| **Australasia** | **0.845644** | **NA** | **507880 (365126 to 671707)** | **203334 (151079 to 266500)** | **32930.58 (23674.52 to 43552.97)** | **11196.47 (8319.09 to 14674.7)** | **-1.64 (-2.22 to -1.05)** | **7685 (5794 to 10336)** | **3122 (2440 to 4065)** | **498.27 (375.67 to 670.19)** | **171.89 (134.37 to 223.82)** | **-1.53 (-2.14 to -0.91)** |
| **Australia** | **0.844269** | **High SDI** | **380445 (276969 to 503872)** | **120423 (87096 to 162004)** | **30131.15 (21935.93 to 39906.6)** | **8009.77 (5793.08 to 10775.43)** | **-1.86 (-2.8 to -0.91)** | **5764 (4239 to 7724)** | **1794 (1344 to 2387)** | **456.49 (335.74 to 611.71)** | **119.29 (89.38 to 158.78)** | **-1.85 (-2.79 to -0.91)** |
| **New Zealand** | **0.850145** | **NA** | **127436 (88651 to 172549)** | **82911 (62107 to 106868)** | **45570.22 (31700.91 to 61702.58)** | **26522.98 (19867.78 to 34186.97)** | **-0.97 (-1.83 to -0.1)** | **1921 (1397 to 2634)** | **1328 (1045 to 1695)** | **686.9 (499.61 to 941.9)** | **424.88 (334.23 to 542.24)** | **-0.71 (-1.6 to 0.19)** |
| **Western Europe** | **0.848729** | **NA** | **19407606 (13551032 to 26345487)** | **15502480 (11131295 to 20392869)** | **84540.02 (59028.64 to 114761.61)** | **73024.26 (52433.84 to 96060.38)** | **-0.11 (-0.76 to 0.55)** | **290323 (210869 to 396536)** | **237220 (175147 to 312801)** | **1264.65 (918.55 to 1727.32)** | **1117.42 (825.03 to 1473.44)** | **-0.06 (-0.71 to 0.6)** |
| **Andorra** | **0.869895** | **High SDI** | **2641 (1855 to 3611)** | **2473 (1683 to 3434)** | **97552.25 (68526.03 to 133409.29)** | **97947.07 (66646.84 to 136040.6)** | **0.38 (-0.18 to 0.94)** | **40 (29 to 55)** | **39 (27 to 54)** | **1463.13 (1060.48 to 2045.74)** | **1529.87 (1058.21 to 2133.44)** | **0.51 (-0.05 to 1.07)** |
| **Austria** | **0.854558** | **High SDI** | **766265 (570339 to 984742)** | **322913 (245585 to 414446)** | **171412.76 (127584.34 to 220286.07)** | **74728.02 (56832.85 to 95910.48)** | **-3.11 (-4.06 to -2.15)** | **12548 (9427 to 16131)** | **4996 (3921 to 6314)** | **2807.06 (2108.85 to 3608.39)** | **1156.19 (907.37 to 1461.11)** | **-3.41 (-4.4 to -2.4)** |
| **Belgium** | **0.853674** | **High SDI** | **457364 (314688 to 629688)** | **329555 (238415 to 438624)** | **76674.27 (52755.5 to 105563.48)** | **55674.11 (40277.07 to 74099.82)** | **-0.91 (-1.62 to -0.19)** | **6795 (4794 to 9367)** | **4929 (3592 to 6672)** | **1139.21 (803.74 to 1570.34)** | **832.76 (606.86 to 1127.14)** | **-0.91 (-1.61 to -0.2)** |
| **Cyprus** | **0.835649** | **High SDI** | **56186 (38883 to 77132)** | **74835 (50992 to 103176)** | **87927.79 (60849.91 to 120707.77)** | **99723.43 (67951.36 to 137491.45)** | **0.98 (0.36 to 1.61)** | **832 (583 to 1160)** | **1156 (802 to 1603)** | **1302.32 (912.18 to 1814.78)** | **1539.9 (1068.71 to 2135.71)** | **1.1 (0.48 to 1.72)** |
| **Denmark** | **0.897314** | **High SDI** | **259885 (182320 to 350441)** | **323131 (236911 to 426151)** | **89737.87 (62954.79 to 121006.69)** | **104003.06 (76252.15 to 137161)** | **1.11 (0.5 to 1.72)** | **3820 (2780 to 5219)** | **4862 (3619 to 6396)** | **1318.98 (959.95 to 1802.03)** | **1564.84 (1164.88 to 2058.56)** | **1.18 (0.58 to 1.78)** |
| **Finland** | **0.860244** | **High SDI** | **294456 (206834 to 397978)** | **151540 (103548 to 207701)** | **94172.75 (66149.43 to 127281.19)** | **62413.69 (42647.5 to 85544.47)** | **-1.17 (-1.98 to -0.35)** | **4351 (3105 to 5886)** | **2287 (1609 to 3173)** | **1391.47 (992.93 to 1882.36)** | **941.92 (662.53 to 1306.97)** | **-1.13 (-1.94 to -0.3)** |
| **France** | **0.837816** | **High SDI** | **3731280 (2637254 to 4960241)** | **4679330 (3223729 to 6332680)** | **95935.04 (67806.52 to 127532.89)** | **132411.89 (91222.48 to 179197.04)** | **1.35 (0.93 to 1.78)** | **55167 (40432 to 72786)** | **71895 (50649 to 97422)** | **1418.4 (1039.55 to 1871.39)** | **2034.43 (1433.21 to 2756.78)** | **1.47 (1.06 to 1.87)** |
| **Germany** | **0.903516** | **High SDI** | **4128988 (2828659 to 5778586)** | **2246689 (1732291 to 2859620)** | **92181.34 (63150.97 to 129009.28)** | **55563.51 (42841.79 to 70722.08)** | **-1.1 (-2.26 to 0.08)** | **61891 (42662 to 86558)** | **34506 (26986 to 43682)** | **1381.74 (952.44 to 1932.44)** | **853.38 (667.4 to 1080.31)** | **-1.07 (-2.3 to 0.17)** |
| **Greece** | **0.791882** | **High-middle SDI** | **554063 (384296 to 761954)** | **320239 (216195 to 438160)** | **99836.34 (69246.06 to 137296.16)** | **75775.9 (51156.69 to 103678.94)** | **-0.16 (-0.85 to 0.53)** | **8409 (5959 to 11703)** | **4883 (3319 to 6753)** | **1515.27 (1073.79 to 2108.8)** | **1155.53 (785.46 to 1597.81)** | **-0.16 (-0.84 to 0.53)** |
| **Iceland** | **0.874629** | **High SDI** | **20106 (13801 to 27724)** | **21269 (14772 to 28613)** | **95168.21 (65324.87 to 131229.22)** | **96799.62 (67231.48 to 130221.5)** | **0.47 (-0.11 to 1.06)** | **300 (212 to 417)** | **327 (235 to 450)** | **1418.99 (1005.17 to 1973.18)** | **1485.98 (1070.09 to 2047.75)** | **0.55 (-0.03 to 1.12)** |
| **Ireland** | **0.87399** | **High SDI** | **249362 (167076 to 351205)** | **272518 (190582 to 375216)** | **86290.6 (57816 to 121533.14)** | **91326.6 (63868.05 to 125742.85)** | **1.94 (1.02 to 2.87)** | **3749 (2543 to 5223)** | **4160 (2934 to 5788)** | **1297.22 (880.09 to 1807.28)** | **1394.24 (983.41 to 1939.72)** | **2.04 (1.12 to 2.97)** |
| **Israel** | **0.809091** | **High-middle SDI** | **385570 (267935 to 530745)** | **485285 (343501 to 660213)** | **74670.44 (51888.96 to 102785.42)** | **52847.43 (37407.24 to 71897.08)** | **-0.69 (-1.53 to 0.15)** | **5686 (4034 to 7979)** | **7197 (5154 to 9732)** | **1101.19 (781.28 to 1545.23)** | **783.77 (561.3 to 1059.84)** | **-0.69 (-1.52 to 0.14)** |
| **Italy** | **0.805537** | **NA** | **1396970 (959991 to 1934435)** | **649551 (475988 to 854195)** | **50877.9 (34963.02 to 70452.45)** | **29931.28 (21933.51 to 39361.28)** | **-1.26 (-1.96 to -0.56)** | **20858 (14835 to 29121)** | **10331 (7753 to 13754)** | **759.67 (540.3 to 1060.59)** | **476.08 (357.26 to 633.77)** | **-1 (-1.74 to -0.26)** |
| **Luxembourg** | **0.884636** | **High SDI** | **18580 (13269 to 24654)** | **20186 (14357 to 27004)** | **81147.81 (57952.24 to 107672.73)** | **61050.01 (43421.2 to 81670.01)** | **-0.76 (-1.51 to -0.01)** | **271 (199 to 360)** | **302 (224 to 406)** | **1185.65 (867.47 to 1574.34)** | **913.47 (676.67 to 1227.23)** | **-0.69 (-1.44 to 0.06)** |
| **Malta** | **0.801854** | **High-middle SDI** | **24734 (16407 to 34407)** | **19144 (13071 to 25858)** | **87571.71 (58092.13 to 121820.69)** | **87007.51 (59407.23 to 117524.58)** | **0.4 (-0.17 to 0.97)** | **372 (252 to 521)** | **293 (204 to 398)** | **1316.58 (891.19 to 1844.35)** | **1330.48 (925.92 to 1808.08)** | **0.42 (-0.13 to 0.97)** |
| **Netherlands** | **0.888376** | **High SDI** | **1018518 (701280 to 1413343)** | **1431436 (963981 to 1902830)** | **108779.32 (74897.84 to 150947.26)** | **166208.95 (111931.07 to 220944.04)** | **1.92 (1.27 to 2.58)** | **15541 (10658 to 21882)** | **22583 (15504 to 30654)** | **1659.77 (1138.31 to 2337.06)** | **2622.19 (1800.27 to 3559.28)** | **2.04 (1.39 to 2.69)** |
| **Norway** | **0.916632** | **NA** | **154623 (105350 to 214097)** | **133058 (94744 to 178148)** | **56052.54 (38190.7 to 77612.71)** | **47331.95 (33702.8 to 63371.39)** | **-1.1 (-1.36 to -0.84)** | **2276 (1628 to 3172)** | **2037 (1489 to 2777)** | **825.22 (590.28 to 1150.06)** | **724.7 (529.82 to 987.91)** | **-0.9 (-1.14 to -0.67)** |
| **Portugal** | **0.745395** | **High-middle SDI** | **483515 (360357 to 625980)** | **223709 (166135 to 285415)** | **83592.61 (62300.37 to 108222.7)** | **52597.11 (39060.66 to 67104.91)** | **-0.69 (-1 to -0.37)** | **7564 (5915 to 9376)** | **3352 (2566 to 4308)** | **1307.72 (1022.54 to 1621.01)** | **788.2 (603.25 to 1012.88)** | **-0.7 (-1.03 to -0.37)** |
| **Spain** | **0.769483** | **High-middle SDI** | **1827491 (1284033 to 2445447)** | **1517022 (1128437 to 1965172)** | **87744.31 (61650.96 to 117414.57)** | **82390.24 (61286.01 to 106729.47)** | **0.22 (-0.16 to 0.6)** | **26867 (19338 to 36766)** | **23272 (17740 to 30590)** | **1289.98 (928.49 to 1765.25)** | **1263.92 (963.45 to 1661.35)** | **0.36 (0 to 0.73)** |
| **Sweden** | **0.887384** | **NA** | **303400 (210065 to 414196)** | **294100 (209531 to 393658)** | **53884.31 (37307.85 to 73561.93)** | **50429.33 (35928.3 to 67500.53)** | **0.23 (0.11 to 0.35)** | **4505 (3208 to 6361)** | **4447 (3266 to 6170)** | **800.11 (569.79 to 1129.76)** | **762.55 (559.98 to 1057.96)** | **0.27 (0.15 to 0.39)** |
| **Switzerland** | **0.933532** | **High SDI** | **523441 (387882 to 682819)** | **580442 (432379 to 754524)** | **131412.26 (97379.68 to 171424.84)** | **131322.85 (97824.22 to 170708.23)** | **0.33 (-0.15 to 0.8)** | **7809 (5926 to 10070)** | **8797 (6711 to 11464)** | **1960.46 (1487.84 to 2528.2)** | **1990.38 (1518.34 to 2593.6)** | **0.36 (-0.11 to 0.83)** |
| **United Kingdom** | **0.858445** | **NA** | **2732096 (1867083 to 3815484)** | **1387934 (947777 to 1912973)** | **71121.31 (48603.49 to 99323.85)** | **37977.56 (25933.7 to 52344.02)** | **-1.44 (-2.34 to -0.53)** | **40401 (28318 to 56969)** | **20320 (14551 to 28108)** | **1051.7 (737.17 to 1483.01)** | **556.02 (398.16 to 769.1)** | **-1.48 (-2.37 to -0.58)** |
| **Southern Latin America** | **0.74303** | **NA** | **5801009 (4745880 to 7021947)** | **1061441 (787147 to 1382548)** | **112707.95 (92207.81 to 136429.58)** | **24808.92 (18397.9 to 32314.12)** | **-3.97 (-5.08 to -2.85)** | **87174 (73826 to 102068)** | **15280 (11868 to 19168)** | **1693.7 (1434.36 to 1983.09)** | **357.15 (277.4 to 448.02)** | **-4.03 (-5.16 to -2.88)** |
| **Argentina** | **0.733528** | **High-middle SDI** | **4537589 (3689916 to 5485724)** | **750891 (554580 to 995160)** | **132029.17 (107364.64 to 159616.83)** | **25060.91 (18509.06 to 33213.37)** | **-4.09 (-5.27 to -2.9)** | **68184 (58038 to 80221)** | **10693 (8277 to 13600)** | **1983.93 (1688.71 to 2334.18)** | **356.89 (276.25 to 453.91)** | **-4.16 (-5.37 to -2.94)** |
| **Chile** | **0.770149** | **High-middle SDI** | **1006863 (769926 to 1264173)** | **270445 (200798 to 342252)** | **70064.66 (53576.9 to 87970.09)** | **24857.92 (18456.38 to 31458.11)** | **-3.82 (-4.86 to -2.76)** | **14949 (12053 to 18219)** | **4015 (3100 to 5095)** | **1040.26 (838.75 to 1267.81)** | **369.07 (284.92 to 468.28)** | **-3.77 (-4.84 to -2.69)** |
| **Uruguay** | **0.721713** | **High-middle SDI** | **256281 (210256 to 310895)** | **40046 (30268 to 52491)** | **93931.76 (77062.65 to 113948.57)** | **20642.18 (15601.74 to 27056.83)** | **-4.98 (-5.95 to -4.01)** | **4037 (3408 to 4750)** | **571 (439 to 729)** | **1479.49 (1249.16 to 1740.8)** | **294.2 (226.12 to 375.53)** | **-5.22 (-6.16 to -4.28)** |
| **High-income North America** | **0.864217** | **NA** | **7545115 (5181665 to 10576161)** | **642275 (499750 to 817522)** | **34799.33 (23898.7 to 48779.02)** | **3133.27 (2437.97 to 3988.19)** | **-7.84 (-8.85 to -6.82)** | **121328 (84137 to 170863)** | **10735 (8578 to 13425)** | **559.59 (388.05 to 788.05)** | **52.37 (41.84 to 65.49)** | **-7.9 (-8.89 to -6.9)** |
| **Canada** | **0.873182** | **High SDI** | **904043 (630188 to 1232837)** | **203480 (145433 to 269563)** | **46962.03 (32736.15 to 64041.77)** | **10708.97 (7654.01 to 14186.85)** | **-4.67 (-5.6 to -3.73)** | **14406 (10020 to 20207)** | **3117 (2314 to 4196)** | **748.36 (520.5 to 1049.71)** | **164.04 (121.78 to 220.81)** | **-4.81 (-5.74 to -3.87)** |
| **United States of America** | **0.863244** | **NA** | **6640007 (4533531 to 9383973)** | **438577 (346080 to 557539)** | **33619.05 (22953.74 to 47512.04)** | **2358.69 (1861.23 to 2998.47)** | **-9.68 (-11.2 to -8.14)** | **106906 (73618 to 152070)** | **7615 (6115 to 9582)** | **541.28 (372.74 to 769.95)** | **40.95 (32.89 to 51.53)** | **-9.56 (-11.02 to -8.07)** |
| **Caribbean** | **0.642315** | **NA** | **4933546 (4205493 to 5670210)** | **1483433 (1211874 to 1786443)** | **119415.3 (101792.96 to 137246.08)** | **38349.64 (31329.31 to 46183.04)** | **-3.32 (-3.81 to -2.82)** | **74428 (66603 to 82313)** | **21177 (18054 to 24894)** | **1801.51 (1612.1 to 1992.35)** | **547.47 (466.73 to 643.56)** | **-3.5 (-3.99 to -3.01)** |
| **Antigua and Barbuda** | **0.74985** | **High-middle SDI** | **3039 (2433 to 3778)** | **1512 (1119 to 2002)** | **50148.07 (40149.02 to 62328.07)** | **28683.4 (21221.75 to 37978.01)** | **-1.86 (-2.08 to -1.65)** | **42 (34 to 51)** | **20 (15 to 26)** | **695.44 (566.82 to 840.71)** | **377.6 (286.58 to 489.21)** | **-2.04 (-2.25 to -1.83)** |
| **Bahamas** | **0.805144** | **High-middle SDI** | **10012 (7774 to 12431)** | **3649 (2491 to 5067)** | **39259.97 (30483.85 to 48744.94)** | **17332.45 (11830.17 to 24065.63)** | **-2.11 (-2.54 to -1.68)** | **137 (109 to 167)** | **47 (34 to 64)** | **537.25 (427.29 to 655.72)** | **222.67 (159.25 to 304.75)** | **-2.31 (-2.72 to -1.9)** |
| **Barbados** | **0.747065** | **High-middle SDI** | **9768 (7507 to 12446)** | **4822 (3267 to 6759)** | **49883.02 (38336.5 to 63557.51)** | **35408.7 (23993.68 to 49635.56)** | **-0.88 (-1.21 to -0.54)** | **134 (106 to 165)** | **63 (44 to 86)** | **683.71 (542.36 to 842.15)** | **459.87 (324.82 to 631.45)** | **-1.02 (-1.36 to -0.68)** |
| **Belize** | **0.610552** | **Low-middle SDI** | **26049 (21785 to 29968)** | **10108 (8042 to 12334)** | **88174.65 (73741.82 to 101443.57)** | **26506.75 (21089.35 to 32346.35)** | **-3.14 (-3.6 to -2.67)** | **377 (332 to 423)** | **137 (114 to 164)** | **1277.31 (1123.82 to 1432.61)** | **358.08 (298.65 to 431.25)** | **-3.35 (-3.82 to -2.87)** |
| **Cuba** | **0.669332** | **Middle SDI** | **387435 (288593 to 496034)** | **203042 (135825 to 285110)** | **43285.78 (32242.75 to 55418.89)** | **37292 (24946.52 to 52365.05)** | **0.19 (-0.17 to 0.55)** | **5648 (4474 to 7075)** | **2701 (1843 to 3788)** | **631.03 (499.85 to 790.45)** | **496.16 (338.47 to 695.77)** | **-0.01 (-0.38 to 0.35)** |
| **Dominica** | **0.747382** | **High-middle SDI** | **3290 (2609 to 3979)** | **757 (570 to 967)** | **37984.32 (30129.35 to 45939.68)** | **21758.19 (16396.04 to 27805.75)** | **-1.78 (-2.03 to -1.53)** | **45 (37 to 55)** | **10 (8 to 13)** | **525.15 (432.75 to 631.78)** | **289.96 (226.53 to 368.84)** | **-1.9 (-2.12 to -1.68)** |
| **Dominican Republic** | **0.619171** | **Middle SDI** | **1919373 (1658930 to 2162635)** | **287422 (232207 to 346524)** | **192737.41 (166584.52 to 217164.98)** | **27820.65 (22476.18 to 33541.43)** | **-5.32 (-6.15 to -4.48)** | **29206 (26331 to 32119)** | **4107 (3403 to 4913)** | **2932.73 (2644.09 to 3225.29)** | **397.51 (329.43 to 475.59)** | **-5.53 (-6.38 to -4.69)** |
| **Grenada** | **0.669351** | **Middle SDI** | **5721 (4502 to 7159)** | **2200 (1475 to 3144)** | **47637.45 (37489.35 to 59613.51)** | **32030.17 (21480.74 to 45780.7)** | **-1.28 (-1.68 to -0.88)** | **78 (65 to 96)** | **28 (19 to 38)** | **652.43 (537.21 to 797.2)** | **404.63 (280.86 to 560.14)** | **-1.54 (-1.91 to -1.17)** |
| **Guyana** | **0.650902** | **Middle SDI** | **69577 (60215 to 79475)** | **10876 (9284 to 12739)** | **61680.83 (53381.4 to 70455.64)** | **14596.48 (12459.58 to 17096.42)** | **-4.74 (-5.28 to -4.2)** | **995 (888 to 1114)** | **150 (132 to 171)** | **882.48 (786.86 to 987.88)** | **201.56 (177.44 to 229.59)** | **-4.9 (-5.43 to -4.38)** |
| **Haiti** | **0.448751** | **Low SDI** | **1674415 (1393956 to 1965942)** | **793143 (670466 to 938348)** | **157859.17 (131418.26 to 185343.54)** | **50523.6 (42709.01 to 59773.21)** | **-3.61 (-4.04 to -3.17)** | **25881 (22428 to 29639)** | **11637 (10183 to 13348)** | **2440.03 (2114.45 to 2794.32)** | **741.31 (648.69 to 850.25)** | **-3.8 (-4.22 to -3.37)** |
| **Jamaica** | **0.683064** | **Middle SDI** | **348416 (297448 to 399923)** | **38562 (28913 to 49365)** | **124876.27 (106608.73 to 143336.89)** | **22527.02 (16890.41 to 28837.56)** | **-4.96 (-5.32 to -4.6)** | **5046 (4516 to 5673)** | **513 (397 to 646)** | **1808.55 (1618.58 to 2033.35)** | **299.9 (232.04 to 377.46)** | **-5.25 (-5.63 to -4.87)** |
| **Saint Lucia** | **0.672602** | **Middle SDI** | **10241 (8453 to 12266)** | **2971 (2224 to 3895)** | **57996.57 (47872.21 to 69460.58)** | **33635.15 (25178.39 to 44095.71)** | **-1.52 (-1.78 to -1.27)** | **144 (123 to 168)** | **39 (31 to 50)** | **815.84 (697.01 to 950.42)** | **444.63 (346.98 to 566.74)** | **-1.72 (-1.97 to -1.47)** |
| **Saint Vincent and the Grenadines** | **0.640887** | **Middle SDI** | **9778 (8053 to 11548)** | **1694 (1314 to 2167)** | **76524.52 (63024.74 to 90377.53)** | **23541.75 (18266.23 to 30116.81)** | **-3.72 (-3.87 to -3.58)** | **141 (121 to 163)** | **23 (18 to 28)** | **1103.91 (944.54 to 1274.37)** | **314.45 (248.27 to 389.23)** | **-3.98 (-4.13 to -3.82)** |
| **Suriname** | **0.641163** | **Middle SDI** | **42085 (35762 to 48453)** | **10517 (8910 to 12518)** | **96076.53 (81641.58 to 110614.59)** | **23613.99 (20005.32 to 28107.7)** | **-4.62 (-5.04 to -4.2)** | **619 (545 to 703)** | **150 (130 to 174)** | **1412.51 (1244.2 to 1604.07)** | **336.22 (291.4 to 391.33)** | **-4.75 (-5.15 to -4.35)** |
| **Trinidad and Tobago** | **0.769401** | **High-middle SDI** | **58004 (47986 to 69445)** | **16893 (12725 to 21698)** | **43508.13 (35994 to 52089.8)** | **20981.46 (15804.96 to 26950.11)** | **-1.9 (-2.27 to -1.53)** | **809 (691 to 944)** | **223 (173 to 278)** | **607.06 (518.6 to 708.4)** | **277.3 (215.37 to 345.11)** | **-2.07 (-2.43 to -1.71)** |
| **Andean Latin America** | **0.654008** | **NA** | **15756871 (14494989 to 16953387)** | **1854614 (1468523 to 2287471)** | **298341.14 (274448.62 to 320996.02)** | **30127.88 (23855.9 to 37159.56)** | **-7.4 (-8.14 to -6.64)** | **285495 (266165 to 304140)** | **26405 (21766 to 31326)** | **5405.56 (5039.58 to 5758.6)** | **428.94 (353.58 to 508.88)** | **-7.97 (-8.7 to -7.23)** |
| **Bolivia (Plurinational State of)** | **0.604497** | **Low-middle SDI** | **2537612 (2208755 to 2777342)** | **290962 (228645 to 357615)** | **251261.51 (218699.7 to 274998.29)** | **24364.88 (19146.5 to 29946.32)** | **-7.81 (-8.85 to -6.76)** | **44239 (38815 to 49126)** | **4216 (3464 to 5059)** | **4380.3 (3843.26 to 4864.25)** | **353.08 (290.08 to 423.62)** | **-8.3 (-9.38 to -7.21)** |
| **Ecuador** | **0.665675** | **Middle SDI** | **4443239 (4072020 to 4825484)** | **355704 (279599 to 440572)** | **330213.18 (302624.91 to 358620.9)** | **21413.44 (16831.93 to 26522.5)** | **-9.47 (-10.37 to -8.56)** | **79265 (73318 to 85365)** | **4900 (4018 to 5953)** | **5890.79 (5448.82 to 6344.19)** | **294.97 (241.9 to 358.36)** | **-10.21 (-11.08 to -9.33)** |
| **Peru** | **0.662036** | **Middle SDI** | **8776020 (8100504 to 9503943)** | **1207948 (937652 to 1496973)** | **299934.48 (276847.65 to 324812.42)** | **36598.99 (28409.43 to 45355.99)** | **-6.4 (-7.08 to -5.72)** | **161991 (149389 to 173581)** | **17289 (14084 to 20378)** | **5536.31 (5105.62 to 5932.4)** | **523.82 (426.71 to 617.42)** | **-6.96 (-7.63 to -6.28)** |
| **Central Latin America** | **0.641931** | **NA** | **32395748 (26553639 to 38804541)** | **6445992 (5124297 to 7889307)** | **140741.46 (115360.75 to 168584.09)** | **32084.93 (25506.19 to 39269.03)** | **-5 (-5.25 to -4.75)** | **508371 (432749 to 595721)** | **95392 (79467 to 113631)** | **2208.59 (1880.05 to 2588.08)** | **474.81 (395.55 to 565.6)** | **-5.18 (-5.41 to -4.94)** |
| **Colombia** | **0.65664** | **Middle SDI** | **5490207 (4605212 to 6299704)** | **1339850 (963528 to 1692563)** | **130503.8 (109467.2 to 149745.77)** | **38917.81 (27987.03 to 49162.86)** | **-4.52 (-5.02 to -4.03)** | **85925 (76014 to 97157)** | **18976 (14378 to 23764)** | **2042.45 (1806.88 to 2309.45)** | **551.19 (417.62 to 690.25)** | **-4.88 (-5.39 to -4.38)** |
| **Costa Rica** | **0.70437** | **Middle SDI** | **339510 (269184 to 409505)** | **161573 (116084 to 219782)** | **83629.35 (66306.45 to 100870.78)** | **52407.44 (37652.69 to 71287.98)** | **-1.28 (-1.35 to -1.21)** | **5314 (4449 to 6314)** | **2245 (1658 to 2927)** | **1309.01 (1095.8 to 1555.39)** | **728.07 (537.91 to 949.52)** | **-1.63 (-1.71 to -1.55)** |
| **El Salvador** | **0.56557** | **Low-middle SDI** | **1234019 (1038714 to 1437297)** | **166866 (123300 to 219217)** | **160245.02 (134883.5 to 186641.93)** | **27777.32 (20525.1 to 36491.82)** | **-5.9 (-6.43 to -5.37)** | **19829 (17423 to 22394)** | **2271 (1762 to 2916)** | **2574.87 (2262.51 to 2908.06)** | **377.97 (293.26 to 485.46)** | **-6.36 (-6.9 to -5.81)** |
| **Guatemala** | **0.540099** | **Low-middle SDI** | **2616391 (2231639 to 3075069)** | **734973 (615423 to 873947)** | **170556.9 (145475.74 to 200457.16)** | **47135.82 (39468.75 to 56048.62)** | **-4.08 (-4.43 to -3.72)** | **42580 (37628 to 47916)** | **11717 (10297 to 13321)** | **2775.68 (2452.9 to 3123.54)** | **751.47 (660.4 to 854.31)** | **-4.13 (-4.51 to -3.76)** |
| **Honduras** | **0.513586** | **Low-middle SDI** | **967409 (787711 to 1170437)** | **350657 (284254 to 426383)** | **117545.91 (95711.49 to 142215.04)** | **32008.19 (25946.89 to 38920.51)** | **-4.44 (-4.93 to -3.96)** | **15036 (12821 to 17464)** | **4877 (4092 to 5780)** | **1826.97 (1557.87 to 2121.94)** | **445.21 (373.48 to 527.56)** | **-4.74 (-5.23 to -4.25)** |
| **Mexico** | **0.664969** | **NA** | **17523051 (13454313 to 22269220)** | **2176728 (1668163 to 2747816)** | **148525.08 (114038.53 to 188753.53)** | **22038.08 (16889.16 to 27820.01)** | **-6.07 (-6.32 to -5.82)** | **269910 (211108 to 343440)** | **33327 (26631 to 41748)** | **2287.75 (1789.34 to 2911)** | **337.41 (269.62 to 422.68)** | **-6.06 (-6.28 to -5.83)** |
| **Nicaragua** | **0.523647** | **Low-middle SDI** | **1343147 (1124624 to 1566880)** | **185426 (144482 to 225682)** | **202430.99 (169496.45 to 236150.65)** | **28503.66 (22209.7 to 34691.73)** | **-7.65 (-8.27 to -7.02)** | **23176 (20143 to 26732)** | **2591 (2124 to 3152)** | **3492.95 (3035.83 to 4028.94)** | **398.33 (326.57 to 484.57)** | **-8.36 (-9.01 to -7.71)** |
| **Panama** | **0.70666** | **Middle SDI** | **469374 (384576 to 566563)** | **300345 (241707 to 362083)** | **164371.84 (134676.12 to 198406.73)** | **80904.68 (65109.31 to 97535.26)** | **-2.61 (-3.09 to -2.13)** | **7567 (6452 to 8921)** | **4660 (3884 to 5611)** | **2650.05 (2259.33 to 3124.06)** | **1255.31 (1046.35 to 1511.5)** | **-2.72 (-3.22 to -2.22)** |
| **Venezuela (Bolivarian Republic of)** | **0.5966** | **Low-middle SDI** | **2412641 (2007384 to 2839934)** | **1029575 (826623 to 1254094)** | **95331.54 (79318.49 to 112215.34)** | **47121.58 (37832.87 to 57397.37)** | **-3.13 (-3.41 to -2.84)** | **39034 (34727 to 44311)** | **14728 (12519 to 17382)** | **1542.36 (1372.17 to 1750.87)** | **674.06 (572.97 to 795.53)** | **-3.56 (-3.85 to -3.27)** |
| **Tropical Latin America** | **0.648942** | **NA** | **28730853 (23019747 to 34934913)** | **5813628 (4434811 to 7392157)** | **168221.48 (134782.5 to 204546.76)** | **33785.22 (25772.38 to 42958.65)** | **-6.11 (-6.5 to -5.72)** | **480762 (398954 to 572411)** | **85298 (66820 to 106347)** | **2814.9 (2335.91 to 3351.51)** | **495.7 (388.32 to 618.03)** | **-6.52 (-6.9 to -6.14)** |
| **Brazil** | **0.648847** | **NA** | **26709723 (21116993 to 32872183)** | **5664707 (4317930 to 7203070)** | **162249.13 (128275.9 to 199683.2)** | **34212.25 (26078.33 to 43503.26)** | **-5.93 (-6.32 to -5.54)** | **443414 (361841 to 536239)** | **83235 (64894 to 103822)** | **2693.53 (2198.01 to 3257.4)** | **502.7 (391.93 to 627.04)** | **-6.31 (-6.69 to -5.92)** |
| **Paraguay** | **0.650488** | **Middle SDI** | **2021129 (1864968 to 2169929)** | **148921 (115432 to 185677)** | **327565.98 (302256.84 to 351682.11)** | **22908.49 (17756.81 to 28562.73)** | **-9.9 (-10.7 to -9.1)** | **37348 (34597 to 40016)** | **2063 (1670 to 2529)** | **6053.02 (5607.09 to 6485.39)** | **317.28 (256.96 to 389.09)** | **-10.82 (-11.62 to -10.02)** |
| **North Africa and Middle East** | **0.658716** | **NA** | **106411110 (92650054 to 120824076)** | **32305754 (26760750 to 37972863)** | **207714.03 (180852.51 to 235848.08)** | **52841.49 (43771.71 to 62111)** | **-3.62 (-4.11 to -3.12)** | **1834656 (1652365 to 2027464)** | **481035 (417791 to 555114)** | **3581.24 (3225.41 to 3957.6)** | **786.81 (683.37 to 907.98)** | **-4.05 (-4.58 to -3.52)** |
| **North Africa and Middle East** | **0.658716** | **NA** | **106411110 (92650054 to 120824076)** | **32305754 (26760750 to 37972863)** | **207714.03 (180852.51 to 235848.08)** | **52841.49 (43771.71 to 62111)** | **-3.62 (-4.11 to -3.12)** | **1834656 (1652365 to 2027464)** | **481035 (417791 to 555114)** | **3581.24 (3225.41 to 3957.6)** | **786.81 (683.37 to 907.98)** | **-4.05 (-4.58 to -3.52)** |
| **Algeria** | **0.65972** | **Middle SDI** | **6970889 (5527707 to 8594987)** | **1603464 (1269986 to 1973055)** | **186587.99 (147958.71 to 230059.8)** | **34082.36 (26994.14 to 41938.2)** | **-5.46 (-5.8 to -5.12)** | **114684 (95188 to 138879)** | **22415 (18148 to 27175)** | **3069.7 (2547.89 to 3717.33)** | **476.43 (385.74 to 577.61)** | **-5.94 (-6.25 to -5.63)** |
| **Bahrain** | **0.752218** | **High-middle SDI** | **33349 (24258 to 43944)** | **25581 (18011 to 34341)** | **54298.28 (39497.43 to 71549.04)** | **27304.34 (19223.74 to 36654.12)** | **-2.18 (-2.54 to -1.82)** | **457 (345 to 589)** | **340 (247 to 459)** | **744.75 (562.05 to 959.49)** | **363.37 (263.7 to 490)** | **-2.25 (-2.61 to -1.88)** |
| **Egypt** | **0.603962** | **Low-middle SDI** | **21308629 (18863783 to 23482473)** | **9787172 (8353211 to 11343411)** | **249724.69 (221072.52 to 275200.87)** | **75069.98 (64071.15 to 87006.71)** | **-3.32 (-3.68 to -2.95)** | **399036 (370869 to 432781)** | **155357 (137196 to 177522)** | **4676.46 (4346.37 to 5071.94)** | **1191.63 (1052.32 to 1361.64)** | **-3.93 (-4.26 to -3.61)** |
| **Iran (Islamic Republic of)** | **0.697293** | **NA** | **17052577 (13475520 to 21249233)** | **1160109 (864204 to 1522555)** | **194395.24 (153617.66 to 242236.11)** | **18847.86 (14040.39 to 24736.38)** | **-7.36 (-7.7 to -7.03)** | **274833 (222327 to 336878)** | **16063 (12393 to 20618)** | **3133.03 (2534.48 to 3840.33)** | **260.98 (201.35 to 334.97)** | **-7.82 (-8.14 to -7.5)** |
| **Iraq** | **0.662777** | **Middle SDI** | **6077759 (4928815 to 7315560)** | **2061625 (1724769 to 2398142)** | **193390.56 (156831.87 to 232776.62)** | **48018.11 (40172.26 to 55856.07)** | **-4.51 (-5.08 to -3.93)** | **98320 (83108 to 116685)** | **29409 (25469 to 34106)** | **3128.49 (2644.45 to 3712.83)** | **684.99 (593.22 to 794.38)** | **-5 (-5.56 to -4.43)** |
| **Jordan** | **0.72542** | **High-middle SDI** | **658472 (550987 to 785396)** | **386401 (302820 to 479535)** | **109706.39 (91798.57 to 130852.88)** | **35213.31 (27596.42 to 43700.78)** | **-2.85 (-3.44 to -2.26)** | **9762 (8260 to 11428)** | **5310 (4320 to 6538)** | **1626.48 (1376.17 to 1904.02)** | **483.93 (393.72 to 595.83)** | **-3.06 (-3.66 to -2.46)** |
| **Kuwait** | **0.846802** | **High SDI** | **255692 (191742 to 324282)** | **113400 (79152 to 155091)** | **125063.04 (93784.23 to 158611.76)** | **42790.06 (29867.13 to 58521.88)** | **-2.21 (-2.54 to -1.89)** | **3803 (3038 to 4667)** | **1553 (1123 to 2104)** | **1860.04 (1485.74 to 2282.66)** | **586.03 (423.86 to 793.9)** | **-2.39 (-2.74 to -2.04)** |
| **Lebanon** | **0.741226** | **High-middle SDI** | **349102 (276911 to 437191)** | **211105 (155218 to 274477)** | **89602.46 (71073.45 to 112211.7)** | **52009.18 (38240.46 to 67621.94)** | **-2.12 (-2.73 to -1.51)** | **5102 (4139 to 6219)** | **2912 (2206 to 3742)** | **1309.54 (1062.26 to 1596.11)** | **717.39 (543.59 to 921.9)** | **-2.31 (-2.95 to -1.66)** |
| **Libya** | **0.735084** | **High-middle SDI** | **877475 (708261 to 1054324)** | **124644 (98225 to 156407)** | **137768.16 (111200.65 to 165534.45)** | **29461.5 (23216.81 to 36969.16)** | **-4.74 (-5.13 to -4.35)** | **13275 (11146 to 15604)** | **1704 (1382 to 2093)** | **2084.27 (1749.97 to 2449.83)** | **402.83 (326.58 to 494.8)** | **-5.05 (-5.44 to -4.66)** |
| **Morocco** | **0.56168** | **Low-middle SDI** | **9054517 (8035387 to 9963306)** | **1317128 (1124372 to 1524426)** | **254312.95 (225688.77 to 279837.96)** | **40517.15 (34587.66 to 46893.99)** | **-6.07 (-6.66 to -5.47)** | **168326 (155945 to 180669)** | **19851 (17333 to 23033)** | **4727.75 (4380 to 5074.43)** | **610.64 (533.18 to 708.54)** | **-6.73 (-7.26 to -6.2)** |
| **Palestine** | **0.629202** | **Middle SDI** | **310255 (251982 to 378544)** | **113890 (86142 to 146195)** | **79553.87 (64611.8 to 97064.15)** | **18586.54 (14058.08 to 23858.6)** | **-2.9 (-3.85 to -1.94)** | **4452 (3738 to 5354)** | **1531 (1211 to 1910)** | **1141.65 (958.57 to 1372.78)** | **249.92 (197.58 to 311.64)** | **-3.06 (-4.05 to -2.07)** |
| **Oman** | **0.773801** | **High-middle SDI** | **328699 (269227 to 387375)** | **104672 (80198 to 130659)** | **100311.77 (82162.27 to 118218.56)** | **24685.42 (18913.54 to 30814.16)** | **-3.71 (-4.26 to -3.16)** | **4903 (4202 to 5708)** | **1455 (1162 to 1782)** | **1496.41 (1282.51 to 1741.89)** | **343.13 (273.99 to 420.16)** | **-3.92 (-4.5 to -3.33)** |
| **Qatar** | **0.846704** | **High SDI** | **32478 (24624 to 41398)** | **66019 (50357 to 85916)** | **63927.15 (48468.1 to 81483.62)** | **35821.27 (27323.42 to 46617.24)** | **-1.77 (-1.95 to -1.59)** | **462 (366 to 582)** | **916 (714 to 1178)** | **908.98 (720.18 to 1144.78)** | **497.08 (387.17 to 638.98)** | **-1.82 (-1.99 to -1.64)** |
| **Saudi Arabia** | **0.814516** | **High SDI** | **3384426 (2745582 to 4085986)** | **820331 (600657 to 1104535)** | **139985.61 (113561.93 to 169003.31)** | **33715.28 (24686.77 to 45395.95)** | **-4 (-4.42 to -3.57)** | **52038 (44045 to 62106)** | **10880 (8068 to 14188)** | **2152.37 (1821.78 to 2568.82)** | **447.18 (331.6 to 583.11)** | **-4.43 (-4.89 to -3.97)** |
| **Syrian Arab Republic** | **0.622856** | **Middle SDI** | **3805605 (3141375 to 4499898)** | **368797 (299264 to 451146)** | **176435.13 (145640.14 to 208623.91)** | **36693.63 (29775.39 to 44887)** | **-4.86 (-5.3 to -4.42)** | **58837 (50541 to 68209)** | **5079 (4233 to 6079)** | **2727.81 (2343.19 to 3162.29)** | **505.32 (421.13 to 604.79)** | **-5.17 (-5.62 to -4.71)** |
| **Tunisia** | **0.681701** | **Middle SDI** | **1618828 (1317713 to 1970504)** | **436393 (335185 to 554069)** | **151695.4 (123478.78 to 184649.84)** | **48921.46 (37575.63 to 62113.49)** | **-2.93 (-3.18 to -2.68)** | **24503 (20646 to 28985)** | **5949 (4717 to 7524)** | **2296.09 (1934.67 to 2716.09)** | **666.86 (528.78 to 843.52)** | **-3.23 (-3.5 to -2.97)** |
| **Turkey** | **0.713246** | **High-middle SDI** | **12679797 (10493694 to 14961499)** | **1843056 (1420550 to 2371197)** | **182310.6 (150878.73 to 215117)** | **33197.02 (25586.87 to 42709.87)** | **-5.29 (-5.73 to -4.85)** | **199003 (171804 to 229020)** | **25084 (20100 to 31336)** | **2861.27 (2470.21 to 3292.87)** | **451.81 (362.04 to 564.43)** | **-5.79 (-6.19 to -5.38)** |
| **United Arab Emirates** | **0.84974** | **High SDI** | **191970 (147232 to 242684)** | **153431 (106863 to 209478)** | **84216.53 (64590.44 to 106464.93)** | **35460.86 (24698.03 to 48414.4)** | **-2.71 (-3.19 to -2.23)** | **2751 (2195 to 3379)** | **2104 (1542 to 2854)** | **1206.72 (962.88 to 1482.14)** | **486.19 (356.31 to 659.6)** | **-2.88 (-3.37 to -2.39)** |
| **Yemen** | **0.45354** | **Low SDI** | **8125869 (7521955 to 8783670)** | **3305475 (2818063 to 3818420)** | **292657.14 (270906.89 to 316348.16)** | **70369.61 (59993.2 to 81289.59)** | **-1.54 (-2.54 to -0.53)** | **155199 (142328 to 166587)** | **50493 (44278 to 58201)** | **5589.57 (5126 to 5999.72)** | **1074.93 (942.62 to 1239.03)** | **-2.01 (-3.15 to -0.85)** |
| **South Asia** | **0.559643** | **NA** | **385246785 (316264985 to 453056743)** | **106994261 (88613169 to 127455070)** | **245339.91 (201409.66 to 288523.89)** | **67464.36 (55874.31 to 80365.75)** | **-3.58 (-4 to -3.16)** | **5969095 (5059963 to 6980626)** | **1563595 (1331634 to 1845412)** | **3801.35 (3222.38 to 4445.53)** | **985.91 (839.65 to 1163.61)** | **-3.73 (-4.15 to -3.31)** |
| **South Asia** | **0.559643** | **NA** | **385246785 (316264985 to 453056743)** | **106994261 (88613169 to 127455070)** | **245339.91 (201409.66 to 288523.89)** | **67464.36 (55874.31 to 80365.75)** | **-3.58 (-4 to -3.16)** | **5969095 (5059963 to 6980626)** | **1563595 (1331634 to 1845412)** | **3801.35 (3222.38 to 4445.53)** | **985.91 (839.65 to 1163.61)** | **-3.73 (-4.15 to -3.31)** |
| **Afghanistan** | **0.335068** | **Low SDI** | **3024039 (2461055 to 3631828)** | **2802789 (2402879 to 3187285)** | **176413.96 (143571.02 to 211870.66)** | **51132.32 (43836.61 to 58146.83)** | **-3.93 (-4.57 to -3.29)** | **47871 (40472 to 57018)** | **40665 (36352 to 45702)** | **2792.65 (2361.05 to 3326.28)** | **741.87 (663.18 to 833.76)** | **-4.26 (-4.89 to -3.64)** |
| **Bangladesh** | **0.493106** | **Low-middle SDI** | **51570203 (43419301 to 59688152)** | **2382243 (1992966 to 2865518)** | **272412.47 (229356.46 to 315294.41)** | **16584.74 (13874.67 to 19949.22)** | **-7.81 (-8.41 to -7.2)** | **787209 (706701 to 891382)** | **32650 (28171 to 38073)** | **4158.32 (3733.05 to 4708.6)** | **227.3 (196.12 to 265.06)** | **-8.14 (-8.74 to -7.54)** |
| **Bhutan** | **0.476725** | **Low-middle SDI** | **152150 (122079 to 184099)** | **16504 (13404 to 19939)** | **159374.12 (127875.1 to 192840.13)** | **27042.12 (21962.75 to 32669.95)** | **-5.16 (-5.93 to -4.38)** | **2270 (1887 to 2732)** | **229 (188 to 270)** | **2378.1 (1976.68 to 2861.91)** | **374.77 (308.12 to 442.27)** | **-5.39 (-6.18 to -4.59)** |
| **India** | **0.577738** | **NA** | **272309063 (217021407 to 326668050)** | **78901534 (64241544 to 94997332)** | **234255.49 (186693.96 to 281018.14)** | **70867.76 (57700.46 to 85324.69)** | **-3.43 (-3.87 to -2.99)** | **4168510 (3440407 to 5009234)** | **1153008 (977692 to 1373126)** | **3585.99 (2959.63 to 4309.22)** | **1035.61 (878.14 to 1233.31)** | **-3.5 (-3.94 to -3.07)** |
| **Nepal** | **0.433953** | **Low SDI** | **8847119 (7709608 to 9795919)** | **865940 (743973 to 999100)** | **268633.65 (234094.28 to 297442.98)** | **27876.09 (23949.75 to 32162.71)** | **-6.03 (-6.78 to -5.29)** | **150095 (133274 to 166084)** | **12179 (10734 to 13725)** | **4557.49 (4046.73 to 5042.98)** | **392.05 (345.54 to 441.83)** | **-6.55 (-7.25 to -5.84)** |
| **Pakistan** | **0.504276** | **NA** | **52368250 (44067598 to 59798606)** | **24828040 (19887821 to 30092766)** | **283662.17 (238700.18 to 323910.05)** | **83522.99 (66903.8 to 101233.84)** | **-3 (-3.57 to -2.43)** | **861010 (735769 to 986395)** | **365529 (304323 to 437006)** | **4663.82 (3985.43 to 5342.99)** | **1229.66 (1023.76 to 1470.11)** | **-3.42 (-3.98 to -2.85)** |
| **Central Sub-Saharan Africa** | **0.484518** | **NA** | **29582048 (26117743 to 32538833)** | **16479501 (13759554 to 19269154)** | **284869.93 (251509.28 to 313343.25)** | **78226.09 (65314.85 to 91468.22)** | **-3.52 (-4.35 to -2.69)** | **527632 (478257 to 575017)** | **237055 (204775 to 275899)** | **5081 (4605.53 to 5537.31)** | **1125.27 (972.04 to 1309.66)** | **-4.34 (-5.23 to -3.44)** |
| **Angola** | **0.482946** | **Low-middle SDI** | **6201558 (5732062 to 6779898)** | **4646174 (3935581 to 5341376)** | **317675.98 (293625.94 to 347301.56)** | **82478.44 (69864.06 to 94819.61)** | **-4.12 (-4.87 to -3.36)** | **117715 (110139 to 126335)** | **66490 (58751 to 75159)** | **6029.95 (5641.9 to 6471.53)** | **1180.32 (1042.93 to 1334.22)** | **-5.26 (-6.04 to -4.46)** |
| **Central African Republic** | **0.311027** | **Low SDI** | **1467562 (1324468 to 1587602)** | **839217 (707103 to 980077)** | **291359.12 (262950.41 to 315191.13)** | **100044.66 (84295.04 to 116836.88)** | **-3.03 (-3.63 to -2.42)** | **26947 (24488 to 29181)** | **12179 (10755 to 13737)** | **5349.94 (4861.66 to 5793.43)** | **1451.87 (1282.18 to 1637.59)** | **-3.94 (-4.55 to -3.34)** |
| **Congo** | **0.586909** | **Low-middle SDI** | **1168738 (1031859 to 1289094)** | **487724 (408499 to 574958)** | **296264.1 (261566.5 to 326773.38)** | **77068.84 (64549.8 to 90853.22)** | **-3.58 (-4.36 to -2.8)** | **21282 (19150 to 23423)** | **7018 (6081 to 8148)** | **5394.89 (4854.28 to 5937.54)** | **1108.89 (960.9 to 1287.59)** | **-4.42 (-5.22 to -3.62)** |
| **Democratic Republic of the Congo** | **0.390178** | **Low SDI** | **20117791 (17351014 to 22486500)** | **10315864 (8416266 to 12268500)** | **275752.27 (237828.37 to 308219.89)** | **76071.54 (62063.47 to 90470.73)** | **-3.33 (-4.21 to -2.45)** | **351131 (310753 to 387700)** | **148672 (124643 to 175593)** | **4812.91 (4259.45 to 5314.16)** | **1096.34 (919.15 to 1294.86)** | **-4.04 (-5 to -3.06)** |
| **Equatorial Guinea** | **0.663978** | **Middle SDI** | **248348 (227140 to 268515)** | **79280 (65254 to 95153)** | **301905.69 (276124.34 to 326421.21)** | **42319.31 (34832.32 to 50792.13)** | **-6.31 (-7.24 to -5.36)** | **4626 (4272 to 5007)** | **1126 (952 to 1329)** | **5623.6 (5193.44 to 6087.2)** | **600.97 (508.02 to 709.41)** | **-7.32 (-8.18 to -6.46)** |
| **Gabon** | **0.639081** | **Middle SDI** | **378051 (305373 to 446920)** | **111241 (91311 to 134103)** | **242044.71 (195512.91 to 286137.51)** | **52093.94 (42760.65 to 62800.33)** | **-4.38 (-5.1 to -3.66)** | **5931 (5033 to 6937)** | **1570 (1329 to 1854)** | **3797.25 (3222.39 to 4441.23)** | **735.39 (622.27 to 868.23)** | **-4.73 (-5.46 to -4)** |
| **Eastern Sub-Saharan Africa** | **0.412188** | **NA** | **103603831 (91552402 to 113911597)** | **42046037 (36246261 to 48452569)** | **287099.13 (253703.12 to 315663.24)** | **65906.62 (56815.54 to 75948.77)** | **-4.28 (-5.07 to -3.49)** | **1814230 (1651113 to 1988477)** | **621489 (552903 to 703275)** | **5027.46 (4575.44 to 5510.32)** | **974.18 (866.67 to 1102.37)** | **-4.9 (-5.67 to -4.12)** |
| **Burundi** | **0.291289** | **Low SDI** | **2915445 (2488908 to 3315759)** | **1813025 (1536426 to 2139395)** | **271179.03 (231504.92 to 308414.18)** | **83921.34 (71118.1 to 99028.33)** | **-3.1 (-3.99 to -2.2)** | **47435 (41710 to 53258)** | **26367 (23059 to 30268)** | **4412.13 (3879.64 to 4953.79)** | **1220.48 (1067.35 to 1401.06)** | **-3.48 (-4.47 to -2.48)** |
| **Comoros** | **0.476956** | **Low-middle SDI** | **234570 (198264 to 267065)** | **56531 (47625 to 65545)** | **284612.17 (240560.65 to 324038.94)** | **69527.2 (58573.83 to 80613.93)** | **-4.17 (-5.01 to -3.33)** | **4089 (3558 to 4636)** | **833 (727 to 953)** | **4961 (4316.67 to 5625.47)** | **1025.07 (894.71 to 1172.55)** | **-4.8 (-5.67 to -3.91)** |
| **Djibouti** | **0.4892** | **Low-middle SDI** | **184236 (158004 to 207474)** | **64662 (53183 to 76940)** | **285068.03 (244479.39 to 321024.63)** | **44381.41 (36502.61 to 52808.4)** | **-6.1 (-6.58 to -5.61)** | **3200 (2837 to 3636)** | **915 (771 to 1071)** | **4952.06 (4389.69 to 5626)** | **628.28 (529.33 to 735.06)** | **-6.71 (-7.13 to -6.28)** |
| **Eritrea** | **0.404572** | **Low SDI** | **1938007 (1739989 to 2103594)** | **660497 (564731 to 770552)** | **310829.96 (279070.63 to 337387.89)** | **71946.71 (61515.09 to 83934.83)** | **-4.44 (-5.17 to -3.71)** | **34188 (31340 to 37083)** | **9423 (8376 to 10607)** | **5483.32 (5026.46 to 5947.54)** | **1026.48 (912.38 to 1155.37)** | **-5.09 (-5.77 to -4.41)** |
| **Ethiopia** | **0.360728** | **NA** | **28130198 (23791048 to 32367863)** | **13356817 (11066420 to 15866438)** | **291827.78 (246812.65 to 335790.09)** | **83662.51 (69316.25 to 99381.92)** | **-3.68 (-4.35 to -3.01)** | **474364 (402022 to 545945)** | **198612 (169464 to 232443)** | **4921.14 (4170.65 to 5663.73)** | **1244.04 (1061.46 to 1455.94)** | **-4.18 (-4.85 to -3.5)** |
| **Kenya** | **0.524783** | **NA** | **11118369 (9275033 to 12868126)** | **5365656 (4524270 to 6282748)** | **258988.87 (216050.6 to 299747.33)** | **90176.3 (76035.79 to 105589.13)** | **-3.23 (-3.81 to -2.65)** | **186375 (158806 to 214844)** | **85012 (74108 to 97422)** | **4341.37 (3699.19 to 5004.53)** | **1428.74 (1245.47 to 1637.29)** | **-3.45 (-4.02 to -2.87)** |
| **Madagascar** | **0.401385** | **Low SDI** | **7455577 (6964425 to 7875123)** | **3773949 (3205115 to 4424692)** | **345870.39 (323085.45 to 365333.46)** | **92316.41 (78401.89 to 108234.53)** | **-3.72 (-4.41 to -3.03)** | **138009 (129291 to 146637)** | **54473 (47657 to 60774)** | **6402.35 (5997.93 to 6802.59)** | **1332.49 (1165.76 to 1486.61)** | **-4.59 (-5.27 to -3.91)** |
| **Malawi** | **0.381986** | **Low SDI** | **5677282 (5191643 to 6185619)** | **1709282 (1465710 to 1957486)** | **299004.39 (273427.36 to 325776.89)** | **62747.71 (53806.16 to 71859.26)** | **-4.49 (-5.49 to -3.48)** | **105607 (97910 to 113280)** | **24526 (21857 to 27811)** | **5561.97 (5156.59 to 5966.12)** | **900.33 (802.38 to 1020.94)** | **-5.45 (-6.46 to -4.42)** |
| **Mauritius** | **0.717977** | **High-middle SDI** | **240951 (209228 to 275844)** | **60666 (47973 to 74010)** | **228455.35 (198376.88 to 261538.69)** | **94355.68 (74614.03 to 115109.51)** | **-2.08 (-2.58 to -1.58)** | **4051 (3616 to 4494)** | **873 (720 to 1043)** | **3841.3 (3428.74 to 4260.67)** | **1357.43 (1120.04 to 1621.84)** | **-2.39 (-2.9 to -1.88)** |
| **Mozambique** | **0.327475** | **Low SDI** | **6893596 (6285508 to 7413921)** | **1572416 (1330116 to 1831957)** | **286159.7 (260917.38 to 307758.88)** | **30360.11 (25681.79 to 35371.31)** | **-7.08 (-8 to -6.15)** | **128496 (118487 to 138018)** | **22835 (20094 to 26114)** | **5333.99 (4918.49 to 5729.28)** | **440.9 (387.97 to 504.21)** | **-8 (-8.85 to -7.15)** |
| **Rwanda** | **0.43614** | **Low SDI** | **3988846 (3637353 to 4361060)** | **767673 (655968 to 890155)** | **295938.35 (269860.58 to 323553.47)** | **43905.59 (37516.86 to 50910.76)** | **-5.73 (-6.87 to -4.58)** | **72947 (67122 to 78280)** | **11006 (9670 to 12570)** | **5412.06 (4979.88 to 5807.67)** | **629.45 (553.03 to 718.9)** | **-6.67 (-7.83 to -5.5)** |
| **Seychelles** | **0.727579** | **High-middle SDI** | **6782 (5484 to 8151)** | **3550 (2777 to 4387)** | **84167.7 (68050.37 to 101151.42)** | **45088.38 (35268.63 to 55709.5)** | **-1.28 (-1.66 to -0.91)** | **97 (81 to 115)** | **50 (40 to 61)** | **1206.22 (1006.86 to 1430.37)** | **629.24 (504.65 to 771.34)** | **-1.36 (-1.74 to -0.98)** |
| **Somalia** | **0.077434** | **Low SDI** | **4144916 (3538292 to 4625801)** | **2411304 (2070605 to 2786641)** | **268272.61 (229009.93 to 299397.08)** | **58398.22 (50147 to 67488.32)** | **-4.29 (-5.22 to -3.34)** | **69328 (60761 to 78336)** | **35326 (31349 to 40025)** | **4487.11 (3932.62 to 5070.17)** | **855.55 (759.24 to 969.34)** | **-4.75 (-5.67 to -3.81)** |
| **United Republic of Tanzania** | **0.448566** | **Low SDI** | **13126935 (11308121 to 14813275)** | **3074334 (2603003 to 3595364)** | **273021.37 (235192.65 to 308094.81)** | **34753.3 (29425.22 to 40643.2)** | **-5.95 (-7.02 to -4.88)** | **229895 (203773 to 254619)** | **43286 (37969 to 49782)** | **4781.49 (4238.18 to 5295.71)** | **489.31 (429.22 to 562.75)** | **-6.71 (-7.71 to -5.69)** |
| **Uganda** | **0.426554** | **Low SDI** | **10200776 (8943703 to 11410724)** | **3883219 (3364476 to 4438168)** | **284028.25 (249026.58 to 317717.78)** | **53065.98 (45977.12 to 60649.61)** | **-4.55 (-5.47 to -3.62)** | **181592 (164142 to 200624)** | **55881 (49743 to 62871)** | **5056.22 (4570.33 to 5586.13)** | **763.64 (679.76 to 859.16)** | **-5.24 (-6.11 to -4.36)** |
| **Zambia** | **0.51023** | **Low-middle SDI** | **4412787 (4058055 to 4748863)** | **1653236 (1416795 to 1927338)** | **292398.83 (268893.64 to 314667.78)** | **56520.16 (48436.83 to 65891.03)** | **-4.83 (-5.83 to -3.82)** | **81931 (76091 to 87913)** | **24600 (21628 to 27813)** | **5428.86 (5041.94 to 5825.24)** | **841 (739.42 to 950.87)** | **-5.7 (-6.66 to -4.73)** |
| **Southern Sub-Saharan Africa** | **0.643348** | **NA** | **20643922 (17798334 to 22955093)** | **4670596 (3873116 to 5540730)** | **276250.38 (238171.64 to 307177.75)** | **58170.76 (48238.4 to 69008)** | **-4.85 (-5.1 to -4.6)** | **343520 (298063 to 387092)** | **71871 (61907 to 83339)** | **4596.87 (3988.59 to 5179.94)** | **895.13 (771.03 to 1037.96)** | **-5.09 (-5.35 to -4.82)** |
| **Botswana** | **0.643078** | **Middle SDI** | **491310 (413896 to 573631)** | **177685 (145754 to 207516)** | **230638.36 (194297.21 to 269282.87)** | **75450.67 (61891.85 to 88117.72)** | **-3.22 (-3.76 to -2.67)** | **7876 (6940 to 8847)** | **2623 (2295 to 2999)** | **3697.17 (3257.8 to 4152.87)** | **1113.76 (974.46 to 1273.6)** | **-3.55 (-4.15 to -2.95)** |
| **Lesotho** | **0.511571** | **Low-middle SDI** | **646938 (543167 to 749838)** | **122288 (103625 to 141268)** | **262658.83 (220527.36 to 304436.81)** | **60029.24 (50867.65 to 69345.94)** | **-4.6 (-5.06 to -4.14)** | **10993 (9756 to 12407)** | **1888 (1681 to 2125)** | **4463.27 (3961.11 to 5037.39)** | **926.7 (825.14 to 1043.02)** | **-4.95 (-5.49 to -4.41)** |
| **Namibia** | **0.618074** | **Low-middle SDI** | **549629 (443078 to 649393)** | **161193 (132052 to 190475)** | **242812.94 (195741.09 to 286886.25)** | **57889.04 (47423.68 to 68405.2)** | **-3.98 (-4.58 to -3.38)** | **8608 (7348 to 10048)** | **2401 (2087 to 2729)** | **3803.01 (3246.33 to 4438.99)** | **862.41 (749.62 to 980.17)** | **-4.14 (-4.78 to -3.49)** |
| **South Africa** | **0.681292** | **NA** | **15158317 (13030904 to 16820474)** | **3368206 (2724061 to 4104338)** | **310731.36 (267121.37 to 344804.02)** | **67889.38 (54906.03 to 82726.81)** | **-5.09 (-5.21 to -4.98)** | **255995 (219733 to 288130)** | **52732 (44349 to 62663)** | **5247.66 (4504.32 to 5906.39)** | **1062.86 (893.89 to 1263.04)** | **-5.27 (-5.37 to -5.17)** |
| **Eswatini** | **0.586217** | **Low-middle SDI** | **392722 (325929 to 459772)** | **88980 (75843 to 102334)** | **270069.76 (224137.31 to 316179.55)** | **63368.66 (54013.12 to 72879.03)** | **-4.51 (-5.1 to -3.91)** | **6638 (5827 to 7579)** | **1424 (1268 to 1582)** | **4564.55 (4007.19 to 5211.9)** | **1013.94 (902.84 to 1126.52)** | **-4.69 (-5.34 to -4.03)** |
| **Zimbabwe** | **0.475577** | **Low-middle SDI** | **3405005 (2810786 to 4051941)** | **752244 (622819 to 870150)** | **193078.72 (159383.88 to 229762.83)** | **34042.53 (28185.45 to 39378.33)** | **-4.24 (-5.01 to -3.47)** | **53409 (45710 to 61533)** | **10803 (9259 to 12345)** | **3028.55 (2591.93 to 3489.16)** | **488.91 (419.03 to 558.66)** | **-4.61 (-5.4 to -3.8)** |
| **Western Sub-Saharan Africa** | **0.446421** | **NA** | **108088161 (96715647 to 117694048)** | **69823406 (58810866 to 81693193)** | **302392.13 (270575.89 to 329265.97)** | **87324.99 (73552.1 to 102170)** | **-3.5 (-3.99 to -3.02)** | **1881143 (1695781 to 2050173)** | **1021638 (895315 to 1177219)** | **5262.77 (4744.19 to 5735.65)** | **1277.72 (1119.73 to 1472.29)** | **-4.12 (-4.6 to -3.64)** |
| **Benin** | **0.374522** | **Low SDI** | **2603530 (2178129 to 2990673)** | **1187000 (987426 to 1388628)** | **263858.68 (220745.78 to 303094.31)** | **50675.33 (42155.12 to 59283.21)** | **-4.91 (-5.39 to -4.42)** | **40639 (35118 to 46910)** | **16659 (14247 to 19092)** | **4118.61 (3559.04 to 4754.13)** | **711.19 (608.22 to 815.05)** | **-5.26 (-5.75 to -4.77)** |
| **Burkina Faso** | **0.284471** | **Low SDI** | **5599842 (5073837 to 6067782)** | **2503443 (2092267 to 2909938)** | **298415.52 (270384.75 to 323352.03)** | **60963.45 (50950.56 to 70862.36)** | **-4.76 (-5.66 to -3.86)** | **103233 (94803 to 110921)** | **35686 (31097 to 40804)** | **5501.3 (5052.05 to 5910.95)** | **869.02 (757.27 to 993.64)** | **-5.77 (-6.7 to -4.84)** |
| **Cameroon** | **0.480365** | **Low-middle SDI** | **5676321 (4965873 to 6244905)** | **3411861 (2847953 to 4000898)** | **282910.48 (247501.39 to 311248.95)** | **70149.3 (58555.11 to 82260.14)** | **-3.72 (-4.56 to -2.86)** | **97860 (87588 to 107917)** | **49663 (42866 to 57262)** | **4877.38 (4365.44 to 5378.64)** | **1021.1 (881.35 to 1177.32)** | **-4.33 (-5.19 to -3.46)** |
| **Cabo Verde** | **0.533601** | **Low-middle SDI** | **154284 (133082 to 174358)** | **9684 (7881 to 11897)** | **259272.72 (223643.18 to 293007.43)** | **21979.29 (17887.88 to 27002.97)** | **-8.71 (-9.43 to -7.97)** | **2595 (2332 to 2874)** | **133 (111 to 159)** | **4360.47 (3919.55 to 4830.32)** | **301.6 (252.08 to 361.32)** | **-9.49 (-10.29 to -8.69)** |
| **Chad** | **0.243517** | **Low SDI** | **3794099 (3285169 to 4236053)** | **4222344 (3601567 to 4889890)** | **305257.24 (264310.89 to 340815)** | **116141.55 (99066.22 to 134503.36)** | **-2.24 (-2.78 to -1.7)** | **64963 (57648 to 72167)** | **61353 (54867 to 68496)** | **5226.63 (4638.1 to 5806.26)** | **1687.61 (1509.19 to 1884.07)** | **-2.86 (-3.46 to -2.26)** |
| **C么te d'Ivoire** | **0.424541** | **Low SDI** | **5764617 (4743198 to 6724061)** | **2396923 (1984610 to 2797932)** | **249501.68 (205293.03 to 291027.92)** | **55046.12 (45577.2 to 64255.41)** | **-4.45 (-5.18 to -3.72)** | **91817 (79156 to 104543)** | **33946 (28926 to 39139)** | **3973.99 (3426 to 4524.8)** | **779.58 (664.29 to 898.83)** | **-4.92 (-5.64 to -4.19)** |
| **Gambia** | **0.410077** | **Low SDI** | **419864 (350129 to 484706)** | **100415 (83691 to 120736)** | **226108.54 (188553.84 to 261027.41)** | **28081.09 (23404.18 to 33763.91)** | **-6.25 (-7.5 to -4.98)** | **6242 (5389 to 7085)** | **1375 (1170 to 1611)** | **3361.46 (2902.38 to 3815.67)** | **384.46 (327.25 to 450.53)** | **-6.54 (-7.83 to -5.23)** |
| **Ghana** | **0.563348** | **Low-middle SDI** | **7883248 (7227195 to 8503920)** | **1790900 (1517308 to 2081465)** | **299286.53 (274379.54 to 322850.25)** | **38595.86 (32699.66 to 44857.85)** | **-6.09 (-6.9 to -5.27)** | **144558 (133770 to 155675)** | **25176 (21905 to 28632)** | **5488.14 (5078.56 to 5910.2)** | **542.57 (472.07 to 617.05)** | **-6.85 (-7.59 to -6.11)** |
| **Guinea** | **0.336555** | **Low SDI** | **3287670 (2789485 to 3729117)** | **1020663 (855241 to 1199031)** | **283287.74 (240360.77 to 321325.74)** | **45353.22 (38002.67 to 53278.99)** | **-5.61 (-6.35 to -4.86)** | **52713 (46020 to 59506)** | **14206 (12209 to 16256)** | **4542.11 (3965.43 to 5127.46)** | **631.26 (542.53 to 722.35)** | **-6.1 (-6.83 to -5.37)** |
| **Guinea-Bissau** | **0.353448** | **Low SDI** | **496961 (413874 to 573260)** | **140196 (117550 to 167795)** | **264233.52 (220056.22 to 304802.04)** | **42144.53 (35336.86 to 50441.1)** | **-5.68 (-6.46 to -4.89)** | **7608 (6520 to 8743)** | **1957 (1653 to 2291)** | **4045.21 (3466.82 to 4648.47)** | **588.26 (496.89 to 688.74)** | **-5.98 (-6.78 to -5.19)** |
| **Liberia** | **0.353229** | **Low SDI** | **1382093 (1213471 to 1525850)** | **553907 (468609 to 652042)** | **297592.87 (261284.98 to 328546.66)** | **72275.02 (61145.08 to 85079.84)** | **-3.61 (-4.54 to -2.66)** | **25095 (22539 to 27538)** | **7966 (6909 to 9244)** | **5403.43 (4853.02 to 5929.44)** | **1039.43 (901.45 to 1206.15)** | **-4.54 (-5.5 to -3.57)** |
| **Mali** | **0.271176** | **Low SDI** | **5450591 (4901171 to 5928847)** | **2243828 (1944162 to 2561504)** | **315213.74 (283440.2 to 342871.85)** | **48987.81 (42445.42 to 55923.38)** | **-5.42 (-6.26 to -4.58)** | **95627 (86104 to 105014)** | **31988 (28602 to 35687)** | **5530.19 (4979.49 to 6073.07)** | **698.38 (624.45 to 779.14)** | **-6.21 (-7.02 to -5.39)** |
| **Mauritania** | **0.495267** | **Low-middle SDI** | **924565 (770139 to 1070732)** | **467253 (386699 to 550752)** | **249245.66 (207615.41 to 288649.78)** | **71118.96 (58858.1 to 83828.05)** | **-3.29 (-4.1 to -2.47)** | **14608 (12553 to 16825)** | **6704 (5743 to 7711)** | **3938.01 (3384.16 to 4535.68)** | **1020.45 (874.18 to 1173.63)** | **-3.71 (-4.57 to -2.85)** |
| **Niger** | **0.17031** | **Low SDI** | **5435773 (5008359 to 5903423)** | **4872179 (4137222 to 5653154)** | **322217.45 (296881.51 to 349938.4)** | **95601.51 (81180.24 to 110925.74)** | **-3.45 (-4.15 to -2.76)** | **102561 (96714 to 109526)** | **70129 (62163 to 78993)** | **6079.5 (5732.93 to 6492.4)** | **1376.06 (1219.75 to 1549.99)** | **-4.49 (-5.25 to -3.73)** |
| **Nigeria** | **0.503699** | **NA** | **50019372 (43815612 to 54921234)** | **41696458 (34324131 to 50072015)** | **314682.44 (275653.28 to 345521.1)** | **112335.35 (92473.4 to 134900.12)** | **-2.85 (-3.15 to -2.55)** | **867048 (758774 to 968448)** | **619632 (527663 to 733525)** | **5454.78 (4773.61 to 6092.71)** | **1669.37 (1421.59 to 1976.21)** | **-3.39 (-3.69 to -3.09)** |
| **Sao Tome and Principe** | **0.503306** | **Low-middle SDI** | **51632 (42793 to 60035)** | **3413 (2789 to 4156)** | **252103.27 (208943.08 to 293133.52)** | **13681.6 (11181.41 to 16659.78)** | **-8.2 (-9.37 to -7.01)** | **827 (719 to 944)** | **47 (39 to 56)** | **4037.9 (3512.19 to 4606.83)** | **187.31 (156.72 to 222.93)** | **-8.74 (-9.89 to -7.57)** |
| **Senegal** | **0.409005** | **Low SDI** | **4588015 (4219756 to 4969578)** | **1471967 (1232669 to 1704280)** | **313163.54 (288027.3 to 339207.78)** | **64803.04 (54267.98 to 75030.58)** | **-4.28 (-5.15 to -3.41)** | **81982 (76075 to 87736)** | **20516 (17869 to 23533)** | **5595.85 (5192.64 to 5988.6)** | **903.23 (786.7 to 1036.04)** | **-5.1 (-5.93 to -4.26)** |
| **Sierra Leone** | **0.359009** | **Low SDI** | **2389688 (2100005 to 2651585)** | **759661 (606177 to 917238)** | **308826.56 (271389.89 to 342672.29)** | **56566.5 (45137.67 to 68300.14)** | **-5.08 (-5.96 to -4.18)** | **42653 (38108 to 47041)** | **10544 (8659 to 12689)** | **5512.12 (4924.86 to 6079.24)** | **785.1 (644.79 to 944.86)** | **-5.98 (-6.82 to -5.13)** |
| **Togo** | **0.410016** | **Low SDI** | **2162428 (1952027 to 2364421)** | **970498 (814434 to 1140879)** | **315085.9 (284428.51 to 344518.26)** | **82771 (69460.72 to 97302.34)** | **-3.82 (-4.46 to -3.16)** | **38453 (34816 to 42275)** | **13945 (12246 to 16011)** | **5603 (5072.97 to 6159.92)** | **1189.37 (1044.45 to 1365.56)** | **-4.56 (-5.19 to -3.92)** |
| **American Samoa** | **0.726268** | **High-middle SDI** | **5144 (4076 to 6308)** | **2183 (1655 to 2769)** | **67485.95 (53478.81 to 82764.69)** | **58848.91 (44630.14 to 74639.45)** | **-0.39 (-0.58 to -0.2)** | **72 (59 to 89)** | **30 (24 to 38)** | **950.27 (772.47 to 1166.79)** | **808.12 (640.86 to 1011.59)** | **-0.46 (-0.65 to -0.27)** |
| **Bermuda** | **0.82132** | **High SDI** | **2066 (1518 to 2715)** | **1289 (856 to 1821)** | **48099.46 (35344.37 to 63197.74)** | **50523.02 (33553.57 to 71370.46)** | **0.06 (-0.24 to 0.35)** | **28 (21 to 36)** | **17 (12 to 24)** | **655.3 (491.66 to 836.62)** | **679.53 (466 to 939.64)** | **0.02 (-0.29 to 0.33)** |
| **Cook Islands** | **0.778252** | **High-middle SDI** | **1580 (1157 to 2102)** | **916 (627 to 1234)** | **70544.26 (51666.08 to 93844.47)** | **81462.16 (55807.48 to 109728.09)** | **0.55 (0.43 to 0.68)** | **22 (16 to 28)** | **12 (9 to 17)** | **981.62 (732.05 to 1271.69)** | **1097.93 (788.5 to 1484.24)** | **0.48 (0.33 to 0.62)** |
| **Greenland** | **0.83564** | **High SDI** | **892 (631 to 1196)** | **208 (147 to 284)** | **16199.06 (11453.3 to 21726.76)** | **5141.83 (3625.89 to 7038.77)** | **-3.76 (-4.29 to -3.23)** | **13 (10 to 18)** | **3 (2 to 4)** | **238.58 (175.65 to 319.11)** | **75.79 (54.85 to 102.32)** | **-3.75 (-4.28 to -3.22)** |
| **Guam** | **0.802168** | **High-middle SDI** | **11464 (8592 to 14603)** | **10369 (7821 to 12959)** | **70884.59 (53128.76 to 90289.93)** | **81137.95 (61200.62 to 101405.27)** | **0.79 (0.46 to 1.12)** | **159 (123 to 199)** | **143 (114 to 174)** | **980.75 (761.25 to 1227.53)** | **1117.5 (889.65 to 1363.84)** | **0.81 (0.46 to 1.16)** |
| **Monaco** | **0.909519** | **High SDI** | **1103 (754 to 1515)** | **1467 (1007 to 2031)** | **94211.14 (64351.37 to 129375.81)** | **90678.88 (62228.91 to 125496.44)** | **0.24 (-0.3 to 0.78)** | **17 (12 to 23)** | **23 (16 to 32)** | **1414.76 (1013.27 to 1973.62)** | **1405.3 (991.91 to 1947.34)** | **0.33 (-0.19 to 0.85)** |
| **Nauru** | **0.62755** | **Middle SDI** | **1349 (1073 to 1660)** | **853 (691 to 1055)** | **82541.03 (65671.96 to 101613.79)** | **61046.46 (49429.14 to 75472.06)** | **-1.17 (-1.42 to -0.92)** | **20 (16 to 24)** | **12 (10 to 14)** | **1201.02 (996.24 to 1448.81)** | **857.54 (719.31 to 1033.07)** | **-1.3 (-1.56 to -1.03)** |
| **Niue** | **0.726219** | **High-middle SDI** | **215 (171 to 271)** | **81 (65 to 100)** | **84311.1 (66892.39 to 106437.22)** | **68791.21 (55213.24 to 85002.96)** | **-0.39 (-0.81 to 0.03)** | **3 (3 to 4)** | **1 (1 to 1)** | **1226.07 (1002.07 to 1496.59)** | **993.11 (831.71 to 1186.37)** | **-0.43 (-0.86 to 0)** |
| **Northern Mariana Islands** | **0.777505** | **High-middle SDI** | **3603 (2732 to 4613)** | **2470 (1900 to 3129)** | **75823.39 (57478.91 to 97067.44)** | **77032.43 (59240.91 to 97566.69)** | **0 (-0.14 to 0.14)** | **50 (40 to 63)** | **34 (27 to 42)** | **1060.7 (835.51 to 1315.58)** | **1064.74 (848.9 to 1300.25)** | **-0.04 (-0.18 to 0.11)** |
| **Palau** | **0.75459** | **High-middle SDI** | **1665 (1345 to 2031)** | **541 (432 to 658)** | **112713.73 (91044.14 to 137553.42)** | **57076.18 (45624.11 to 69464.11)** | **-2.33 (-2.56 to -2.1)** | **25 (21 to 30)** | **8 (6 to 9)** | **1677.2 (1396.92 to 2025.97)** | **802.25 (661.29 to 965.02)** | **-2.48 (-2.71 to -2.26)** |
| **Puerto Rico** | **0.824544** | **High SDI** | **179341 (120023 to 250940)** | **41886 (29318 to 56360)** | **56185.24 (37601.73 to 78616.36)** | **39845.62 (27889.81 to 53614.43)** | **-0.91 (-1.4 to -0.41)** | **2467 (1667 to 3400)** | **570 (409 to 762)** | **772.88 (522.35 to 1065.17)** | **542.03 (388.81 to 725.22)** | **-0.93 (-1.43 to -0.43)** |
| **Saint Kitts and Nevis** | **0.756333** | **High-middle SDI** | **3004 (2535 to 3465)** | **885 (711 to 1075)** | **64103.67 (54098.87 to 73959.31)** | **29013.96 (23291.68 to 35232.02)** | **-2.67 (-2.84 to -2.51)** | **43 (37 to 49)** | **12 (10 to 15)** | **913.19 (788.88 to 1038.53)** | **397.76 (332.29 to 475.41)** | **-2.8 (-2.96 to -2.64)** |
| **San Marino** | **0.887884** | **High SDI** | **1021 (708 to 1396)** | **1004 (702 to 1349)** | **86042.46 (59666.48 to 117656.26)** | **83066.54 (58026.53 to 111549.31)** | **0.38 (-0.16 to 0.92)** | **15 (11 to 21)** | **15 (11 to 21)** | **1288 (917.48 to 1796.21)** | **1273.37 (900.01 to 1742.28)** | **0.45 (-0.07 to 0.97)** |
| **Tokelau** | **0.687018** | **Middle SDI** | **158 (127 to 195)** | **48 (37 to 63)** | **81074.77 (65192.15 to 100403.1)** | **48840.43 (37161.45 to 63685.4)** | **-1.69 (-1.86 to -1.53)** | **2 (2 to 3)** | **1 (1 to 1)** | **1175.73 (971.21 to 1430.93)** | **655.87 (517 to 818.52)** | **-1.94 (-2.12 to -1.77)** |
| **Tuvalu** | **0.578627** | **Low-middle SDI** | **1742 (1401 to 2162)** | **499 (395 to 608)** | **115435.32 (92892.55 to 143295.73)** | **38875.07 (30793.82 to 47393.36)** | **-3.51 (-3.65 to -3.36)** | **26 (22 to 32)** | **7 (6 to 8)** | **1742.1 (1453.1 to 2096.09)** | **528.81 (434.57 to 639.83)** | **-3.83 (-3.99 to -3.68)** |
| **United States Virgin Islands** | **0.822988** | **High SDI** | **4876 (3524 to 6438)** | **1003 (669 to 1401)** | **44295.74 (32012.65 to 58480.33)** | **25602.68 (17072.1 to 35744.02)** | **-1.72 (-2.12 to -1.32)** | **66 (49 to 86)** | **13 (9 to 18)** | **597.73 (443.06 to 776.88)** | **333.62 (224.51 to 466.99)** | **-1.82 (-2.22 to -1.42)** |
| **South Sudan** | **0.278378** | **Low SDI** | **3108250 (2743581 to 3442815)** | **1846832 (1583909 to 2106069)** | **305387.35 (269558.47 to 338258.56)** | **118209.83 (101380.92 to 134802.76)** | **-2.51 (-3.19 to -1.83)** | **55479 (49704 to 61456)** | **27851 (25007 to 31111)** | **5450.81 (4883.41 to 6038.12)** | **1782.65 (1600.63 to 1991.32)** | **-3.05 (-3.78 to -2.32)** |
| **Sudan** | **0.542748** | **Low-middle SDI** | **10212471 (9537307 to 10950893)** | **5470140 (4529239 to 6428945)** | **293386.92 (273990.6 to 314600.51)** | **97004.26 (80318.88 to 114007.15)** | **-3.17 (-3.96 to -2.38)** | **196035 (182679 to 208079)** | **81516 (70011 to 94593)** | **5631.74 (5248.04 to 5977.76)** | **1445.55 (1241.54 to 1677.46)** | **-4.1 (-4.98 to -3.22)** |
| **Georgia** | **0.847268** | **High SDI** | **480986 (414627 to 553783)** | **58791 (46170 to 72314)** | **102651.2 (88488.84 to 118187.24)** | **24164.65 (18976.9 to 29722.91)** | **-4.26 (-5.02 to -3.5)** | **7657 (6778 to 8602)** | **813 (651 to 984)** | **1634.18 (1446.56 to 1835.93)** | **334.12 (267.73 to 404.6)** | **-4.69 (-5.43 to -3.94)** |
| **Niger** | **0.425189** | **Low SDI** | **5435773 (5008359 to 5903423)** | **4872179 (4137222 to 5653154)** | **322217.45 (296881.51 to 349938.4)** | **95601.51 (81180.24 to 110925.74)** | **-3.45 (-4.15 to -2.76)** | **102561 (96714 to 109526)** | **70129 (62163 to 78993)** | **6079.5 (5732.93 to 6492.4)** | **1376.06 (1219.75 to 1549.99)** | **-4.49 (-5.25 to -3.73)** |

**Abbreviations: ASR, age-standardized rate; DALYs, disability-adjusted life-years; SDI, sociodemographic index; GBD, Global Burden of Diseases, Injuries, and Risk Factors Study; EAPC, estimated annual percentage change; UIs, uncertainty intervals; CI, conﬁdence interval.**
