# Supplementary material for: Global trends, inequalities, and pathogen shifts in infectious diarrhea among children under five: a comprehensive analysis of the global burden of disease study 1990–2021
Source: Front Nutr. 2025 Nov 14;12:1679081. doi: 10.3389/fnut.2025.1679081 (PMC12661344; doi:10.3389/fnut.2025.1679081)
Supplement: Supplementary file 2 [file Table_2.docx]

**Table S2. The case number and ASR of Death and DALYs of infectious diarrhea in children under 5 years of age in 1990 and 2021 for both sexes by SDI quintiles, by GBD regions and 204 countries and regions, with EAPC from 1990 to 2021.**

|  | **SDI** | | **Death** | | | | | **DALYs** | | | | |
| --- | --- | --- | --- | --- | --- | --- | --- | --- | --- | --- | --- | --- |
| **Location** | **SDI.Index.Value** | **SDI.Quintile** | **Number (95 % UIs).1990** | **Number (95 % UIs).2021** | **ASR (95 % UIs) .1990** | **ASR (95 % UIs) .2021** | **EAPC (95 % CI) 1990–2021** | **Number (95 % UIs).1990** | **Number (95 % UIs).2021** | **ASR (95 % UIs) .1990** | **ASR (95 % UIs) .2021** | **EAPC (95 % CI) 1990–2021** |
| **Global** | **0.666368** | **NA** | **1636314 (1285402 to 1930943)** | **340429 (250952 to 464258)** | **263.95 (207.34 to 311.47)** | **51.72 (38.13 to 70.54)** | **-5.06 (-5.36 to -4.76)** | **147785002 (116823041 to 173680475)** | **30931280 (23118226 to 41966936)** | **23838.68 (18844.31 to 28015.78)** | **4699.58 (3512.49 to 6376.29)** | **-5.04 (-5.34 to -4.73)** |
| **High SDI** | **NA** | **NA** | **2089 (1618 to 2821)** | **425 (367 to 480)** | **3.39 (2.62 to 4.57)** | **0.79 (0.68 to 0.89)** | **-4.14 (-4.32 to -3.96)** | **254083 (202409 to 321586)** | **76190 (61696 to 95371)** | **411.72 (327.99 to 521.11)** | **141.5 (114.58 to 177.12)** | **-2.8 (-3.02 to -2.59)** |
| **High-middle SDI** | **NA** | **NA** | **33085 (26377 to 39776)** | **1678 (1305 to 2104)** | **35.61 (28.39 to 42.82)** | **2.4 (1.86 to 3)** | **-8.82 (-9.01 to -8.63)** | **3124387 (2506993 to 3709927)** | **184219 (147038 to 222584)** | **3363.08 (2698.52 to 3993.35)** | **263 (209.92 to 317.77)** | **-8.28 (-8.43 to -8.14)** |
| **Middle SDI** | **NA** | **NA** | **288482 (222302 to 342110)** | **25439 (19432 to 33425)** | **143.86 (110.85 to 170.6)** | **14.4 (11 to 18.93)** | **-7.13 (-7.28 to -6.99)** | **26366427 (20552056 to 31139227)** | **2417638 (1881869 to 3098180)** | **13147.99 (10248.57 to 15528.01)** | **1368.86 (1065.51 to 1754.18)** | **-7.02 (-7.17 to -6.87)** |
| **Low-middle SDI** | **NA** | **NA** | **739331 (596347 to 863031)** | **85502 (64732 to 114557)** | **426.17 (343.75 to 497.47)** | **44.63 (33.79 to 59.8)** | **-6.87 (-7.16 to -6.57)** | **66632099 (53795719 to 77682088)** | **7843322 (5981529 to 10443268)** | **38408.45 (31009.23 to 44777.94)** | **4094.07 (3122.24 to 5451.19)** | **-6.81 (-7.1 to -6.51)** |
| **Low SDI** | **NA** | **NA** | **572316 (417109 to 717601)** | **227079 (160302 to 315667)** | **630.33 (459.39 to 790.35)** | **137.15 (96.81 to 190.65)** | **-4.73 (-5.02 to -4.43)** | **51316662 (37628480 to 64249579)** | **20382047 (14491345 to 28195005)** | **56518.92 (41443.09 to 70762.92)** | **12309.84 (8752.12 to 17028.51)** | **-4.72 (-5.01 to -4.42)** |
| **East Asia** | **0.722912** | **NA** | **72550 (53672 to 91813)** | **795 (602 to 1091)** | **62.68 (46.37 to 79.32)** | **0.99 (0.75 to 1.36)** | **-13.69 (-14.16 to -13.21)** | **6683248 (5027005 to 8392858)** | **90830 (70729 to 117457)** | **5773.92 (4343.03 to 7250.92)** | **113.43 (88.33 to 146.69)** | **-13.1 (-13.56 to -12.63)** |
| **China** | **0.718679** | **NA** | **72373 (53535 to 91645)** | **774 (576 to 1068)** | **64.73 (47.88 to 81.97)** | **1 (0.74 to 1.38)** | **-13.8 (-14.29 to -13.31)** | **6660942 (5004116 to 8371696)** | **84650 (65656 to 110304)** | **5957.59 (4475.71 to 7487.7)** | **108.99 (84.53 to 142.02)** | **-13.34 (-13.83 to -12.85)** |
| **Democratic People's Republic of Korea** | **0.569455** | **Low-middle SDI** | **117 (71 to 186)** | **17 (8 to 28)** | **5.03 (3.06 to 7.96)** | **1.15 (0.55 to 1.82)** | **-4.12 (-4.28 to -3.96)** | **14615 (10362 to 20910)** | **3721 (2608 to 5068)** | **625.55 (443.51 to 895.01)** | **245.94 (172.38 to 334.93)** | **-2.31 (-2.71 to -1.91)** |
| **Taiwan (Province of China)** | **0.87514** | **High SDI** | **59 (52 to 66)** | **3 (3 to 4)** | **3.69 (3.27 to 4.1)** | **0.37 (0.29 to 0.46)** | **-6.87 (-7.69 to -6.04)** | **7692 (6710 to 9034)** | **2458 (1641 to 3676)** | **478.79 (417.67 to 562.33)** | **275.49 (183.97 to 411.99)** | **-0.67 (-1.41 to 0.07)** |
| **Southeast Asia** | **0.649072** | **NA** | **170426 (106086 to 221428)** | **13698 (10285 to 17941)** | **292.38 (182 to 379.88)** | **24.34 (18.27 to 31.88)** | **-7.78 (-7.88 to -7.68)** | **15463620 (9786917 to 19970273)** | **1333684 (1019066 to 1709205)** | **26528.93 (16790.15 to 34260.42)** | **2369.54 (1810.56 to 3036.72)** | **-7.57 (-7.66 to -7.47)** |
| **Cambodia** | **0.474** | **Low-middle SDI** | **5443 (3755 to 7500)** | **284 (192 to 422)** | **298.26 (205.77 to 410.96)** | **16.22 (10.96 to 24.16)** | **-10.03 (-10.5 to -9.56)** | **494970 (345510 to 679255)** | **27410 (19126 to 39710)** | **27121 (18931.63 to 37218.59)** | **1567.6 (1093.81 to 2271.06)** | **-9.81 (-10.27 to -9.35)** |
| **Indonesia** | **0.657935** | **NA** | **108425 (69731 to 137441)** | **8261 (6106 to 10950)** | **485.76 (312.4 to 615.76)** | **37.71 (27.87 to 49.99)** | **-7.93 (-8.09 to -7.77)** | **9777390 (6372816 to 12364458)** | **793334 (598005 to 1029257)** | **43803.98 (28551.05 to 55394.38)** | **3621.45 (2729.8 to 4698.4)** | **-7.72 (-7.89 to -7.56)** |
| **Lao People's Democratic Republic** | **0.489281** | **Low-middle SDI** | **5338 (3340 to 7513)** | **384 (235 to 609)** | **750.82 (469.84 to 1056.71)** | **46.32 (28.37 to 73.47)** | **-8.89 (-9.19 to -8.58)** | **480067 (302228 to 673909)** | **35790 (22371 to 55987)** | **67524.18 (42510.12 to 94789.31)** | **4314.46 (2696.77 to 6749.26)** | **-8.79 (-9.09 to -8.49)** |
| **Malaysia** | **0.742553** | **High-middle SDI** | **377 (245 to 501)** | **76 (51 to 113)** | **15.84 (10.28 to 21.03)** | **3.09 (2.06 to 4.58)** | **-4.54 (-5.31 to -3.77)** | **37091 (25528 to 47833)** | **10382 (7471 to 13790)** | **1556.89 (1071.56 to 2007.83)** | **422.29 (303.9 to 560.93)** | **-3.49 (-4.21 to -2.77)** |
| **Maldives** | **0.657665** | **Middle SDI** | **133 (87 to 169)** | **3 (2 to 5)** | **319.07 (207.32 to 405.43)** | **10.89 (7.27 to 15.52)** | **-10.39 (-11.09 to -9.69)** | **12074 (7938 to 15305)** | **371 (269 to 508)** | **28898.35 (18999.67 to 36631.64)** | **1168.66 (847.8 to 1599.51)** | **-9.88 (-10.57 to -9.17)** |
| **Myanmar** | **0.528492** | **Low-middle SDI** | **26387 (12137 to 43405)** | **1759 (1149 to 2580)** | **523.32 (240.71 to 860.83)** | **33.66 (21.98 to 49.37)** | **-8.83 (-9.03 to -8.63)** | **2380070 (1115186 to 3892870)** | **168547 (112875 to 241670)** | **47202.82 (22116.97 to 77205.49)** | **3224.9 (2159.71 to 4624.01)** | **-8.67 (-8.86 to -8.47)** |
| **Philippines** | **0.65192** | **NA** | **16243 (11427 to 21237)** | **2484 (1833 to 3415)** | **175.58 (123.52 to 229.56)** | **22.15 (16.35 to 30.45)** | **-6.23 (-6.41 to -6.04)** | **1497037 (1068294 to 1942945)** | **240575 (183500 to 322311)** | **16182.17 (11547.69 to 21002.21)** | **2145.43 (1636.44 to 2874.35)** | **-6.11 (-6.28 to -5.94)** |
| **Sri Lanka** | **0.701372** | **Middle SDI** | **870 (636 to 1164)** | **46 (30 to 66)** | **49.18 (35.99 to 65.84)** | **2.93 (1.93 to 4.18)** | **-8.2 (-8.64 to -7.75)** | **81824 (60974 to 108162)** | **5835 (4247 to 7761)** | **4628.47 (3449.06 to 6118.3)** | **372.68 (271.3 to 495.74)** | **-7.4 (-7.81 to -6.97)** |
| **Thailand** | **0.682657** | **Middle SDI** | **2773 (994 to 4822)** | **208 (149 to 276)** | **53.33 (19.12 to 92.73)** | **7.36 (5.26 to 9.78)** | **-5.76 (-6.17 to -5.35)** | **266993 (109781 to 455278)** | **21673 (16186 to 27801)** | **5134.2 (2111.06 to 8754.89)** | **766.63 (572.55 to 983.4)** | **-5.55 (-5.91 to -5.19)** |
| **Timor-Leste** | **0.450689** | **Low SDI** | **915 (496 to 1262)** | **79 (45 to 125)** | **653.59 (354.12 to 901.09)** | **42.72 (24.15 to 67.56)** | **-9.21 (-9.6 to -8.81)** | **82319 (44981 to 113184)** | **7396 (4320 to 11476)** | **58778.37 (32117.66 to 80817.15)** | **4000.98 (2337.27 to 6208.19)** | **-9.03 (-9.41 to -8.66)** |
| **Viet Nam** | **0.621621** | **Middle SDI** | **3246 (1508 to 5276)** | **89 (42 to 144)** | **34.49 (16.03 to 56.07)** | **1.1 (0.52 to 1.76)** | **-10.68 (-10.93 to -10.43)** | **328374 (173592 to 506299)** | **19983 (13814 to 27122)** | **3489.87 (1844.89 to 5380.8)** | **245.43 (169.67 to 333.12)** | **-8.36 (-8.73 to -8)** |
| **Oceania** | **0.467359** | **NA** | **1920 (1337 to 2819)** | **1766 (1064 to 2754)** | **191.18 (133.17 to 280.73)** | **91.31 (55.02 to 142.36)** | **-1.82 (-2.05 to -1.59)** | **174375 (122706 to 254996)** | **161464 (99610 to 249857)** | **17364.81 (12219.49 to 25393.35)** | **8346.77 (5149.29 to 12916.15)** | **-1.82 (-2.04 to -1.6)** |
| **Fiji** | **0.669069** | **Middle SDI** | **32 (22 to 45)** | **16 (9 to 25)** | **33.73 (22.99 to 47.95)** | **17.54 (9.86 to 27.7)** | **-1.62 (-1.98 to -1.25)** | **3015 (2104 to 4187)** | **1557 (949 to 2361)** | **3199.26 (2233.07 to 4442.56)** | **1710.68 (1042.21 to 2593.45)** | **-1.55 (-1.89 to -1.2)** |
| **Kiribati** | **0.525958** | **Low-middle SDI** | **33 (24 to 45)** | **8 (5 to 12)** | **277.66 (200.92 to 384.43)** | **59.28 (36.76 to 86.27)** | **-4.65 (-4.86 to -4.43)** | **2951 (2151 to 4067)** | **769 (482 to 1112)** | **25062.61 (18265.53 to 34531.95)** | **5379.01 (3374.55 to 7782.63)** | **-4.64 (-4.85 to -4.42)** |
| **Marshall Islands** | **0.573525** | **Low-middle SDI** | **3 (2 to 5)** | **1 (0 to 1)** | **40.29 (22.71 to 63.38)** | **14.63 (8.51 to 22.63)** | **-3.17 (-3.99 to -2.34)** | **283 (164 to 437)** | **78 (47 to 119)** | **3798.86 (2199.63 to 5871.58)** | **1372.07 (832 to 2091.6)** | **-3.26 (-4.05 to -2.47)** |
| **Micronesia (Federated States of)** | **0.588013** | **Low-middle SDI** | **7 (5 to 11)** | **1 (1 to 1)** | **46.69 (30.57 to 68.37)** | **9.04 (5.35 to 14)** | **-4.91 (-6.13 to -3.68)** | **689 (460 to 991)** | **83 (51 to 125)** | **4397.28 (2940.63 to 6330.33)** | **873.92 (542.09 to 1320.53)** | **-4.97 (-6.1 to -3.83)** |
| **Papua New Guinea** | **0.418098** | **Low SDI** | **1645 (1107 to 2444)** | **1633 (973 to 2581)** | **253.96 (170.92 to 377.39)** | **107.35 (63.94 to 169.66)** | **-2.19 (-2.43 to -1.96)** | **149080 (101239 to 220587)** | **149068 (90587 to 233826)** | **23017.08 (15630.66 to 34057.3)** | **9798.66 (5954.52 to 15370.05)** | **-2.19 (-2.42 to -1.96)** |
| **Samoa** | **0.59234** | **Low-middle SDI** | **2 (1 to 4)** | **1 (0 to 2)** | **8.83 (3.26 to 14.39)** | **3.21 (1.38 to 5.27)** | **-3.04 (-3.37 to -2.71)** | **238 (112 to 359)** | **109 (61 to 161)** | **934.47 (439.46 to 1408.46)** | **373.65 (208.62 to 551.72)** | **-2.72 (-2.96 to -2.48)** |
| **Solomon Islands** | **0.429542** | **Low SDI** | **51 (32 to 77)** | **17 (11 to 24)** | **85.29 (54.31 to 128.78)** | **17.51 (11.25 to 25.47)** | **-4.57 (-4.77 to -4.37)** | **4680 (3025 to 6999)** | **1560 (1017 to 2216)** | **7845.02 (5071.05 to 11732.21)** | **1636.11 (1066.9 to 2324.46)** | **-4.54 (-4.73 to -4.35)** |
| **Tonga** | **0.629101** | **Middle SDI** | **2 (1 to 2)** | **1 (0 to 1)** | **10.96 (6.74 to 16.33)** | **3.57 (1.91 to 5.92)** | **-2.97 (-3.17 to -2.77)** | **168 (110 to 241)** | **56 (35 to 87)** | **1099.63 (719.54 to 1575.61)** | **389.63 (240.61 to 600.75)** | **-2.77 (-2.94 to -2.6)** |
| **Vanuatu** | **0.472796** | **Low-middle SDI** | **19 (11 to 31)** | **8 (4 to 15)** | **68.8 (39.05 to 115.62)** | **19.28 (9.91 to 36.07)** | **-3.88 (-4.11 to -3.64)** | **1727 (999 to 2847)** | **769 (417 to 1403)** | **6385.02 (3692.88 to 10530.35)** | **1824.19 (989.99 to 3327.82)** | **-3.83 (-4.05 to -3.61)** |
| **Central Asia** | **0.674963** | **NA** | **13675 (12328 to 15244)** | **1716 (1241 to 2307)** | **143.58 (129.43 to 160.05)** | **17.17 (12.41 to 23.07)** | **-7.69 (-7.99 to -7.39)** | **1246316 (1123608 to 1387721)** | **156035 (112985 to 208347)** | **13085.32 (11796.98 to 14569.95)** | **1560.82 (1130.19 to 2084.09)** | **-7.68 (-7.97 to -7.39)** |
| **Armenia** | **0.702497** | **Middle SDI** | **375 (317 to 439)** | **4 (3 to 5)** | **98.05 (82.91 to 114.74)** | **2.09 (1.57 to 2.78)** | **-14.08 (-14.94 to -13.21)** | **34519 (29268 to 40266)** | **405 (312 to 530)** | **9014.39 (7643.17 to 10515.29)** | **217.66 (167.32 to 284.63)** | **-13.5 (-14.24 to -12.74)** |
| **Azerbaijan** | **0.695411** | **Middle SDI** | **1493 (1140 to 1920)** | **170 (104 to 272)** | **165.22 (126.19 to 212.47)** | **23.52 (14.39 to 37.71)** | **-6.69 (-6.96 to -6.42)** | **135586 (104242 to 173875)** | **15485 (9601 to 24601)** | **15002.47 (11534.32 to 19239.14)** | **2147.51 (1331.53 to 3411.87)** | **-6.67 (-6.93 to -6.42)** |
| **Georgia** | **0.733124** | **High-middle SDI** | **197 (164 to 234)** | **2 (1 to 3)** | **41.97 (34.91 to 50.01)** | **0.86 (0.6 to 1.21)** | **-13.39 (-14.12 to -12.66)** | **18447 (15487 to 21824)** | **281 (214 to 372)** | **3937.03 (3305.2 to 4657.71)** | **115.6 (87.95 to 152.75)** | **-12.1 (-12.74 to -11.45)** |
| **Kazakhstan** | **0.718332** | **High-middle SDI** | **1640 (1436 to 1838)** | **29 (21 to 40)** | **87.09 (76.24 to 97.61)** | **1.51 (1.06 to 2.07)** | **-14.55 (-15.39 to -13.69)** | **150702 (132367 to 168668)** | **3110 (2304 to 4129)** | **8003.74 (7029.99 to 8957.89)** | **159.62 (118.23 to 211.93)** | **-14.08 (-14.88 to -13.29)** |
| **Kyrgyzstan** | **0.609181** | **Low-middle SDI** | **760 (650 to 871)** | **57 (44 to 72)** | **118.32 (101.11 to 135.55)** | **7.13 (5.57 to 9.03)** | **-9.67 (-10.39 to -8.94)** | **69452 (59364 to 79396)** | **5300 (4174 to 6643)** | **10805.6 (9236.07 to 12352.7)** | **667.63 (525.79 to 836.77)** | **-9.56 (-10.26 to -8.85)** |
| **Mongolia** | **0.618744** | **Low-middle SDI** | **206 (122 to 331)** | **23 (9 to 49)** | **60.76 (35.98 to 97.62)** | **5.83 (2.26 to 12.56)** | **-7.46 (-7.71 to -7.21)** | **19171 (11706 to 30379)** | **2099 (841 to 4456)** | **5646.51 (3447.88 to 8947.54)** | **537.28 (215.37 to 1140.54)** | **-7.45 (-7.74 to -7.16)** |
| **Tajikistan** | **0.536613** | **Low-middle SDI** | **3390 (2748 to 4166)** | **1307 (871 to 1832)** | **358.53 (290.62 to 440.59)** | **97.63 (65.07 to 136.88)** | **-4.77 (-5.1 to -4.44)** | **306899 (249120 to 376050)** | **117597 (78781 to 164559)** | **32457.53 (26346.9 to 39770.99)** | **8785.86 (5885.81 to 12294.45)** | **-4.78 (-5.11 to -4.45)** |
| **Turkmenistan** | **0.68304** | **Middle SDI** | **1573 (1358 to 1834)** | **43 (31 to 58)** | **268.55 (231.91 to 313.19)** | **8 (5.8 to 10.8)** | **-12.76 (-13.47 to -12.06)** | **142652 (123392 to 165888)** | **4008 (2977 to 5336)** | **24360.53 (21071.41 to 28328.49)** | **742.7 (551.59 to 988.7)** | **-12.68 (-13.37 to -11.98)** |
| **Uzbekistan** | **0.664965** | **Middle SDI** | **4041 (3549 to 4587)** | **82 (60 to 111)** | **119.8 (105.22 to 135.98)** | **2.13 (1.56 to 2.88)** | **-13.92 (-14.64 to -13.2)** | **368887 (325387 to 416644)** | **7749 (5831 to 10379)** | **10936.49 (9646.83 to 12352.33)** | **202.04 (152.03 to 270.63)** | **-13.78 (-14.48 to -13.08)** |
| **Central Europe** | **0.79578** | **NA** | **772 (687 to 868)** | **185 (151 to 218)** | **8.46 (7.53 to 9.51)** | **3.31 (2.7 to 3.9)** | **-2.65 (-3.82 to -1.47)** | **73196 (65724 to 81210)** | **17471 (14469 to 20434)** | **801.57 (719.75 to 889.34)** | **312.79 (259.04 to 365.82)** | **-2.66 (-3.68 to -1.63)** |
| **Albania** | **0.706889** | **Middle SDI** | **100 (73 to 136)** | **6 (3 to 9)** | **24.7 (18.02 to 33.59)** | **4.2 (2.22 to 6.25)** | **-6.02 (-6.74 to -5.3)** | **9179 (6759 to 12373)** | **566 (316 to 829)** | **2273.25 (1673.96 to 3064.34)** | **396.58 (221.76 to 581.32)** | **-5.91 (-6.54 to -5.27)** |
| **Bosnia and Herzegovina** | **0.722964** | **High-middle SDI** | **16 (11 to 22)** | **3 (2 to 5)** | **4.54 (3.16 to 6.16)** | **2.31 (1.55 to 3.38)** | **-1.55 (-1.91 to -1.2)** | **1647 (1205 to 2146)** | **359 (255 to 500)** | **468.39 (342.85 to 610.27)** | **237.43 (168.9 to 330.93)** | **-1.63 (-1.91 to -1.35)** |
| **Bulgaria** | **0.764641** | **High-middle SDI** | **23 (19 to 28)** | **12 (9 to 16)** | **4.22 (3.48 to 5.13)** | **3.99 (2.96 to 5.12)** | **-0.11 (-1.21 to 1)** | **2250 (1896 to 2678)** | **1135 (857 to 1437)** | **419.16 (353.16 to 498.83)** | **375.12 (283.25 to 474.79)** | **-0.26 (-1.26 to 0.74)** |
| **Croatia** | **0.799069** | **High-middle SDI** | **6 (5 to 7)** | **5 (3 to 6)** | **2.01 (1.65 to 2.46)** | **2.61 (1.86 to 3.42)** | **2.28 (0.34 to 4.26)** | **618 (509 to 738)** | **456 (334 to 586)** | **204.97 (169.09 to 245.12)** | **250.34 (183.3 to 321.73)** | **1.98 (0.31 to 3.69)** |
| **Czechia** | **0.82851** | **High SDI** | **34 (27 to 40)** | **30 (24 to 38)** | **5.17 (4.19 to 6.23)** | **5.39 (4.22 to 6.72)** | **1.6 (-0.31 to 3.54)** | **3133 (2568 to 3763)** | **2817 (2241 to 3478)** | **482.87 (395.76 to 580.04)** | **501.27 (398.8 to 618.86)** | **1.51 (-0.21 to 3.25)** |
| **Hungary** | **0.791025** | **High-middle SDI** | **30 (24 to 37)** | **30 (22 to 38)** | **4.8 (3.82 to 5.95)** | **6.59 (4.88 to 8.43)** | **2.51 (0.6 to 4.45)** | **3081 (2499 to 3723)** | **2797 (2092 to 3514)** | **497.57 (403.61 to 601.32)** | **615.59 (460.29 to 773.4)** | **1.85 (0.16 to 3.56)** |
| **North Macedonia** | **0.750955** | **High-middle SDI** | **126 (98 to 166)** | **7 (5 to 9)** | **74.09 (57.77 to 97.66)** | **6.52 (4.62 to 9.29)** | **-6.39 (-7.4 to -5.37)** | **11625 (9095 to 15245)** | **618 (448 to 874)** | **6824.59 (5339.34 to 8949.86)** | **614.35 (445.7 to 869.7)** | **-6.36 (-7.31 to -5.39)** |
| **Montenegro** | **0.796533** | **High-middle SDI** | **0 (0 to 1)** | **0 (0 to 0)** | **0.9 (0.6 to 1.4)** | **0.22 (0.11 to 0.41)** | **-4.48 (-4.91 to -4.06)** | **70 (52 to 95)** | **16 (10 to 23)** | **132.6 (98.65 to 180.93)** | **43.23 (28.61 to 63.11)** | **-2.97 (-3.52 to -2.41)** |
| **Poland** | **0.812073** | **NA** | **60 (53 to 67)** | **39 (31 to 49)** | **2.05 (1.8 to 2.31)** | **2.09 (1.62 to 2.61)** | **2.35 (0.11 to 4.63)** | **6531 (5693 to 7433)** | **3630 (2830 to 4511)** | **223.75 (195.04 to 254.66)** | **192.86 (150.35 to 239.66)** | **1.35 (-0.38 to 3.11)** |
| **Romania** | **0.766321** | **High-middle SDI** | **320 (269 to 374)** | **39 (29 to 49)** | **18.06 (15.21 to 21.12)** | **4.12 (3.09 to 5.23)** | **-4.92 (-5.82 to -4.02)** | **29401 (24910 to 34221)** | **3635 (2737 to 4582)** | **1661.53 (1407.72 to 1933.89)** | **387.56 (291.83 to 488.57)** | **-4.83 (-5.66 to -4)** |
| **Serbia** | **0.792213** | **High-middle SDI** | **36 (15 to 56)** | **5 (3 to 8)** | **5.32 (2.17 to 8.26)** | **1.42 (0.91 to 2.21)** | **-4.14 (-4.8 to -3.48)** | **3498 (1586 to 5229)** | **567 (389 to 832)** | **513.91 (233.02 to 768.12)** | **153.69 (105.63 to 225.66)** | **-3.42 (-3.79 to -3.04)** |
| **Slovakia** | **0.808329** | **High-middle SDI** | **9 (6 to 12)** | **5 (3 to 9)** | **2.17 (1.54 to 2.86)** | **1.89 (1.13 to 3.2)** | **0.48 (-0.09 to 1.04)** | **859 (624 to 1111)** | **521 (326 to 859)** | **209.88 (152.49 to 271.55)** | **181.96 (113.77 to 300.35)** | **0.41 (-0.1 to 0.92)** |
| **Slovenia** | **0.842633** | **High SDI** | **1 (1 to 1)** | **1 (1 to 1)** | **0.83 (0.66 to 1.02)** | **0.74 (0.53 to 1)** | **0.87 (-0.9 to 2.66)** | **134 (108 to 168)** | **101 (78 to 132)** | **109.03 (88.14 to 136.5)** | **103.65 (79.33 to 134.86)** | **0.78 (0.02 to 1.54)** |
| **Eastern Europe** | **0.803414** | **NA** | **1386 (1315 to 1462)** | **91 (81 to 101)** | **8.04 (7.62 to 8.48)** | **0.9 (0.8 to 1)** | **-8.27 (-8.88 to -7.65)** | **161898 (147089 to 179379)** | **12682 (10960 to 14855)** | **938.96 (853.08 to 1040.35)** | **125.33 (108.31 to 146.8)** | **-7.2 (-7.69 to -6.7)** |
| **Belarus** | **0.784114** | **High-middle SDI** | **35 (28 to 46)** | **2 (1 to 3)** | **4.36 (3.46 to 5.66)** | **0.41 (0.28 to 0.59)** | **-9.46 (-10.41 to -8.51)** | **4433 (3679 to 5488)** | **530 (367 to 712)** | **550.16 (456.6 to 681.07)** | **113.44 (78.61 to 152.42)** | **-6.38 (-6.97 to -5.78)** |
| **Estonia** | **0.845787** | **High SDI** | **5 (4 to 5)** | **0 (0 to 0)** | **3.91 (3.38 to 4.5)** | **0.25 (0.19 to 0.33)** | **-10.06 (-10.79 to -9.33)** | **573 (487 to 674)** | **84 (58 to 116)** | **474.73 (403.96 to 558.45)** | **122.12 (84.26 to 167.89)** | **-4.64 (-4.89 to -4.39)** |
| **Latvia** | **0.830715** | **High SDI** | **7 (5 to 8)** | **0 (0 to 0)** | **3.26 (2.71 to 3.99)** | **0.24 (0.18 to 0.34)** | **-9.75 (-10.42 to -9.09)** | **847 (715 to 1018)** | **81 (57 to 109)** | **418.02 (352.64 to 502.4)** | **86.96 (61.09 to 115.98)** | **-5.75 (-6.11 to -5.39)** |
| **Lithuania** | **0.857613** | **High SDI** | **14 (12 to 17)** | **1 (1 to 1)** | **4.85 (4.07 to 5.74)** | **0.53 (0.4 to 0.71)** | **-8.28 (-8.96 to -7.6)** | **1628 (1362 to 1929)** | **176 (132 to 229)** | **563.09 (471.11 to 667.37)** | **133.8 (100.4 to 174.38)** | **-5.52 (-6.05 to -4.99)** |
| **Republic of Moldova** | **0.732393** | **High-middle SDI** | **93 (76 to 112)** | **3 (2 to 5)** | **21.55 (17.67 to 26.02)** | **2.21 (1.51 to 3.23)** | **-8.04 (-8.75 to -7.32)** | **9268 (7668 to 11023)** | **418 (313 to 552)** | **2150.99 (1779.71 to 2558.45)** | **271.05 (202.95 to 357.92)** | **-7.1 (-7.57 to -6.64)** |
| **Russian Federation** | **0.809111** | **NA** | **1054 (1004 to 1109)** | **73 (64 to 83)** | **9.07 (8.64 to 9.54)** | **0.97 (0.84 to 1.08)** | **-8.3 (-8.96 to -7.64)** | **122667 (111793 to 135072)** | **9777 (8461 to 11566)** | **1055.27 (961.72 to 1161.98)** | **128.47 (111.17 to 151.97)** | **-7.34 (-7.92 to -6.76)** |
| **Ukraine** | **0.761046** | **NA** | **179 (154 to 209)** | **11 (9 to 15)** | **4.74 (4.07 to 5.53)** | **0.71 (0.56 to 0.94)** | **-8.3 (-9.21 to -7.38)** | **22483 (19247 to 26317)** | **1615 (1305 to 2007)** | **596.52 (510.66 to 698.27)** | **101.41 (81.98 to 126.08)** | **-7.37 (-8.01 to -6.72)** |
| **High-income Asia Pacific** | **0.877157** | **NA** | **173 (144 to 217)** | **43 (37 to 49)** | **1.69 (1.41 to 2.12)** | **0.66 (0.58 to 0.75)** | **-2.19 (-2.55 to -1.83)** | **27819 (22284 to 35487)** | **13321 (9777 to 18648)** | **272.31 (218.13 to 347.37)** | **206.46 (151.52 to 289.02)** | **-0.22 (-0.58 to 0.14)** |
| **Brunei Darussalam** | **0.810289** | **High-middle SDI** | **0 (0 to 1)** | **0 (0 to 1)** | **1.35 (0.92 to 1.87)** | **1.21 (0.8 to 1.71)** | **0.33 (0.07 to 0.58)** | **50 (36 to 67)** | **38 (27 to 53)** | **144.76 (103.75 to 192.92)** | **122.88 (85.79 to 171.08)** | **0.04 (-0.18 to 0.25)** |
| **Japan** | **0.87146** | **NA** | **66 (61 to 70)** | **30 (25 to 34)** | **0.99 (0.92 to 1.05)** | **0.65 (0.55 to 0.75)** | **-0.7 (-1.32 to -0.07)** | **17010 (12599 to 23158)** | **11700 (8369 to 16688)** | **255.47 (189.23 to 347.81)** | **255.15 (182.51 to 363.94)** | **0.56 (0.13 to 1)** |
| **Republic of Korea** | **0.887196** | **High SDI** | **102 (73 to 145)** | **11 (7 to 17)** | **3.09 (2.21 to 4.38)** | **0.74 (0.48 to 1.1)** | **-3.99 (-4.31 to -3.67)** | **10334 (7749 to 14228)** | **1449 (1006 to 2039)** | **311.34 (233.48 to 428.68)** | **93.48 (64.92 to 131.58)** | **-3.23 (-3.47 to -2.99)** |
| **Singapore** | **0.856235** | **High SDI** | **4 (4 to 5)** | **1 (1 to 2)** | **2.13 (1.84 to 2.46)** | **0.41 (0.32 to 0.53)** | **-3.43 (-4.16 to -2.69)** | **425 (373 to 485)** | **134 (107 to 169)** | **208.48 (182.72 to 237.87)** | **46.97 (37.38 to 58.96)** | **-3.14 (-3.8 to -2.48)** |
| **Australasia** | **0.845644** | **NA** | **14 (12 to 16)** | **6 (5 to 7)** | **0.89 (0.78 to 1.01)** | **0.33 (0.27 to 0.41)** | **-0.39 (-1.58 to 0.8)** | **2118 (1731 to 2624)** | **902 (735 to 1133)** | **137.34 (112.21 to 170.14)** | **49.68 (40.5 to 62.37)** | **-0.89 (-1.69 to -0.09)** |
| **Australia** | **0.844269** | **High SDI** | **10 (9 to 12)** | **3 (3 to 4)** | **0.81 (0.7 to 0.93)** | **0.23 (0.17 to 0.29)** | **-1.54 (-2.57 to -0.5)** | **1584 (1284 to 1998)** | **514 (404 to 665)** | **125.47 (101.69 to 158.22)** | **34.19 (26.88 to 44.2)** | **-1.65 (-2.49 to -0.79)** |
| **New Zealand** | **0.850145** | **NA** | **3 (3 to 4)** | **3 (2 to 3)** | **1.25 (1.08 to 1.43)** | **0.84 (0.68 to 1.02)** | **2.54 (0.65 to 4.46)** | **534 (438 to 670)** | **388 (318 to 480)** | **190.95 (156.74 to 239.44)** | **124.16 (101.86 to 153.45)** | **0.77 (-0.15 to 1.69)** |
| **Western Europe** | **0.848729** | **NA** | **168 (154 to 183)** | **134 (113 to 155)** | **0.73 (0.67 to 0.8)** | **0.63 (0.53 to 0.73)** | **0.65 (-0.19 to 1.5)** | **48915 (35765 to 68693)** | **39636 (29568 to 54280)** | **213.08 (155.79 to 299.23)** | **186.7 (139.28 to 255.69)** | **0.14 (-0.5 to 0.78)** |
| **Andorra** | **0.869895** | **High SDI** | **0 (0 to 0)** | **0 (0 to 0)** | **0.16 (0.09 to 0.25)** | **0.04 (0.02 to 0.06)** | **-3.92 (-4.25 to -3.59)** | **5 (3 to 8)** | **5 (3 to 7)** | **184.95 (115.38 to 280.63)** | **181.86 (107.28 to 287.29)** | **0.35 (-0.18 to 0.88)** |
| **Austria** | **0.854558** | **High SDI** | **2 (2 to 2)** | **1 (1 to 1)** | **0.45 (0.39 to 0.52)** | **0.21 (0.17 to 0.26)** | **0.03 (-1.05 to 1.12)** | **1645 (1092 to 2393)** | **665 (464 to 964)** | **368.03 (244.38 to 535.41)** | **153.9 (107.43 to 223.02)** | **-3.09 (-4.02 to -2.14)** |
| **Belgium** | **0.853674** | **High SDI** | **9 (7 to 10)** | **10 (7 to 12)** | **1.44 (1.22 to 1.7)** | **1.64 (1.26 to 2.11)** | **1.57 (0.71 to 2.44)** | **1559 (1207 to 2032)** | **1444 (1133 to 1841)** | **261.28 (202.42 to 340.71)** | **243.9 (191.41 to 311.04)** | **0.34 (-0.31 to 0.98)** |
| **Cyprus** | **0.835649** | **High SDI** | **3 (1 to 5)** | **1 (0 to 1)** | **4.88 (2.28 to 7.16)** | **0.93 (0.59 to 1.43)** | **-5.24 (-5.41 to -5.08)** | **376 (232 to 509)** | **197 (137 to 278)** | **588.29 (363.3 to 797.23)** | **262.86 (182.76 to 370.36)** | **-2 (-2.28 to -1.73)** |
| **Denmark** | **0.897314** | **High SDI** | **4 (4 to 5)** | **4 (3 to 5)** | **1.45 (1.27 to 1.67)** | **1.21 (0.93 to 1.49)** | **0.89 (0.11 to 1.69)** | **822 (642 to 1075)** | **904 (670 to 1224)** | **283.7 (221.64 to 371.16)** | **291.09 (215.68 to 393.96)** | **1.06 (0.49 to 1.63)** |
| **Finland** | **0.860244** | **High SDI** | **1 (1 to 1)** | **1 (1 to 1)** | **0.32 (0.27 to 0.37)** | **0.35 (0.28 to 0.44)** | **1.58 (0.58 to 2.6)** | **597 (390 to 885)** | **344 (231 to 490)** | **190.8 (124.88 to 283.13)** | **141.73 (95.13 to 201.98)** | **-0.7 (-1.5 to 0.09)** |
| **France** | **0.837816** | **High SDI** | **55 (48 to 63)** | **29 (23 to 36)** | **1.42 (1.24 to 1.62)** | **0.82 (0.65 to 1)** | **-1.71 (-2.22 to -1.19)** | **11364 (8809 to 14827)** | **10968 (7708 to 15686)** | **292.18 (226.5 to 381.23)** | **310.36 (218.12 to 443.87)** | **0.49 (0.17 to 0.81)** |
| **Germany** | **0.903516** | **High SDI** | **35 (29 to 41)** | **26 (21 to 33)** | **0.78 (0.66 to 0.92)** | **0.65 (0.52 to 0.81)** | **1.24 (0.35 to 2.14)** | **10320 (7277 to 14884)** | **6380 (4891 to 8544)** | **230.4 (162.45 to 332.29)** | **157.79 (120.97 to 211.31)** | **-0.46 (-1.52 to 0.62)** |
| **Greece** | **0.791882** | **High-middle SDI** | **0 (0 to 1)** | **1 (0 to 1)** | **0.08 (0.07 to 0.09)** | **0.14 (0.11 to 0.18)** | **3.41 (2.75 to 4.07)** | **1025 (640 to 1597)** | **624 (403 to 949)** | **184.76 (115.34 to 287.75)** | **147.54 (95.28 to 224.63)** | **0.02 (-0.63 to 0.68)** |
| **Iceland** | **0.874629** | **High SDI** | **0 (0 to 0)** | **0 (0 to 0)** | **0.31 (0.26 to 0.38)** | **0.34 (0.25 to 0.44)** | **1.49 (0.57 to 2.41)** | **41 (26 to 60)** | **45 (29 to 67)** | **193.34 (125.09 to 283.92)** | **203.74 (133.82 to 302.85)** | **0.68 (0.09 to 1.28)** |
| **Ireland** | **0.87399** | **High SDI** | **1 (1 to 1)** | **1 (1 to 1)** | **0.25 (0.22 to 0.29)** | **0.23 (0.18 to 0.3)** | **0.93 (-0.18 to 2.04)** | **501 (318 to 770)** | **549 (358 to 828)** | **173.48 (110.08 to 266.31)** | **184.01 (120.07 to 277.42)** | **1.94 (1.04 to 2.86)** |
| **Israel** | **0.809091** | **High-middle SDI** | **9 (8 to 11)** | **11 (8 to 13)** | **1.77 (1.51 to 2.07)** | **1.16 (0.91 to 1.45)** | **0.56 (-0.32 to 1.45)** | **1483 (1201 to 1882)** | **1794 (1407 to 2305)** | **287.21 (232.55 to 364.54)** | **195.37 (153.26 to 250.97)** | **-0.01 (-0.8 to 0.78)** |
| **Italy** | **0.805537** | **NA** | **8 (7 to 9)** | **16 (13 to 20)** | **0.29 (0.27 to 0.31)** | **0.75 (0.59 to 0.94)** | **6.79 (4.94 to 8.67)** | **3144 (2179 to 4544)** | **2669 (2110 to 3339)** | **114.5 (79.35 to 165.48)** | **123.01 (97.24 to 153.87)** | **1.15 (0.82 to 1.49)** |
| **Luxembourg** | **0.884636** | **High SDI** | **0 (0 to 0)** | **0 (0 to 0)** | **0.96 (0.82 to 1.13)** | **0.9 (0.7 to 1.16)** | **0.89 (0.22 to 1.56)** | **51 (39 to 69)** | **62 (47 to 81)** | **224.14 (171.69 to 300.49)** | **187.02 (142.66 to 244.24)** | **-0.13 (-0.67 to 0.42)** |
| **Malta** | **0.801854** | **High-middle SDI** | **0 (0 to 0)** | **0 (0 to 0)** | **0.18 (0.14 to 0.21)** | **0.3 (0.22 to 0.4)** | **3.45 (2.53 to 4.38)** | **48 (30 to 74)** | **40 (26 to 61)** | **169.29 (104.63 to 261.42)** | **181.74 (120.21 to 275.06)** | **0.75 (0.2 to 1.29)** |
| **Netherlands** | **0.888376** | **High SDI** | **4 (4 to 5)** | **6 (5 to 7)** | **0.47 (0.41 to 0.53)** | **0.68 (0.55 to 0.81)** | **2.43 (1.68 to 3.19)** | **2205 (1477 to 3229)** | **3159 (2077 to 4689)** | **235.48 (157.75 to 344.88)** | **366.75 (241.12 to 544.41)** | **2.09 (1.47 to 2.71)** |
| **Norway** | **0.916632** | **NA** | **0 (0 to 0)** | **1 (0 to 1)** | **0.14 (0.12 to 0.16)** | **0.2 (0.16 to 0.24)** | **3.2 (1.89 to 4.52)** | **301 (194 to 451)** | **287 (195 to 420)** | **109.1 (70.3 to 163.66)** | **102.09 (69.52 to 149.34)** | **-0.21 (-0.49 to 0.07)** |
| **Portugal** | **0.745395** | **High-middle SDI** | **12 (11 to 14)** | **2 (1 to 2)** | **2.09 (1.84 to 2.43)** | **0.44 (0.34 to 0.54)** | **-4.32 (-5.04 to -3.59)** | **1969 (1608 to 2452)** | **557 (413 to 754)** | **340.33 (278.06 to 423.97)** | **130.9 (97.2 to 177.17)** | **-2.22 (-2.53 to -1.91)** |
| **Spain** | **0.769483** | **High-middle SDI** | **14 (12 to 15)** | **10 (8 to 12)** | **0.65 (0.57 to 0.73)** | **0.56 (0.45 to 0.66)** | **0.93 (0.05 to 1.82)** | **4349 (3074 to 5939)** | **3639 (2620 to 4954)** | **208.82 (147.6 to 285.16)** | **197.63 (142.32 to 269.03)** | **0.5 (0.09 to 0.91)** |
| **Sweden** | **0.887384** | **NA** | **0 (0 to 0)** | **2 (2 to 3)** | **0.06 (0.06 to 0.07)** | **0.4 (0.32 to 0.48)** | **8.33 (7.02 to 9.65)** | **558 (349 to 854)** | **726 (520 to 1004)** | **99.15 (61.97 to 151.72)** | **124.48 (89.13 to 172.09)** | **1.62 (1.32 to 1.92)** |
| **Switzerland** | **0.933532** | **High SDI** | **3 (3 to 4)** | **3 (2 to 3)** | **0.84 (0.72 to 0.95)** | **0.6 (0.46 to 0.74)** | **0.27 (-0.5 to 1.06)** | **1211 (881 to 1660)** | **1266 (890 to 1735)** | **304.11 (221.15 to 416.73)** | **286.39 (201.4 to 392.55)** | **0.32 (-0.13 to 0.78)** |
| **United Kingdom** | **0.858445** | **NA** | **7 (6 to 7)** | **10 (9 to 12)** | **0.17 (0.16 to 0.18)** | **0.28 (0.23 to 0.32)** | **1.66 (-1.17 to 4.57)** | **5297 (3378 to 8028)** | **3273 (2315 to 4596)** | **137.89 (87.95 to 208.99)** | **89.57 (63.34 to 125.76)** | **-0.84 (-2.25 to 0.6)** |
| **Southern Latin America** | **0.74303** | **NA** | **860 (790 to 937)** | **89 (70 to 113)** | **16.71 (15.34 to 18.2)** | **2.07 (1.63 to 2.64)** | **-5.81 (-6.15 to -5.46)** | **86979 (79550 to 94439)** | **9692 (7797 to 12151)** | **1689.92 (1545.58 to 1834.86)** | **226.52 (182.24 to 284.02)** | **-5.44 (-5.79 to -5.08)** |
| **Argentina** | **0.733528** | **High-middle SDI** | **646 (580 to 708)** | **64 (49 to 83)** | **18.79 (16.88 to 20.6)** | **2.14 (1.64 to 2.77)** | **-6.11 (-6.46 to -5.75)** | **65598 (59333 to 71892)** | **6965 (5506 to 8762)** | **1908.68 (1726.4 to 2091.84)** | **232.47 (183.75 to 292.43)** | **-5.69 (-6.11 to -5.27)** |
| **Chile** | **0.770149** | **High-middle SDI** | **161 (145 to 178)** | **18 (15 to 22)** | **11.24 (10.08 to 12.36)** | **1.65 (1.33 to 2.06)** | **-4.99 (-5.58 to -4.39)** | **16167 (14593 to 17930)** | **2071 (1681 to 2528)** | **1125.03 (1015.49 to 1247.71)** | **190.32 (154.55 to 232.33)** | **-4.75 (-5.02 to -4.48)** |
| **Uruguay** | **0.721713** | **High-middle SDI** | **53 (47 to 60)** | **7 (5 to 9)** | **19.43 (17.25 to 22)** | **3.4 (2.56 to 4.4)** | **-5.34 (-5.69 to -5)** | **5210 (4631 to 5804)** | **655 (500 to 831)** | **1909.47 (1697.5 to 2127.23)** | **337.77 (257.8 to 428.55)** | **-5.33 (-5.63 to -5.03)** |
| **High-income North America** | **0.864217** | **NA** | **224 (211 to 238)** | **116 (101 to 132)** | **1.03 (0.97 to 1.1)** | **0.57 (0.49 to 0.65)** | **-2.03 (-2.6 to -1.46)** | **34274 (28625 to 42552)** | **11656 (10141 to 13266)** | **158.08 (132.02 to 196.26)** | **56.86 (49.47 to 64.72)** | **-3.07 (-3.58 to -2.55)** |
| **Canada** | **0.873182** | **High SDI** | **7 (6 to 8)** | **18 (14 to 22)** | **0.37 (0.31 to 0.43)** | **0.93 (0.72 to 1.16)** | **5.44 (3.76 to 7.16)** | **2320 (1617 to 3311)** | **1948 (1544 to 2432)** | **120.49 (84.02 to 171.98)** | **102.51 (81.25 to 128.02)** | **0.74 (-0.34 to 1.82)** |
| **United States of America** | **0.863244** | **NA** | **217 (204 to 231)** | **98 (86 to 113)** | **1.1 (1.03 to 1.17)** | **0.53 (0.46 to 0.61)** | **-2.7 (-3.26 to -2.13)** | **31935 (26903 to 39162)** | **9703 (8508 to 11036)** | **161.69 (136.22 to 198.28)** | **52.18 (45.76 to 59.35)** | **-3.64 (-4.15 to -3.11)** |
| **Caribbean** | **0.642315** | **NA** | **12534 (10280 to 14893)** | **3974 (2659 to 5528)** | **303.39 (248.83 to 360.48)** | **102.75 (68.75 to 142.91)** | **-3.13 (-3.61 to -2.65)** | **1125813 (926617 to 1335056)** | **356778 (240071 to 495586)** | **27250.03 (22428.55 to 32314.72)** | **9223.41 (6206.31 to 12811.86)** | **-3.13 (-3.61 to -2.65)** |
| **Antigua and Barbuda** | **0.74985** | **High-middle SDI** | **1 (1 to 1)** | **0 (0 to 0)** | **11.1 (8.88 to 13.7)** | **3.33 (2.61 to 4.1)** | **-3.83 (-4.34 to -3.32)** | **65 (53 to 80)** | **18 (15 to 22)** | **1071.04 (874.92 to 1312.73)** | **341.29 (276.05 to 408.89)** | **-3.66 (-4.1 to -3.22)** |
| **Bahamas** | **0.805144** | **High-middle SDI** | **4 (3 to 5)** | **1 (0 to 1)** | **14.45 (11.38 to 18.17)** | **2.45 (1.72 to 3.4)** | **-5.81 (-6.53 to -5.09)** | **345 (273 to 432)** | **52 (38 to 69)** | **1353.69 (1072.01 to 1692.28)** | **244.82 (180.42 to 329.38)** | **-5.53 (-6.16 to -4.89)** |
| **Barbados** | **0.747065** | **High-middle SDI** | **2 (1 to 2)** | **0 (0 to 0)** | **9.18 (7.4 to 11)** | **1.89 (1.25 to 2.74)** | **-5.16 (-5.74 to -4.56)** | **176 (143 to 209)** | **30 (22 to 41)** | **899.8 (728.13 to 1069.74)** | **222.74 (162.7 to 301.32)** | **-4.44 (-4.91 to -3.96)** |
| **Belize** | **0.610552** | **Low-middle SDI** | **27 (23 to 31)** | **4 (3 to 4)** | **89.98 (77.71 to 103.55)** | **9.22 (7.02 to 11.56)** | **-7.12 (-7.66 to -6.58)** | **2417 (2096 to 2777)** | **330 (256 to 410)** | **8180.73 (7096.63 to 9401.58)** | **864.93 (672.16 to 1074.54)** | **-7 (-7.53 to -6.47)** |
| **Cuba** | **0.669332** | **Middle SDI** | **138 (125 to 152)** | **11 (9 to 14)** | **15.43 (13.92 to 17.04)** | **2.06 (1.57 to 2.59)** | **-5.89 (-6.66 to -5.11)** | **12985 (11709 to 14303)** | **1312 (1050 to 1620)** | **1450.75 (1308.17 to 1597.95)** | **240.9 (192.92 to 297.47)** | **-5.11 (-5.79 to -4.42)** |
| **Dominica** | **0.747382** | **High-middle SDI** | **1 (1 to 2)** | **0 (0 to 1)** | **16.5 (11.68 to 22.53)** | **12.14 (7.1 to 18.89)** | **-0.75 (-1.33 to -0.16)** | **133 (95 to 179)** | **39 (23 to 60)** | **1534.66 (1096.89 to 2068.14)** | **1117.92 (667.37 to 1720.93)** | **-0.79 (-1.34 to -0.24)** |
| **Dominican Republic** | **0.619171** | **Middle SDI** | **2146 (1689 to 2742)** | **256 (133 to 379)** | **215.48 (169.65 to 275.32)** | **24.78 (12.84 to 36.72)** | **-6.66 (-7.09 to -6.22)** | **194770 (153574 to 248170)** | **23343 (12397 to 34301)** | **19558.16 (15421.38 to 24920.43)** | **2259.47 (1199.92 to 3320.17)** | **-6.62 (-7.03 to -6.21)** |
| **Grenada** | **0.669351** | **Middle SDI** | **2 (1 to 2)** | **0 (0 to 0)** | **15.16 (12.07 to 19.35)** | **2.39 (1.77 to 3.11)** | **-5.4 (-6.17 to -4.63)** | **171 (137 to 216)** | **18 (14 to 23)** | **1427.89 (1142.46 to 1801.91)** | **260.32 (201.85 to 330.31)** | **-4.99 (-5.65 to -4.33)** |
| **Guyana** | **0.650902** | **Middle SDI** | **176 (142 to 213)** | **14 (10 to 20)** | **156.17 (125.85 to 188.62)** | **19.11 (13.47 to 26.4)** | **-6.49 (-6.79 to -6.18)** | **15862 (12797 to 19133)** | **1287 (914 to 1774)** | **14061.69 (11344.43 to 16961.56)** | **1727.72 (1226.64 to 2381.48)** | **-6.48 (-6.79 to -6.17)** |
| **Haiti** | **0.448751** | **Low SDI** | **9344 (7327 to 11297)** | **3525 (2331 to 4963)** | **880.89 (690.79 to 1065.03)** | **224.53 (148.46 to 316.16)** | **-4.12 (-4.61 to -3.63)** | **835577 (656153 to 1009712)** | **315525 (208624 to 443517)** | **78775.87 (61860.31 to 95192.84)** | **20099.09 (13289.45 to 28252.23)** | **-4.12 (-4.6 to -3.63)** |
| **Jamaica** | **0.683064** | **Middle SDI** | **152 (130 to 179)** | **9 (6 to 13)** | **54.59 (46.45 to 64.23)** | **5.33 (3.67 to 7.45)** | **-6.01 (-7.45 to -4.56)** | **14165 (12105 to 16602)** | **875 (617 to 1206)** | **5077.07 (4338.72 to 5950.38)** | **510.97 (360.25 to 704.28)** | **-5.97 (-7.33 to -4.6)** |
| **Saint Lucia** | **0.672602** | **Middle SDI** | **5 (4 to 6)** | **0 (0 to 1)** | **29.31 (23.67 to 35.98)** | **4.4 (3.06 to 6.42)** | **-5.67 (-6.45 to -4.87)** | **479 (387 to 586)** | **39 (28 to 56)** | **2709.93 (2192.73 to 3319.02)** | **444.37 (319.81 to 629.94)** | **-5.38 (-6.11 to -4.64)** |
| **Saint Vincent and the Grenadines** | **0.640887** | **Middle SDI** | **7 (6 to 9)** | **0 (0 to 1)** | **57.37 (44.44 to 71.77)** | **6.32 (4.59 to 8.74)** | **-6.63 (-7.26 to -6)** | **671 (525 to 834)** | **43 (32 to 58)** | **5252.25 (4110.89 to 6527.34)** | **601.34 (446.59 to 811.14)** | **-6.53 (-7.14 to -5.92)** |
| **Suriname** | **0.641163** | **Middle SDI** | **58 (42 to 76)** | **13 (8 to 18)** | **131.69 (96.4 to 172.98)** | **28.37 (17.6 to 41.07)** | **-5.18 (-5.44 to -4.93)** | **5231 (3843 to 6845)** | **1146 (718 to 1652)** | **11941.84 (8773.02 to 15627.38)** | **2573.36 (1611.21 to 3709.52)** | **-5.18 (-5.43 to -4.93)** |
| **Trinidad and Tobago** | **0.769401** | **High-middle SDI** | **30 (24 to 36)** | **3 (2 to 5)** | **22.34 (17.66 to 27.33)** | **4.29 (3.03 to 6.11)** | **-5.85 (-6.21 to -5.5)** | **2755 (2196 to 3356)** | **335 (243 to 468)** | **2066.27 (1647.33 to 2517.41)** | **415.62 (301.77 to 580.86)** | **-5.65 (-5.98 to -5.32)** |
| **Andean Latin America** | **0.654008** | **NA** | **7873 (6580 to 9425)** | **564 (368 to 799)** | **149.07 (124.59 to 178.45)** | **9.16 (5.99 to 12.99)** | **-9.06 (-9.31 to -8.8)** | **733006 (616412 to 866680)** | **53206 (36050 to 74222)** | **13878.76 (11671.16 to 16409.75)** | **864.33 (585.62 to 1205.73)** | **-8.99 (-9.21 to -8.77)** |
| **Bolivia (Plurinational State of)** | **0.604497** | **Low-middle SDI** | **2254 (1480 to 3253)** | **285 (170 to 419)** | **223.15 (146.49 to 322.12)** | **23.9 (14.24 to 35.1)** | **-7.39 (-7.53 to -7.26)** | **205459 (136862 to 294454)** | **25863 (15602 to 37705)** | **20343.48 (13551.42 to 29155.3)** | **2165.7 (1306.49 to 3157.42)** | **-7.41 (-7.54 to -7.28)** |
| **Ecuador** | **0.665675** | **Middle SDI** | **1784 (1634 to 1957)** | **60 (42 to 86)** | **132.59 (121.47 to 145.45)** | **3.61 (2.53 to 5.18)** | **-11.52 (-12.03 to -11)** | **167865 (153954 to 183025)** | **5902 (4278 to 8149)** | **12475.4 (11441.59 to 13602.11)** | **355.29 (257.55 to 490.59)** | **-11.42 (-11.94 to -10.89)** |
| **Peru** | **0.662036** | **Middle SDI** | **3835 (3024 to 4838)** | **218 (124 to 337)** | **131.07 (103.36 to 165.35)** | **6.62 (3.75 to 10.22)** | **-9.69 (-10.15 to -9.22)** | **359682 (285405 to 449603)** | **21442 (12965 to 32213)** | **12292.72 (9754.18 to 15365.91)** | **649.66 (392.82 to 976.01)** | **-9.44 (-9.81 to -9.07)** |
| **Central Latin America** | **0.641931** | **NA** | **39758 (36665 to 43414)** | **2903 (2109 to 3872)** | **172.73 (159.29 to 188.61)** | **14.45 (10.5 to 19.28)** | **-7.62 (-8.05 to -7.2)** | **3601608 (3324906 to 3932793)** | **269557 (198644 to 357488)** | **15646.98 (14444.86 to 17085.79)** | **1341.72 (988.75 to 1779.4)** | **-7.56 (-7.97 to -7.14)** |
| **Colombia** | **0.65664** | **Middle SDI** | **3436 (2942 to 3902)** | **214 (142 to 316)** | **81.67 (69.94 to 92.74)** | **6.22 (4.14 to 9.19)** | **-9.02 (-9.73 to -8.31)** | **316454 (271669 to 360332)** | **21295 (14745 to 30251)** | **7522.21 (6457.64 to 8565.19)** | **618.55 (428.29 to 878.69)** | **-8.76 (-9.42 to -8.1)** |
| **Costa Rica** | **0.70437** | **Middle SDI** | **88 (77 to 99)** | **8 (6 to 10)** | **21.68 (18.87 to 24.45)** | **2.5 (1.93 to 3.12)** | **-7.12 (-7.78 to -6.45)** | **8473 (7399 to 9475)** | **949 (761 to 1175)** | **2087.14 (1822.67 to 2333.8)** | **307.74 (246.8 to 380.97)** | **-6.18 (-6.74 to -5.61)** |
| **El Salvador** | **0.56557** | **Low-middle SDI** | **1764 (1432 to 2155)** | **60 (37 to 89)** | **229.06 (185.98 to 279.86)** | **10.07 (6.24 to 14.87)** | **-10.14 (-10.65 to -9.62)** | **159699 (129847 to 194523)** | **5663 (3591 to 8200)** | **20737.87 (16861.48 to 25259.97)** | **942.72 (597.73 to 1365.05)** | **-10.02 (-10.51 to -9.53)** |
| **Guatemala** | **0.540099** | **Low-middle SDI** | **5596 (5031 to 6310)** | **944 (708 to 1250)** | **364.79 (327.98 to 411.33)** | **60.51 (45.38 to 80.15)** | **-5.12 (-5.38 to -4.86)** | **501992 (451415 to 564486)** | **85297 (64294 to 112363)** | **32723.79 (29426.8 to 36797.66)** | **5470.32 (4123.32 to 7206.15)** | **-5.1 (-5.36 to -4.85)** |
| **Honduras** | **0.513586** | **Low-middle SDI** | **2149 (1752 to 2587)** | **297 (180 to 428)** | **261.17 (212.9 to 314.33)** | **27.07 (16.4 to 39.03)** | **-7.26 (-7.46 to -7.05)** | **193184 (157505 to 232220)** | **26983 (16437 to 38578)** | **23473 (19137.73 to 28216.1)** | **2463.01 (1500.4 to 3521.45)** | **-7.22 (-7.42 to -7.02)** |
| **Mexico** | **0.664969** | **NA** | **21321 (19183 to 23944)** | **753 (540 to 1023)** | **180.72 (162.6 to 202.95)** | **7.63 (5.46 to 10.36)** | **-9.57 (-9.98 to -9.16)** | **1931292 (1742291 to 2164148)** | **70966 (51571 to 95613)** | **16369.6 (14767.63 to 18343.28)** | **718.49 (522.12 to 968.03)** | **-9.46 (-9.87 to -9.05)** |
| **Nicaragua** | **0.523647** | **Low-middle SDI** | **2085 (1686 to 2693)** | **98 (59 to 144)** | **314.3 (254.06 to 405.89)** | **15.08 (9.1 to 22.12)** | **-9.77 (-10.18 to -9.36)** | **188733 (153187 to 243196)** | **9058 (5621 to 13153)** | **28444.74 (23087.37 to 36652.98)** | **1392.36 (864.11 to 2021.87)** | **-9.74 (-10.14 to -9.34)** |
| **Panama** | **0.70666** | **Middle SDI** | **164 (135 to 195)** | **58 (43 to 77)** | **57.51 (47.23 to 68.37)** | **15.62 (11.5 to 20.87)** | **-3.16 (-3.57 to -2.76)** | **15504 (12921 to 18342)** | **5699 (4306 to 7479)** | **5429.51 (4524.84 to 6423.25)** | **1535.22 (1159.79 to 2014.55)** | **-3.14 (-3.51 to -2.77)** |
| **Venezuela (Bolivarian Republic of)** | **0.5966** | **Low-middle SDI** | **3154 (2940 to 3397)** | **471 (218 to 664)** | **124.63 (116.17 to 134.22)** | **21.55 (10 to 30.38)** | **-6.1 (-7 to -5.18)** | **286276 (267309 to 307852)** | **43647 (21013 to 60597)** | **11311.72 (10562.28 to 12164.29)** | **1997.63 (961.72 to 2773.41)** | **-6.03 (-6.9 to -5.14)** |
| **Tropical Latin America** | **0.648942** | **NA** | **31553 (26931 to 36374)** | **748 (584 to 946)** | **184.75 (157.68 to 212.97)** | **4.35 (3.4 to 5.5)** | **-11.63 (-11.86 to -11.4)** | **2877930 (2457687 to 3307394)** | **76685 (61910 to 94174)** | **16850.51 (14389.96 to 19365.06)** | **445.65 (359.78 to 547.28)** | **-11.38 (-11.58 to -11.17)** |
| **Brazil** | **0.648847** | **NA** | **31031 (26383 to 35754)** | **694 (535 to 877)** | **188.5 (160.27 to 217.19)** | **4.19 (3.23 to 5.3)** | **-11.75 (-12 to -11.51)** | **2826989 (2407329 to 3254574)** | **71550 (57235 to 88225)** | **17172.64 (14623.4 to 19770.02)** | **432.13 (345.67 to 532.84)** | **-11.48 (-11.7 to -11.27)** |
| **Paraguay** | **0.650488** | **Middle SDI** | **523 (391 to 686)** | **55 (29 to 94)** | **84.72 (63.41 to 111.13)** | **8.44 (4.49 to 14.39)** | **-8.13 (-8.43 to -7.84)** | **50941 (38921 to 65339)** | **5135 (2880 to 8581)** | **8256.06 (6307.89 to 10589.59)** | **789.95 (443.03 to 1319.99)** | **-8.31 (-8.62 to -8)** |
| **North Africa and Middle East** | **0.658716** | **NA** | **90385 (67646 to 115927)** | **11473 (8080 to 17690)** | **176.43 (132.04 to 226.29)** | **18.77 (13.22 to 28.93)** | **-7.16 (-7.42 to -6.89)** | **8271492 (6236549 to 10554251)** | **1079163 (782635 to 1625428)** | **16145.92 (12173.72 to 20601.85)** | **1765.15 (1280.13 to 2658.66)** | **-7.03 (-7.29 to -6.77)** |
| **North Africa and Middle East** | **0.658716** | **NA** | **90385 (67646 to 115927)** | **11473 (8080 to 17690)** | **176.43 (132.04 to 226.29)** | **18.77 (13.22 to 28.93)** | **-7.16 (-7.42 to -6.89)** | **8271492 (6236549 to 10554251)** | **1079163 (782635 to 1625428)** | **16145.92 (12173.72 to 20601.85)** | **1765.15 (1280.13 to 2658.66)** | **-7.03 (-7.29 to -6.77)** |
| **Algeria** | **0.65972** | **Middle SDI** | **1055 (375 to 1776)** | **183 (77 to 274)** | **28.23 (10.03 to 47.53)** | **3.88 (1.64 to 5.83)** | **-5.71 (-5.86 to -5.55)** | **107468 (46833 to 172343)** | **18892 (9335 to 27296)** | **2876.56 (1253.56 to 4613.05)** | **401.55 (198.41 to 580.19)** | **-5.74 (-5.89 to -5.59)** |
| **Bahrain** | **0.752218** | **High-middle SDI** | **8 (6 to 11)** | **2 (1 to 3)** | **13.79 (10.04 to 18.5)** | **2.01 (1.27 to 2.78)** | **-5.32 (-5.67 to -4.96)** | **809 (606 to 1074)** | **208 (145 to 280)** | **1317.69 (986.39 to 1749.17)** | **222.03 (154.87 to 298.68)** | **-4.95 (-5.28 to -4.62)** |
| **Egypt** | **0.603962** | **Low-middle SDI** | **27607 (19987 to 36954)** | **2746 (1842 to 3976)** | **323.54 (234.24 to 433.08)** | **21.07 (14.13 to 30.49)** | **-8.25 (-8.58 to -7.91)** | **2507544 (1822354 to 3351168)** | **263179 (182156 to 372878)** | **29386.95 (21356.92 to 39273.73)** | **2018.65 (1397.18 to 2860.06)** | **-8.06 (-8.39 to -7.74)** |
| **Iran (Islamic Republic of)** | **0.697293** | **NA** | **3453 (2148 to 5952)** | **71 (51 to 94)** | **39.36 (24.48 to 67.85)** | **1.16 (0.83 to 1.53)** | **-9.11 (-9.7 to -8.52)** | **339721 (225450 to 566926)** | **8220 (6140 to 10464)** | **3872.73 (2570.08 to 6462.82)** | **133.55 (99.76 to 170.01)** | **-8.94 (-9.47 to -8.41)** |
| **Iraq** | **0.662777** | **Middle SDI** | **2240 (1520 to 3197)** | **490 (326 to 705)** | **71.28 (48.37 to 101.71)** | **11.4 (7.6 to 16.41)** | **-6.4 (-6.67 to -6.13)** | **211365 (147062 to 297833)** | **47055 (32257 to 66129)** | **6725.52 (4679.42 to 9476.85)** | **1095.99 (751.31 to 1540.25)** | **-6.31 (-6.58 to -6.04)** |
| **Jordan** | **0.72542** | **High-middle SDI** | **96 (65 to 137)** | **37 (25 to 54)** | **15.95 (10.81 to 22.78)** | **3.36 (2.29 to 4.89)** | **-5.23 (-5.39 to -5.08)** | **9683 (6858 to 13339)** | **3909 (2821 to 5377)** | **1613.34 (1142.67 to 2222.39)** | **356.2 (257.13 to 489.99)** | **-4.88 (-5.01 to -4.75)** |
| **Kuwait** | **0.846802** | **High SDI** | **10 (9 to 12)** | **2 (2 to 3)** | **5.13 (4.3 to 6.06)** | **0.89 (0.68 to 1.15)** | **-3.24 (-4.28 to -2.2)** | **1377 (1168 to 1629)** | **393 (303 to 513)** | **673.37 (571.21 to 796.71)** | **148.14 (114.42 to 193.59)** | **-2.93 (-3.6 to -2.25)** |
| **Lebanon** | **0.741226** | **High-middle SDI** | **102 (56 to 152)** | **26 (16 to 41)** | **26.24 (14.3 to 38.89)** | **6.49 (3.99 to 10.07)** | **-4.54 (-4.83 to -4.26)** | **9731 (5596 to 14113)** | **2697 (1821 to 4063)** | **2497.51 (1436.29 to 3622.42)** | **664.33 (448.59 to 1001.08)** | **-4.3 (-4.5 to -4.11)** |
| **Libya** | **0.735084** | **High-middle SDI** | **325 (181 to 513)** | **21 (6 to 36)** | **51.08 (28.45 to 80.51)** | **4.88 (1.33 to 8.55)** | **-6.84 (-7.72 to -5.95)** | **30560 (17712 to 47576)** | **2031 (687 to 3446)** | **4798.15 (2780.87 to 7469.65)** | **480.02 (162.47 to 814.4)** | **-6.67 (-7.42 to -5.91)** |
| **Morocco** | **0.56168** | **Low-middle SDI** | **11012 (8401 to 14144)** | **633 (384 to 998)** | **309.29 (235.95 to 397.26)** | **19.47 (11.81 to 30.71)** | **-8.54 (-8.83 to -8.26)** | **1002590 (771512 to 1282824)** | **58774 (36088 to 91763)** | **28159.59 (21669.34 to 36030.51)** | **1807.98 (1110.13 to 2822.8)** | **-8.5 (-8.79 to -8.21)** |
| **Palestine** | **0.629202** | **Middle SDI** | **151 (97 to 236)** | **16 (10 to 24)** | **38.64 (24.91 to 60.48)** | **2.57 (1.67 to 3.94)** | **-7.9 (-8.28 to -7.51)** | **13958 (9163 to 21529)** | **1583 (1071 to 2340)** | **3579.06 (2349.4 to 5520.44)** | **258.39 (174.71 to 381.93)** | **-7.45 (-7.84 to -7.06)** |
| **Oman** | **0.773801** | **High-middle SDI** | **76 (41 to 134)** | **17 (11 to 25)** | **23.24 (12.37 to 40.89)** | **3.99 (2.59 to 5.88)** | **-4.47 (-5.44 to -3.5)** | **7359 (4102 to 12486)** | **1680 (1142 to 2368)** | **2245.74 (1251.9 to 3810.55)** | **396.18 (269.29 to 558.45)** | **-4.42 (-5.35 to -3.48)** |
| **Qatar** | **0.846704** | **High SDI** | **3 (2 to 4)** | **2 (1 to 3)** | **5.88 (3.75 to 8.76)** | **1.27 (0.81 to 1.84)** | **-4.44 (-4.64 to -4.24)** | **321 (222 to 448)** | **316 (226 to 426)** | **631.06 (437.59 to 881.19)** | **171.59 (122.8 to 231)** | **-3.82 (-3.99 to -3.65)** |
| **Saudi Arabia** | **0.814516** | **High SDI** | **1205 (753 to 1917)** | **68 (41 to 102)** | **49.85 (31.15 to 79.29)** | **2.79 (1.68 to 4.19)** | **-9.08 (-9.25 to -8.9)** | **113494 (73680 to 176371)** | **7316 (4728 to 10447)** | **4694.3 (3047.53 to 7294.99)** | **300.67 (194.34 to 429.37)** | **-8.65 (-8.84 to -8.45)** |
| **Syrian Arab Republic** | **0.622856** | **Middle SDI** | **731 (476 to 1093)** | **24 (12 to 38)** | **33.89 (22.05 to 50.68)** | **2.41 (1.18 to 3.76)** | **-7.57 (-8.38 to -6.75)** | **71974 (49717 to 104653)** | **2740 (1612 to 3882)** | **3336.84 (2304.98 to 4851.93)** | **272.65 (160.39 to 386.2)** | **-7.23 (-7.88 to -6.57)** |
| **Tunisia** | **0.681701** | **Middle SDI** | **297 (126 to 474)** | **25 (9 to 40)** | **27.82 (11.76 to 44.4)** | **2.78 (1.03 to 4.52)** | **-6.56 (-6.91 to -6.21)** | **29326 (14012 to 44661)** | **2911 (1449 to 4347)** | **2748.06 (1313.04 to 4185.08)** | **326.3 (162.44 to 487.26)** | **-6.04 (-6.36 to -5.72)** |
| **Turkey** | **0.713246** | **High-middle SDI** | **5220 (3568 to 8234)** | **239 (170 to 348)** | **75.05 (51.3 to 118.39)** | **4.3 (3.06 to 6.27)** | **-9.31 (-9.52 to -9.1)** | **488868 (339294 to 756768)** | **24240 (17893 to 33769)** | **7028.96 (4878.38 to 10880.84)** | **436.6 (322.29 to 608.24)** | **-9 (-9.15 to -8.84)** |
| **United Arab Emirates** | **0.84974** | **High SDI** | **23 (14 to 34)** | **11 (8 to 16)** | **10.1 (5.99 to 14.74)** | **2.53 (1.8 to 3.6)** | **-2.26 (-3 to -1.52)** | **2373 (1527 to 3348)** | **1222 (916 to 1653)** | **1040.96 (669.81 to 1468.91)** | **282.35 (211.6 to 382.12)** | **-2.39 (-3.03 to -1.75)** |
| **Yemen** | **0.45354** | **Low SDI** | **14165 (9317 to 20497)** | **1300 (453 to 2668)** | **510.14 (335.55 to 738.21)** | **27.69 (9.64 to 56.79)** | **-9.18 (-9.67 to -8.7)** | **1281938 (848926 to 1847065)** | **121757 (45578 to 243177)** | **46169.64 (30574.47 to 66522.94)** | **2592.05 (970.31 to 5176.95)** | **-8.86 (-9.29 to -8.44)** |
| **South Asia** | **0.559643** | **NA** | **613477 (489340 to 736962)** | **56161 (34503 to 81505)** | **390.69 (311.63 to 469.33)** | **35.41 (21.76 to 51.39)** | **-7.21 (-7.56 to -6.87)** | **55270939 (44154166 to 66228228)** | **5181856 (3276948 to 7440275)** | **35198.65 (28119.06 to 42176.67)** | **3267.38 (2066.25 to 4691.4)** | **-7.14 (-7.48 to -6.8)** |
| **South Asia** | **0.559643** | **NA** | **613477 (489340 to 736962)** | **56161 (34503 to 81505)** | **390.69 (311.63 to 469.33)** | **35.41 (21.76 to 51.39)** | **-7.21 (-7.56 to -6.87)** | **55270939 (44154166 to 66228228)** | **5181856 (3276948 to 7440275)** | **35198.65 (28119.06 to 42176.67)** | **3267.38 (2066.25 to 4691.4)** | **-7.14 (-7.48 to -6.8)** |
| **Afghanistan** | **0.335068** | **Low SDI** | **7324 (4692 to 10605)** | **3956 (2551 to 5981)** | **427.27 (273.74 to 618.69)** | **72.17 (46.53 to 109.11)** | **-6.03 (-6.66 to -5.4)** | **658539 (425214 to 950116)** | **357718 (232504 to 538230)** | **38417.34 (24805.76 to 55427.09)** | **6525.98 (4241.65 to 9819.13)** | **-6.01 (-6.64 to -5.38)** |
| **Bangladesh** | **0.493106** | **Low-middle SDI** | **48399 (35899 to 71847)** | **2281 (1421 to 3744)** | **255.66 (189.63 to 379.52)** | **15.88 (9.9 to 26.06)** | **-8.71 (-9.05 to -8.36)** | **4383982 (3267017 to 6467885)** | **206678 (129417 to 336806)** | **23157.78 (17257.57 to 34165.71)** | **1438.86 (900.97 to 2344.79)** | **-8.68 (-9.01 to -8.36)** |
| **Bhutan** | **0.476725** | **Low-middle SDI** | **581 (257 to 972)** | **24 (11 to 42)** | **608.84 (269.39 to 1018.34)** | **39.06 (18.82 to 69.01)** | **-9.16 (-9.4 to -8.92)** | **51897 (23124 to 86546)** | **2146 (1045 to 3777)** | **54361.06 (24222.1 to 90655.7)** | **3515.63 (1711.58 to 6188.75)** | **-9.12 (-9.35 to -8.88)** |
| **India** | **0.577738** | **NA** | **472092 (360096 to 585588)** | **37874 (21994 to 56748)** | **406.12 (309.77 to 503.76)** | **34.02 (19.75 to 50.97)** | **-7.59 (-7.99 to -7.19)** | **42484114 (32597282 to 52593044)** | **3507755 (2132904 to 5176638)** | **36547.21 (28042.01 to 45243.48)** | **3150.59 (1915.73 to 4649.55)** | **-7.51 (-7.9 to -7.11)** |
| **Nepal** | **0.433953** | **Low SDI** | **14010 (10400 to 18072)** | **623 (342 to 939)** | **425.41 (315.79 to 548.73)** | **20.04 (11 to 30.24)** | **-9.45 (-9.76 to -9.15)** | **1262955 (937634 to 1624690)** | **56928 (32234 to 85369)** | **38348.31 (28470.29 to 49332.03)** | **1832.61 (1037.68 to 2748.17)** | **-9.38 (-9.69 to -9.07)** |
| **Pakistan** | **0.504276** | **NA** | **78395 (62125 to 98593)** | **15359 (9112 to 25190)** | **424.64 (336.51 to 534.05)** | **51.67 (30.65 to 84.74)** | **-5.71 (-6.14 to -5.29)** | **7087992 (5620010 to 8887104)** | **1408349 (854516 to 2281534)** | **38393.4 (30441.81 to 48138.62)** | **4737.77 (2874.64 to 7675.21)** | **-5.67 (-6.1 to -5.24)** |
| **Central Sub-Saharan Africa** | **0.484518** | **NA** | **60617 (42180 to 76496)** | **13404 (8218 to 20759)** | **583.73 (406.19 to 736.64)** | **63.63 (39.01 to 98.54)** | **-6.73 (-7.69 to -5.77)** | **5452145 (3802717 to 6858692)** | **1220197 (757095 to 1880530)** | **52503.2 (36619.49 to 66048)** | **5792.12 (3593.83 to 8926.64)** | **-6.69 (-7.65 to -5.73)** |
| **Angola** | **0.482946** | **Low-middle SDI** | **21105 (14513 to 28671)** | **3387 (2126 to 4852)** | **1081.09 (743.45 to 1468.68)** | **60.13 (37.75 to 86.14)** | **-8.99 (-9.73 to -8.24)** | **1887317 (1301411 to 2557388)** | **309002 (196664 to 438964)** | **96678.18 (66665.02 to 131002.67)** | **5485.38 (3491.16 to 7792.45)** | **-8.93 (-9.67 to -8.18)** |
| **Central African Republic** | **0.311027** | **Low SDI** | **3878 (2323 to 5318)** | **2638 (1527 to 4284)** | **769.95 (461.27 to 1055.7)** | **314.52 (182 to 510.73)** | **-2.46 (-2.68 to -2.25)** | **348399 (210175 to 476607)** | **236047 (137194 to 382514)** | **69168.56 (41726.67 to 94622.09)** | **28139.58 (16355.14 to 45600.22)** | **-2.48 (-2.69 to -2.27)** |
| **Congo** | **0.586909** | **Low-middle SDI** | **1608 (1023 to 2447)** | **344 (153 to 723)** | **407.57 (259.27 to 620.28)** | **54.43 (24.23 to 114.23)** | **-6.21 (-6.83 to -5.59)** | **145590 (93547 to 220456)** | **31468 (14418 to 65335)** | **36905.68 (23713.19 to 55883.51)** | **4972.54 (2278.35 to 10323.98)** | **-6.17 (-6.79 to -5.55)** |
| **Democratic Republic of the Congo** | **0.390178** | **Low SDI** | **32929 (22345 to 44036)** | **6942 (3333 to 13141)** | **451.35 (306.28 to 603.6)** | **51.19 (24.58 to 96.91)** | **-6.43 (-7.66 to -5.19)** | **2971986 (2024714 to 3957936)** | **635209 (310423 to 1187962)** | **40736.68 (27752.52 to 54250.98)** | **4684.18 (2289.13 to 8760.31)** | **-6.39 (-7.61 to -5.15)** |
| **Equatorial Guinea** | **0.663978** | **Middle SDI** | **714 (402 to 1026)** | **39 (20 to 70)** | **867.53 (488.95 to 1247.25)** | **20.85 (10.51 to 37.21)** | **-13.31 (-13.93 to -12.69)** | **64010 (36274 to 91647)** | **3613 (1897 to 6344)** | **77813.79 (44097.06 to 111411.33)** | **1928.61 (1012.47 to 3386.12)** | **-13.16 (-13.75 to -12.57)** |
| **Gabon** | **0.639081** | **Middle SDI** | **384 (194 to 711)** | **53 (17 to 122)** | **245.55 (123.89 to 455.1)** | **24.62 (7.88 to 56.95)** | **-6.87 (-7.21 to -6.53)** | **34844 (17910 to 64143)** | **4858 (1709 to 11017)** | **22308.54 (11466.51 to 41067.2)** | **2274.79 (800.29 to 5159.14)** | **-6.81 (-7.16 to -6.46)** |
| **Eastern Sub-Saharan Africa** | **0.412188** | **NA** | **204487 (128877 to 280209)** | **66667 (46246 to 96386)** | **566.66 (357.13 to 776.49)** | **104.5 (72.49 to 151.08)** | **-5.33 (-5.5 to -5.16)** | **18376539 (11668580 to 25086522)** | **5994967 (4175442 to 8646755)** | **50923.68 (32335.09 to 69517.88)** | **9397.03 (6544.95 to 13553.68)** | **-5.32 (-5.5 to -5.15)** |
| **Burundi** | **0.291289** | **Low SDI** | **4323 (2684 to 6367)** | **1925 (793 to 4103)** | **402.06 (249.66 to 592.19)** | **89.11 (36.71 to 189.94)** | **-4.17 (-4.69 to -3.64)** | **388787 (243580 to 569589)** | **173964 (73331 to 366966)** | **36162.89 (22656.49 to 52980.15)** | **8052.44 (3394.34 to 16986.14)** | **-4.15 (-4.68 to -3.62)** |
| **Comoros** | **0.476956** | **Low-middle SDI** | **228 (134 to 364)** | **41 (20 to 70)** | **276.52 (162.84 to 441.66)** | **50.6 (24.61 to 86.21)** | **-5.32 (-5.76 to -4.89)** | **20752 (12465 to 32721)** | **3752 (1872 to 6343)** | **25179.62 (15123.7 to 39701.58)** | **4614.4 (2302.59 to 7801.26)** | **-5.31 (-5.75 to -4.87)** |
| **Djibouti** | **0.4892** | **Low-middle SDI** | **292 (193 to 422)** | **66 (31 to 123)** | **452.24 (298.51 to 653.02)** | **45.23 (21.52 to 84.53)** | **-7.09 (-7.52 to -6.66)** | **26354 (17476 to 37907)** | **5961 (2895 to 11004)** | **40776.88 (27039.88 to 58653.67)** | **4091.23 (1987.14 to 7552.73)** | **-7.08 (-7.51 to -6.65)** |
| **Eritrea** | **0.404572** | **Low SDI** | **4322 (2992 to 5696)** | **852 (496 to 1374)** | **693.19 (479.94 to 913.55)** | **92.86 (54.05 to 149.7)** | **-6.38 (-6.58 to -6.18)** | **387154 (269447 to 508235)** | **76643 (44945 to 122428)** | **62094.27 (43215.64 to 81513.97)** | **8348.63 (4895.8 to 13335.85)** | **-6.36 (-6.56 to -6.16)** |
| **Ethiopia** | **0.360728** | **NA** | **55000 (27558 to 85677)** | **13615 (9241 to 20606)** | **570.57 (285.89 to 888.83)** | **85.28 (57.89 to 129.07)** | **-6.3 (-6.46 to -6.14)** | **4936889 (2505279 to 7657481)** | **1230931 (841708 to 1851590)** | **51216.18 (25990.22 to 79440.1)** | **7710.13 (5272.17 to 11597.72)** | **-6.26 (-6.42 to -6.1)** |
| **Kenya** | **0.524783** | **NA** | **16979 (10277 to 22158)** | **4823 (3568 to 6308)** | **395.51 (239.4 to 516.14)** | **81.05 (59.97 to 106.02)** | **-4.61 (-5.05 to -4.16)** | **1534609 (935680 to 1993147)** | **439705 (327818 to 573578)** | **35746.84 (21795.52 to 46427.92)** | **7389.77 (5509.37 to 9639.67)** | **-4.59 (-5.04 to -4.14)** |
| **Madagascar** | **0.401385** | **Low SDI** | **15409 (12190 to 18829)** | **8676 (4902 to 14210)** | **714.85 (565.53 to 873.51)** | **212.24 (119.91 to 347.59)** | **-3.31 (-3.63 to -2.99)** | **1382686 (1098801 to 1685320)** | **776397 (441619 to 1268605)** | **64143.93 (50974.27 to 78183.4)** | **18991.82 (10802.66 to 31031.98)** | **-3.32 (-3.64 to -3)** |
| **Malawi** | **0.381986** | **Low SDI** | **16749 (10513 to 22074)** | **2346 (1372 to 3727)** | **882.1 (553.7 to 1162.55)** | **86.14 (50.36 to 136.81)** | **-7.44 (-7.61 to -7.27)** | **1500957 (948768 to 1975812)** | **211195 (124662 to 334313)** | **79050.66 (49968.61 to 104059.76)** | **7752.97 (4576.32 to 12272.62)** | **-7.42 (-7.6 to -7.24)** |
| **Mauritius** | **0.717977** | **High-middle SDI** | **28 (25 to 31)** | **4 (3 to 5)** | **26.08 (23.29 to 29.6)** | **6.6 (5.2 to 7.88)** | **-2.5 (-3.02 to -1.98)** | **2920 (2612 to 3283)** | **480 (394 to 562)** | **2768.82 (2476.74 to 3112.69)** | **747.09 (612.36 to 873.5)** | **-2.47 (-2.93 to -2)** |
| **Mozambique** | **0.327475** | **Low SDI** | **14976 (8895 to 23082)** | **3902 (2238 to 6766)** | **621.67 (369.22 to 958.18)** | **75.34 (43.21 to 130.64)** | **-6.63 (-6.96 to -6.31)** | **1344330 (805540 to 2062542)** | **349619 (201313 to 603122)** | **55804.41 (33438.74 to 85618.06)** | **6750.43 (3886.93 to 11645.04)** | **-6.64 (-6.97 to -6.31)** |
| **Rwanda** | **0.43614** | **Low SDI** | **6235 (3723 to 8889)** | **1191 (743 to 1803)** | **462.58 (276.2 to 659.47)** | **68.13 (42.49 to 103.11)** | **-7 (-7.78 to -6.21)** | **561484 (338705 to 798958)** | **107031 (67225 to 161346)** | **41657.32 (25129.04 to 59275.83)** | **6121.43 (3844.8 to 9227.91)** | **-6.99 (-7.77 to -6.2)** |
| **Seychelles** | **0.727579** | **High-middle SDI** | **1 (1 to 2)** | **0 (0 to 1)** | **15.5 (11.17 to 21.21)** | **6.19 (3.88 to 9.1)** | **-2.34 (-2.88 to -1.79)** | **123 (93 to 164)** | **49 (32 to 71)** | **1522.62 (1153.33 to 2031.99)** | **626.23 (411.95 to 900.43)** | **-2.23 (-2.74 to -1.71)** |
| **Somalia** | **0.077434** | **Low SDI** | **12425 (7023 to 17847)** | **9794 (5600 to 14582)** | **804.18 (454.53 to 1155.13)** | **237.2 (135.62 to 353.16)** | **-4.04 (-4.22 to -3.86)** | **1111297 (632027 to 1593625)** | **875901 (502877 to 1298923)** | **71926.8 (40906.89 to 103144.69)** | **21213.04 (12178.94 to 31457.99)** | **-4.04 (-4.22 to -3.86)** |
| **United Republic of Tanzania** | **0.448566** | **Low SDI** | **21715 (15269 to 29883)** | **6200 (3503 to 10073)** | **451.64 (317.57 to 621.52)** | **70.08 (39.6 to 113.87)** | **-5.8 (-6.09 to -5.51)** | **1958893 (1383154 to 2685988)** | **555853 (315692 to 898918)** | **40742.16 (28767.61 to 55864.69)** | **6283.55 (3568.69 to 10161.67)** | **-5.82 (-6.11 to -5.52)** |
| **Uganda** | **0.426554** | **Low SDI** | **16284 (7073 to 25887)** | **3899 (1879 to 7312)** | **453.4 (196.95 to 720.8)** | **53.28 (25.68 to 99.92)** | **-6.69 (-6.93 to -6.44)** | **1469178 (651064 to 2325638)** | **352709 (174910 to 656140)** | **40907.48 (18128.08 to 64754.57)** | **4819.93 (2390.22 to 8966.46)** | **-6.67 (-6.91 to -6.42)** |
| **Zambia** | **0.51023** | **Low-middle SDI** | **9850 (6229 to 13736)** | **2258 (1404 to 3413)** | **652.71 (412.73 to 910.15)** | **77.2 (47.99 to 116.69)** | **-6.72 (-7.35 to -6.09)** | **884625 (565389 to 1228286)** | **203406 (127717 to 306298)** | **58616.76 (37463.63 to 81388.33)** | **6953.97 (4366.33 to 10471.58)** | **-6.71 (-7.34 to -6.07)** |
| **Southern Sub-Saharan Africa** | **0.643348** | **NA** | **23642 (20416 to 27215)** | **8101 (5953 to 10797)** | **316.37 (273.2 to 364.18)** | **100.89 (74.14 to 134.47)** | **-3.31 (-3.82 to -2.8)** | **2148040 (1855248 to 2468975)** | **730458 (539617 to 969537)** | **28744.38 (24826.34 to 33039.04)** | **9097.62 (6720.75 to 12075.27)** | **-3.34 (-3.85 to -2.83)** |
| **Botswana** | **0.643078** | **Middle SDI** | **585 (441 to 760)** | **296 (181 to 442)** | **274.8 (206.97 to 356.67)** | **125.55 (76.98 to 187.76)** | **-2.45 (-2.93 to -1.96)** | **53028 (40090 to 68637)** | **26610 (16486 to 39544)** | **24893.4 (18819.52 to 32220.51)** | **11299.24 (7000.64 to 16791.7)** | **-2.47 (-2.96 to -1.98)** |
| **Lesotho** | **0.511571** | **Low-middle SDI** | **1345 (1095 to 1663)** | **609 (392 to 859)** | **546.27 (444.46 to 675.36)** | **299.09 (192.27 to 421.65)** | **-1.86 (-2.16 to -1.57)** | **121235 (98606 to 149761)** | **54623 (35234 to 76926)** | **49221.7 (40034.51 to 60803.6)** | **26813.51 (17295.81 to 37761.47)** | **-1.89 (-2.18 to -1.59)** |
| **Namibia** | **0.618074** | **Low-middle SDI** | **796 (586 to 1037)** | **336 (195 to 496)** | **351.76 (258.74 to 458.14)** | **120.69 (70 to 178.01)** | **-3.02 (-3.39 to -2.65)** | **71875 (53289 to 93262)** | **30214 (17612 to 44376)** | **31752.62 (23541.61 to 41200.9)** | **10850.61 (6324.92 to 15936.52)** | **-3.03 (-3.4 to -2.66)** |
| **South Africa** | **0.681292** | **NA** | **17926 (15177 to 20832)** | **4690 (3572 to 6073)** | **367.47 (311.11 to 427.04)** | **94.53 (71.99 to 122.41)** | **-3.97 (-4.46 to -3.47)** | **1628376 (1380569 to 1885976)** | **424284 (325440 to 546936)** | **33380.19 (28300.38 to 38660.75)** | **8551.85 (6559.56 to 11024.02)** | **-3.99 (-4.48 to -3.5)** |
| **Eswatini** | **0.586217** | **Low-middle SDI** | **714 (543 to 895)** | **198 (122 to 306)** | **490.84 (373.16 to 615.15)** | **140.68 (86.96 to 218.2)** | **-3.94 (-4.57 to -3.3)** | **64365 (49136 to 80399)** | **17791 (11025 to 27469)** | **44263.3 (33790.33 to 55289.47)** | **12670.39 (7851.63 to 19562.54)** | **-3.94 (-4.58 to -3.3)** |
| **Zimbabwe** | **0.475577** | **Low-middle SDI** | **2275 (1596 to 2877)** | **1972 (986 to 2888)** | **129 (90.52 to 163.12)** | **89.25 (44.61 to 130.7)** | **-0.55 (-1.53 to 0.43)** | **209160 (149695 to 262819)** | **176937 (88833 to 258658)** | **11860.29 (8488.34 to 14902.97)** | **8007.2 (4020.1 to 11705.46)** | **-0.63 (-1.6 to 0.35)** |
| **Western Sub-Saharan Africa** | **0.446421** | **NA** | **289819 (202388 to 360182)** | **157796 (106245 to 230123)** | **810.81 (566.21 to 1007.66)** | **197.35 (132.88 to 287.8)** | **-4.36 (-4.75 to -3.96)** | **25924733 (18175757 to 32135520)** | **14121036 (9586422 to 20521000)** | **72528.16 (50849.28 to 89903.72)** | **17660.54 (11989.31 to 25664.69)** | **-4.35 (-4.74 to -3.96)** |
| **Benin** | **0.374522** | **Low SDI** | **4847 (3146 to 6837)** | **1911 (874 to 3926)** | **491.23 (318.81 to 692.9)** | **81.59 (37.31 to 167.63)** | **-5.37 (-5.66 to -5.09)** | **434859 (284924 to 611842)** | **171504 (79295 to 350011)** | **44071.47 (28876.07 to 62008.06)** | **7321.82 (3385.27 to 14942.64)** | **-5.37 (-5.66 to -5.09)** |
| **Burkina Faso** | **0.284471** | **Low SDI** | **14586 (9700 to 19819)** | **7405 (4897 to 10958)** | **777.28 (516.91 to 1056.14)** | **180.34 (119.25 to 266.86)** | **-4.63 (-4.9 to -4.35)** | **1306480 (872570 to 1770582)** | **662300 (438997 to 975850)** | **69622.31 (46499.26 to 94354.32)** | **16128.23 (10690.38 to 23763.74)** | **-4.63 (-4.91 to -4.35)** |
| **Cameroon** | **0.480365** | **Low-middle SDI** | **7379 (4671 to 11199)** | **4273 (2040 to 8341)** | **367.76 (232.8 to 558.15)** | **87.85 (41.94 to 171.49)** | **-4.1 (-4.79 to -3.4)** | **666430 (426311 to 1005441)** | **385303 (187266 to 747383)** | **33215.17 (21247.51 to 50111.63)** | **7921.99 (3850.26 to 15366.51)** | **-4.1 (-4.8 to -3.4)** |
| **Cabo Verde** | **0.533601** | **Low-middle SDI** | **160 (112 to 218)** | **9 (6 to 13)** | **269.01 (187.62 to 365.59)** | **20.48 (12.94 to 30.2)** | **-8.54 (-9.21 to -7.85)** | **14576 (10262 to 19678)** | **819 (520 to 1204)** | **24495.02 (17244.88 to 33069.41)** | **1858.92 (1179.39 to 2732.3)** | **-8.57 (-9.23 to -7.9)** |
| **Chad** | **0.243517** | **Low SDI** | **15060 (9719 to 20634)** | **20375 (13589 to 34632)** | **1211.62 (781.92 to 1660.14)** | **560.45 (373.77 to 952.59)** | **-2.37 (-2.56 to -2.18)** | **1343207 (870890 to 1839287)** | **1814904 (1214255 to 3079256)** | **108068.79 (70068.17 to 147981.31)** | **49921.52 (33399.8 to 84699.3)** | **-2.37 (-2.56 to -2.18)** |
| **C么te d'Ivoire** | **0.424541** | **Low SDI** | **8435 (5916 to 11621)** | **3369 (1880 to 5730)** | **365.09 (256.04 to 502.96)** | **77.36 (43.18 to 131.59)** | **-4.32 (-4.78 to -3.87)** | **761258 (537743 to 1044454)** | **303531 (171640 to 513452)** | **32948.43 (23274.38 to 45205.6)** | **6970.68 (3941.77 to 11791.58)** | **-4.33 (-4.79 to -3.87)** |
| **Gambia** | **0.410077** | **Low SDI** | **767 (504 to 1045)** | **223 (148 to 314)** | **413.01 (271.53 to 562.9)** | **62.31 (41.49 to 87.81)** | **-6.16 (-6.48 to -5.84)** | **68627 (45572 to 93417)** | **19892 (13310 to 27954)** | **36957.69 (24541.59 to 50307.67)** | **5562.85 (3722.05 to 7817.32)** | **-6.16 (-6.48 to -5.83)** |
| **Ghana** | **0.563348** | **Low-middle SDI** | **7724 (4845 to 10936)** | **1494 (886 to 2258)** | **293.24 (183.93 to 415.19)** | **32.2 (19.09 to 48.67)** | **-6.6 (-6.96 to -6.25)** | **702064 (447877 to 986510)** | **135559 (81309 to 203516)** | **26653.78 (17003.61 to 37452.74)** | **2921.44 (1752.3 to 4386)** | **-6.6 (-6.97 to -6.24)** |
| **Guinea** | **0.336555** | **Low SDI** | **8035 (5262 to 11152)** | **1393 (623 to 2772)** | **692.36 (453.38 to 960.97)** | **61.88 (27.67 to 123.16)** | **-7.11 (-7.45 to -6.78)** | **719414 (473171 to 994598)** | **125455 (56823 to 248201)** | **61989.53 (40771.57 to 85701.25)** | **5574.61 (2524.95 to 11028.84)** | **-7.1 (-7.44 to -6.76)** |
| **Guinea-Bissau** | **0.353448** | **Low SDI** | **1172 (819 to 1617)** | **215 (120 to 349)** | **622.9 (435.33 to 859.88)** | **64.66 (36.16 to 104.79)** | **-7.25 (-8.14 to -6.36)** | **104738 (73542 to 144117)** | **19319 (10913 to 31136)** | **55689.11 (39102.18 to 76626.78)** | **5807.61 (3280.6 to 9359.81)** | **-7.24 (-8.12 to -6.34)** |
| **Liberia** | **0.353229** | **Low SDI** | **4520 (3216 to 5823)** | **952 (492 to 1825)** | **973.26 (692.5 to 1253.91)** | **124.18 (64.16 to 238.17)** | **-6.89 (-7.27 to -6.51)** | **404880 (289088 to 521937)** | **85587 (44736 to 162949)** | **87178.84 (62246.46 to 112383.66)** | **11167.59 (5837.23 to 21261.97)** | **-6.86 (-7.25 to -6.48)** |
| **Mali** | **0.271176** | **Low SDI** | **11182 (6857 to 15980)** | **4081 (2610 to 6331)** | **646.67 (396.52 to 924.17)** | **89.1 (56.99 to 138.22)** | **-6.02 (-6.39 to -5.65)** | **1002282 (620562 to 1425207)** | **366089 (235866 to 567430)** | **57963.07 (35887.77 to 82421.32)** | **7992.54 (5149.49 to 12388.28)** | **-6.02 (-6.39 to -5.64)** |
| **Mauritania** | **0.495267** | **Low-middle SDI** | **1309 (794 to 1998)** | **318 (155 to 624)** | **352.91 (214.13 to 538.73)** | **48.38 (23.53 to 95.04)** | **-6.3 (-6.92 to -5.69)** | **117846 (72130 to 179585)** | **28993 (14462 to 56195)** | **31769 (19444.97 to 48412.72)** | **4412.9 (2201.17 to 8553.22)** | **-6.24 (-6.86 to -5.62)** |
| **Niger** | **0.17031** | **Low SDI** | **23016 (16896 to 29458)** | **10238 (6320 to 16433)** | **1364.31 (1001.57 to 1746.19)** | **200.89 (124.01 to 322.46)** | **-6.5 (-6.91 to -6.09)** | **2049782 (1511076 to 2621078)** | **915670 (569110 to 1463894)** | **121505.37 (89572.37 to 155370.18)** | **17967.2 (11167.03 to 28724.41)** | **-6.48 (-6.89 to -6.07)** |
| **Nigeria** | **0.503699** | **NA** | **162714 (110934 to 202326)** | **97680 (63507 to 141995)** | **1023.67 (697.91 to 1272.88)** | **263.16 (171.1 to 382.55)** | **-4.12 (-4.55 to -3.69)** | **14533038 (9930542 to 18028604)** | **8738219 (5711410 to 12653316)** | **91430.42 (62475.14 to 113421.76)** | **23541.83 (15387.24 to 34089.58)** | **-4.11 (-4.55 to -3.68)** |
| **Sao Tome and Principe** | **0.503306** | **Low-middle SDI** | **88 (67 to 111)** | **4 (3 to 6)** | **429.52 (325.92 to 540.91)** | **16.65 (10.44 to 25.34)** | **-10.26 (-10.88 to -9.64)** | **7919 (6048 to 9948)** | **375 (236 to 567)** | **38664.44 (29528.05 to 48574.9)** | **1503.67 (946.91 to 2275.06)** | **-10.23 (-10.85 to -9.61)** |
| **Senegal** | **0.409005** | **Low SDI** | **9300 (6940 to 11819)** | **999 (599 to 1509)** | **634.77 (473.72 to 806.76)** | **43.97 (26.35 to 66.42)** | **-8.34 (-8.95 to -7.73)** | **832126 (626110 to 1055206)** | **91018 (55789 to 136022)** | **56798.29 (42736.3 to 72025.08)** | **4007.05 (2456.1 to 5988.35)** | **-8.27 (-8.88 to -7.65)** |
| **Sierra Leone** | **0.359009** | **Low SDI** | **5453 (3564 to 7980)** | **1429 (880 to 2360)** | **704.73 (460.62 to 1031.26)** | **106.43 (65.52 to 175.76)** | **-6.32 (-6.95 to -5.68)** | **489534 (321833 to 714374)** | **128202 (79321 to 210356)** | **63263.91 (41591.47 to 92320.73)** | **9546.31 (5906.48 to 15663.69)** | **-6.31 (-6.94 to -5.68)** |
| **Togo** | **0.410016** | **Low SDI** | **4064 (2629 to 6063)** | **1426 (726 to 2599)** | **592.18 (383.02 to 883.45)** | **121.63 (61.95 to 221.67)** | **-4.64 (-5.11 to -4.16)** | **364818 (237584 to 541238)** | **128133 (66016 to 232189)** | **53157.45 (34618.13 to 78863.45)** | **10928.08 (5630.31 to 19802.73)** | **-4.63 (-5.11 to -4.16)** |
| **American Samoa** | **0.726268** | **High-middle SDI** | **1 (1 to 1)** | **0 (0 to 0)** | **11.72 (7.7 to 17.28)** | **7.98 (4.98 to 12.26)** | **-1.13 (-1.42 to -0.83)** | **88 (60 to 126)** | **30 (20 to 44)** | **1153.74 (791.41 to 1651.31)** | **803.14 (533.41 to 1181.73)** | **-1.05 (-1.29 to -0.81)** |
| **Bermuda** | **0.82132** | **High SDI** | **0 (0 to 0)** | **0 (0 to 0)** | **4.57 (3.58 to 5.65)** | **0.6 (0.4 to 0.79)** | **-6.4 (-7.25 to -5.55)** | **21 (17 to 26)** | **3 (2 to 5)** | **483.5 (389.12 to 593.62)** | **132.55 (96.74 to 181.54)** | **-3.92 (-4.46 to -3.38)** |
| **Cook Islands** | **0.778252** | **High-middle SDI** | **0 (0 to 0)** | **0 (0 to 0)** | **2.18 (1.4 to 3.33)** | **1.5 (0.91 to 2.35)** | **-3.8 (-4.7 to -2.89)** | **7 (5 to 9)** | **3 (2 to 4)** | **307.84 (222.58 to 415.94)** | **261.26 (182.55 to 351.37)** | **-1.49 (-1.85 to -1.12)** |
| **Greenland** | **0.83564** | **High SDI** | **0 (0 to 0)** | **0 (0 to 0)** | **3.48 (1.55 to 5.32)** | **1.32 (0.87 to 1.98)** | **-2.23 (-2.51 to -1.95)** | **19 (10 to 28)** | **5 (4 to 8)** | **339.41 (172.71 to 501.38)** | **127.06 (87.1 to 185.75)** | **-2.36 (-2.6 to -2.12)** |
| **Guam** | **0.802168** | **High-middle SDI** | **1 (0 to 1)** | **1 (0 to 1)** | **4.37 (3.09 to 6.23)** | **4.51 (2.94 to 6.72)** | **0.91 (0.44 to 1.39)** | **81 (61 to 109)** | **68 (49 to 94)** | **503.56 (378.23 to 671.52)** | **531.59 (384.48 to 737.82)** | **0.87 (0.56 to 1.18)** |
| **Monaco** | **0.909519** | **High SDI** | **0 (0 to 0)** | **0 (0 to 0)** | **0.25 (0.12 to 0.4)** | **0.25 (0.16 to 0.39)** | **-2.16 (-3.04 to -1.27)** | **2 (1 to 3)** | **3 (2 to 5)** | **187.98 (123.51 to 282.96)** | **185.78 (118.47 to 285.26)** | **0.18 (-0.26 to 0.62)** |
| **Nauru** | **0.62755** | **Middle SDI** | **0 (0 to 1)** | **0 (0 to 0)** | **30.58 (19.56 to 45.04)** | **13.87 (8.45 to 21.51)** | **-2.56 (-3.18 to -1.94)** | **47 (31 to 68)** | **19 (12 to 28)** | **2856.42 (1877.71 to 4161.79)** | **1330.52 (860.19 to 1986.95)** | **-2.49 (-3.09 to -1.89)** |
| **Niue** | **0.726219** | **High-middle SDI** | **0 (0 to 0)** | **0 (0 to 0)** | **17.8 (12.16 to 26.42)** | **26.62 (14.16 to 37.79)** | **-1.4 (-2.32 to -0.46)** | **4 (3 to 6)** | **3 (2 to 4)** | **1722.49 (1218.81 to 2470.52)** | **2468.79 (1368.16 to 3451.09)** | **-1.26 (-2.09 to -0.43)** |
| **Northern Mariana Islands** | **0.777505** | **High-middle SDI** | **0 (0 to 0)** | **0 (0 to 0)** | **3.92 (2.6 to 5.72)** | **2.52 (1.67 to 3.61)** | **-0.76 (-1.12 to -0.41)** | **22 (16 to 30)** | **11 (8 to 15)** | **473.1 (342.93 to 633.71)** | **348.34 (259.47 to 464.49)** | **-0.54 (-0.76 to -0.33)** |
| **Palau** | **0.75459** | **High-middle SDI** | **1 (0 to 1)** | **0 (0 to 0)** | **37.05 (21.63 to 62.1)** | **12.88 (8.61 to 18.98)** | **-3.24 (-3.4 to -3.09)** | **52 (32 to 85)** | **12 (8 to 17)** | **3494.05 (2147.71 to 5723.23)** | **1238.13 (856.98 to 1780.91)** | **-3.2 (-3.34 to -3.05)** |
| **Puerto Rico** | **0.824544** | **High SDI** | **14 (12 to 16)** | **2 (2 to 3)** | **4.34 (3.8 to 4.99)** | **2.13 (1.69 to 2.72)** | **-3.45 (-4.64 to -2.24)** | **1525 (1324 to 1760)** | **267 (215 to 330)** | **477.75 (414.84 to 551.23)** | **253.69 (204.34 to 313.81)** | **-2.73 (-3.42 to -2.05)** |
| **Saint Kitts and Nevis** | **0.756333** | **High-middle SDI** | **3 (3 to 3)** | **0 (0 to 1)** | **62.5 (54.31 to 71.53)** | **11.63 (8.31 to 16.42)** | **-4.91 (-5.68 to -4.12)** | **266 (232 to 303)** | **33 (24 to 47)** | **5685.05 (4944.12 to 6473.54)** | **1084.2 (790.92 to 1526.24)** | **-4.84 (-5.59 to -4.09)** |
| **San Marino** | **0.887884** | **High SDI** | **0 (0 to 0)** | **0 (0 to 0)** | **0.36 (0.18 to 0.53)** | **0.11 (0.06 to 0.2)** | **-3.27 (-3.53 to -3.01)** | **2 (1 to 3)** | **2 (1 to 3)** | **182.12 (119.46 to 266.45)** | **158.94 (96.52 to 246.66)** | **0.07 (-0.38 to 0.53)** |
| **Tokelau** | **0.687018** | **Middle SDI** | **0 (0 to 0)** | **0 (0 to 0)** | **49.77 (26.42 to 86.83)** | **88.89 (52.45 to 143.75)** | **-3.24 (-5.28 to -1.15)** | **9 (5 to 15)** | **8 (5 to 13)** | **4558.64 (2497.29 to 7859.64)** | **7977.78 (4709.82 to 12884.68)** | **-3.11 (-5.08 to -1.1)** |
| **Tuvalu** | **0.578627** | **Low-middle SDI** | **1 (1 to 2)** | **0 (0 to 0)** | **79.5 (45.07 to 121.58)** | **10.42 (6.67 to 15.3)** | **-5.79 (-6.38 to -5.2)** | **110 (64 to 166)** | **13 (8 to 18)** | **7278.92 (4225.27 to 11032.27)** | **987.74 (656.27 to 1426.57)** | **-5.71 (-6.28 to -5.14)** |
| **United States Virgin Islands** | **0.822988** | **High SDI** | **1 (1 to 1)** | **0 (0 to 0)** | **7.08 (4.71 to 10.49)** | **2.38 (1.35 to 3.62)** | **-3.41 (-3.73 to -3.08)** | **77 (54 to 111)** | **10 (6 to 14)** | **702.58 (492.64 to 1011.64)** | **250.63 (157.46 to 361.31)** | **-3.19 (-3.41 to -2.96)** |
| **South Sudan** | **0.278378** | **Low SDI** | **9555 (5897 to 13597)** | **7019 (4361 to 10426)** | **938.74 (579.41 to 1335.96)** | **449.27 (279.14 to 667.33)** | **-2.04 (-2.29 to -1.78)** | **855412 (531185 to 1214261)** | **626681 (389640 to 928777)** | **84044.74 (52189.19 to 119301.87)** | **40111.87 (24939.63 to 59448.03)** | **-2.05 (-2.29 to -1.8)** |
| **Sudan** | **0.542748** | **Low-middle SDI** | **15231 (8060 to 27603)** | **1593 (627 to 4446)** | **437.57 (231.55 to 792.99)** | **28.26 (11.12 to 78.84)** | **-8.64 (-9.11 to -8.16)** | **1377970 (735953 to 2478790)** | **151318 (66030 to 404811)** | **39586.74 (21142.68 to 71211.42)** | **2683.39 (1170.94 to 7178.68)** | **-8.48 (-8.96 to -8)** |
| **Georgia** | **0.847268** | **High SDI** | **197 (164 to 234)** | **2 (1 to 3)** | **41.97 (34.91 to 50.01)** | **0.86 (0.6 to 1.21)** | **-13.39 (-14.12 to -12.66)** | **18447 (15487 to 21824)** | **281 (214 to 372)** | **3937.03 (3305.2 to 4657.71)** | **115.6 (87.95 to 152.75)** | **-12.1 (-12.74 to -11.45)** |
| **Niger** | **0.425189** | **Low SDI** | **23016 (16896 to 29458)** | **10238 (6320 to 16433)** | **1364.31 (1001.57 to 1746.19)** | **200.89 (124.01 to 322.46)** | **-6.5 (-6.91 to -6.09)** | **2049782 (1511076 to 2621078)** | **915670 (569110 to 1463894)** | **121505.37 (89572.37 to 155370.18)** | **17967.2 (11167.03 to 28724.41)** | **-6.48 (-6.89 to -6.07)** |

**Abbreviations: ASR, age-standardized rate; DALYs, disability-adjusted life-years; SDI, sociodemographic index; GBD, Global Burden of Diseases, Injuries, and Risk Factors Study; EAPC, estimated annual percentage change; UIs, uncertainty intervals; CI, conﬁdence interval.**
