# Supplementary material for: Global trends, inequalities, and pathogen shifts in infectious diarrhea among children under five: a comprehensive analysis of the global burden of disease study 1990–2021
Source: Front Nutr. 2025 Nov 14;12:1679081. doi: 10.3389/fnut.2025.1679081 (PMC12661344; doi:10.3389/fnut.2025.1679081)
Supplement: Supplementary file 3 [file Table_3.docx]

**Table S3. The APC and AAPC in ASIR of infectious diarrhea in children under 5 years for both sexes from 1990 to 2021.**

| **Location** | **Segment** | **APC** | **P Value** | **AAPC (1990-2021)** | **P Value** |
| --- | --- | --- | --- | --- | --- |
| **Global** | **1990-1993** | **-2.326 (-2.814 to -1.8355)** | **<0.001** | **-3.6483 (-3.7749 to -3.5215)** | **<0.001** |
| **Global** | **1993-2004** | **-1.1798 (-1.2575 to -1.102)** | **<0.001** | **-3.6483 (-3.7749 to -3.5215)** | **<0.001** |
| **Global** | **2004-2011** | **-2.811 (-2.983 to -2.6386)** | **<0.001** | **-3.6483 (-3.7749 to -3.5215)** | **<0.001** |
| **Global** | **2011-2015** | **-6.563 (-7.071 to -6.0523)** | **<0.001** | **-3.6483 (-3.7749 to -3.5215)** | **<0.001** |
| **Global** | **2015-2019** | **-9.37 (-9.8796 to -8.8574)** | **<0.001** | **-3.6483 (-3.7749 to -3.5215)** | **<0.001** |
| **Global** | **2019-2021** | **-4.2234 (-5.3168 to -3.1174)** | **<0.001** | **-3.6483 (-3.7749 to -3.5215)** | **<0.001** |
| **High-middle SDI** | **1990-2004** | **-2.1697 (-2.2335 to -2.1059)** | **<0.001** | **-4.0585 (-4.2739 to -3.8425)** | **<0.001** |
| **High-middle SDI** | **2004-2012** | **-4.1935 (-4.3818 to -4.0048)** | **<0.001** | **-4.0585 (-4.2739 to -3.8425)** | **<0.001** |
| **High-middle SDI** | **2012-2015** | **-5.8666 (-7.3259 to -4.3843)** | **<0.001** | **-4.0585 (-4.2739 to -3.8425)** | **<0.001** |
| **High-middle SDI** | **2015-2018** | **-10.8019 (-12.2521 to -9.3276)** | **<0.001** | **-4.0585 (-4.2739 to -3.8425)** | **<0.001** |
| **High-middle SDI** | **2018-2021** | **-3.6082 (-4.4143 to -2.7953)** | **<0.001** | **-4.0585 (-4.2739 to -3.8425)** | **<0.001** |
| **High SDI** | **1990-1994** | **-2.9997 (-3.297 to -2.7016)** | **<0.001** | **-1.3191 (-1.4283 to -1.2099)** | **<0.001** |
| **High SDI** | **1994-2005** | **1.8156 (1.7423 to 1.8889)** | **<0.001** | **-1.3191 (-1.4283 to -1.2099)** | **<0.001** |
| **High SDI** | **2005-2011** | **-0.5315 (-0.7237 to -0.339)** | **<0.001** | **-1.3191 (-1.4283 to -1.2099)** | **<0.001** |
| **High SDI** | **2011-2015** | **-2.8032 (-3.227 to -2.3775)** | **<0.001** | **-1.3191 (-1.4283 to -1.2099)** | **<0.001** |
| **High SDI** | **2015-2019** | **-7.3813 (-7.812 to -6.9486)** | **<0.001** | **-1.3191 (-1.4283 to -1.2099)** | **<0.001** |
| **High SDI** | **2019-2021** | **-1.7509 (-2.7167 to -0.7754)** | **0.001705** | **-1.3191 (-1.4283 to -1.2099)** | **<0.001** |
| **Low-middle SDI** | **1990-1993** | **-3.0998 (-3.5605 to -2.6369)** | **<0.001** | **-4.0754 (-4.2055 to -3.9451)** | **<0.001** |
| **Low-middle SDI** | **1993-2006** | **-1.8856 (-1.9442 to -1.827)** | **<0.001** | **-4.0754 (-4.2055 to -3.9451)** | **<0.001** |
| **Low-middle SDI** | **2006-2011** | **-3.3738 (-3.698 to -3.0485)** | **<0.001** | **-4.0754 (-4.2055 to -3.9451)** | **<0.001** |
| **Low-middle SDI** | **2011-2015** | **-6.7915 (-7.319 to -6.261)** | **<0.001** | **-4.0754 (-4.2055 to -3.9451)** | **<0.001** |
| **Low-middle SDI** | **2015-2019** | **-9.0653 (-9.5886 to -8.5389)** | **<0.001** | **-4.0754 (-4.2055 to -3.9451)** | **<0.001** |
| **Low-middle SDI** | **2019-2021** | **-5.5809 (-6.6284 to -4.5217)** | **<0.001** | **-4.0754 (-4.2055 to -3.9451)** | **<0.001** |
| **Low SDI** | **1990-2002** | **-1.2108 (-1.2672 to -1.1543)** | **<0.001** | **-3.9421 (-4.1438 to -3.7399)** | **<0.001** |
| **Low SDI** | **2002-2010** | **-1.6234 (-1.7646 to -1.4821)** | **<0.001** | **-3.9421 (-4.1438 to -3.7399)** | **<0.001** |
| **Low SDI** | **2010-2013** | **-5.3231 (-6.4198 to -4.2136)** | **<0.001** | **-3.9421 (-4.1438 to -3.7399)** | **<0.001** |
| **Low SDI** | **2013-2016** | **-8.4336 (-9.5841 to -7.2685)** | **<0.001** | **-3.9421 (-4.1438 to -3.7399)** | **<0.001** |
| **Low SDI** | **2016-2019** | **-11.9982 (-13.1233 to -10.8586)** | **<0.001** | **-3.9421 (-4.1438 to -3.7399)** | **<0.001** |
| **Low SDI** | **2019-2021** | **-7.5867 (-8.7941 to -6.3633)** | **<0.001** | **-3.9421 (-4.1438 to -3.7399)** | **<0.001** |
| **Middle SDI** | **1990-1993** | **-3.2627 (-3.9691 to -2.5512)** | **<0.001** | **-4.0268 (-4.1894 to -3.864)** | **<0.001** |
| **Middle SDI** | **1993-2002** | **-2.3547 (-2.5176 to -2.1916)** | **<0.001** | **-4.0268 (-4.1894 to -3.864)** | **<0.001** |
| **Middle SDI** | **2002-2006** | **-3.1971 (-3.9533 to -2.435)** | **<0.001** | **-4.0268 (-4.1894 to -3.864)** | **<0.001** |
| **Middle SDI** | **2006-2013** | **-5.2881 (-5.5365 to -5.039)** | **<0.001** | **-4.0268 (-4.1894 to -3.864)** | **<0.001** |
| **Middle SDI** | **2013-2018** | **-7.4266 (-7.8924 to -6.9584)** | **<0.001** | **-4.0268 (-4.1894 to -3.864)** | **<0.001** |
| **Middle SDI** | **2018-2021** | **-2.1123 (-2.9081 to -1.3098)** | **<0.001** | **-4.0268 (-4.1894 to -3.864)** | **<0.001** |

**Abbreviations: ASIR, Age-standardized incidence rate; APC, Annual Percent Change; AAPC, Average Annual Percent Change; SDI, Sociodemographic Index.**
