# Supplementary material for: Global trends, inequalities, and pathogen shifts in infectious diarrhea among children under five: a comprehensive analysis of the global burden of disease study 1990–2021
Source: Front Nutr. 2025 Nov 14;12:1679081. doi: 10.3389/fnut.2025.1679081 (PMC12661344; doi:10.3389/fnut.2025.1679081)
Supplement: Supplementary file 4 [file Table_4.docx]

**Table S4. The APC and AAPC in ASPR of infectious diarrhea in children under 5 years for both sexes from 1990 to 2021.**

| **Location** | **Segment** | **APC** | **P Value** | **AAPC (1990-2021)** | **P Value** |
| --- | --- | --- | --- | --- | --- |
| **Global** | **1990-1993** | **-2.5337 (-3.0806 to -1.9838)** | **<0.001** | **-3.9804 (-4.1263 to -3.8343)** | **<0.001** |
| **Global** | **1993-2004** | **-1.5431 (-1.6275 to -1.4585)** | **<0.001** | **-3.9804 (-4.1263 to -3.8343)** | **<0.001** |
| **Global** | **2004-2011** | **-3.1378 (-3.3258 to -2.9495)** | **<0.001** | **-3.9804 (-4.1263 to -3.8343)** | **<0.001** |
| **Global** | **2011-2015** | **-7.1553 (-7.7297 to -6.5774)** | **<0.001** | **-3.9804 (-4.1263 to -3.8343)** | **<0.001** |
| **Global** | **2015-2019** | **-9.5885 (-10.1934 to -8.9795)** | **<0.001** | **-3.9804 (-4.1263 to -3.8343)** | **<0.001** |
| **Global** | **2019-2021** | **-4.2939 (-5.5809 to -2.9894)** | **<0.001** | **-3.9804 (-4.1263 to -3.8343)** | **<0.001** |
| **High-middle SDI** | **1990-1997** | **-2.3852 (-2.5378 to -2.2324)** | **<0.001** | **-4.315 (-4.4964 to -4.1333)** | **<0.001** |
| **High-middle SDI** | **1997-2004** | **-2.8375 (-3.03 to -2.6446)** | **<0.001** | **-4.315 (-4.4964 to -4.1333)** | **<0.001** |
| **High-middle SDI** | **2004-2012** | **-4.316 (-4.4707 to -4.161)** | **<0.001** | **-4.315 (-4.4964 to -4.1333)** | **<0.001** |
| **High-middle SDI** | **2012-2015** | **-6.1614 (-7.3647 to -4.9425)** | **<0.001** | **-4.315 (-4.4964 to -4.1333)** | **<0.001** |
| **High-middle SDI** | **2015-2018** | **-10.9124 (-12.1179 to -9.6903)** | **<0.001** | **-4.315 (-4.4964 to -4.1333)** | **<0.001** |
| **High-middle SDI** | **2018-2021** | **-3.4878 (-4.1608 to -2.8101)** | **<0.001** | **-4.315 (-4.4964 to -4.1333)** | **<0.001** |
| **High SDI** | **1990-1994** | **-3.0131 (-3.3132 to -2.7121)** | **<0.001** | **-1.3867 (-1.5081 to -1.2652)** | **<0.001** |
| **High SDI** | **1994-2005** | **1.7376 (1.6658 to 1.8094)** | **<0.001** | **-1.3867 (-1.5081 to -1.2652)** | **<0.001** |
| **High SDI** | **2005-2012** | **-0.7643 (-0.9091 to -0.6193)** | **<0.001** | **-1.3867 (-1.5081 to -1.2652)** | **<0.001** |
| **High SDI** | **2012-2015** | **-3.4077 (-4.2462 to -2.5619)** | **<0.001** | **-1.3867 (-1.5081 to -1.2652)** | **<0.001** |
| **High SDI** | **2015-2019** | **-7.3708 (-7.7968 to -6.9428)** | **<0.001** | **-1.3867 (-1.5081 to -1.2652)** | **<0.001** |
| **High SDI** | **2019-2021** | **-1.785 (-2.7388 to -0.8218)** | **0.001327** | **-1.3867 (-1.5081 to -1.2652)** | **<0.001** |
| **Low-middle SDI** | **1990-1993** | **-3.3588 (-3.8466 to -2.8686)** | **<0.001** | **-4.3751 (-4.5108 to -4.2392)** | **<0.001** |
| **Low-middle SDI** | **1993-2006** | **-2.2011 (-2.2592 to -2.1429)** | **<0.001** | **-4.3751 (-4.5108 to -4.2392)** | **<0.001** |
| **Low-middle SDI** | **2006-2011** | **-3.6805 (-4.0078 to -3.3521)** | **<0.001** | **-4.3751 (-4.5108 to -4.2392)** | **<0.001** |
| **Low-middle SDI** | **2011-2015** | **-7.4525 (-7.9997 to -6.9021)** | **<0.001** | **-4.3751 (-4.5108 to -4.2392)** | **<0.001** |
| **Low-middle SDI** | **2015-2019** | **-9.1439 (-9.6942 to -8.5903)** | **<0.001** | **-4.3751 (-4.5108 to -4.2392)** | **<0.001** |
| **Low-middle SDI** | **2019-2021** | **-5.548 (-6.6585 to -4.4244)** | **<0.001** | **-4.3751 (-4.5108 to -4.2392)** | **<0.001** |
| **Low SDI** | **1990-2001** | **-1.5854 (-1.6305 to -1.5403)** | **<0.001** | **-4.4269 (-4.5661 to -4.2876)** | **<0.001** |
| **Low SDI** | **2001-2010** | **-2.1407 (-2.2164 to -2.0649)** | **<0.001** | **-4.4269 (-4.5661 to -4.2876)** | **<0.001** |
| **Low SDI** | **2010-2013** | **-5.9934 (-6.7092 to -5.2722)** | **<0.001** | **-4.4269 (-4.5661 to -4.2876)** | **<0.001** |
| **Low SDI** | **2013-2016** | **-9.1613 (-9.9496 to -8.3661)** | **<0.001** | **-4.4269 (-4.5661 to -4.2876)** | **<0.001** |
| **Low SDI** | **2016-2019** | **-12.3853 (-13.1903 to -11.5729)** | **<0.001** | **-4.4269 (-4.5661 to -4.2876)** | **<0.001** |
| **Low SDI** | **2019-2021** | **-7.8166 (-8.6914 to -6.9334)** | **<0.001** | **-4.4269 (-4.5661 to -4.2876)** | **<0.001** |
| **Middle SDI** | **1990-1993** | **-3.6672 (-4.328 to -3.0018)** | **<0.001** | **-4.3186 (-4.4717 to -4.1652)** | **<0.001** |
| **Middle SDI** | **1993-2002** | **-2.7619 (-2.9098 to -2.6138)** | **<0.001** | **-4.3186 (-4.4717 to -4.1652)** | **<0.001** |
| **Middle SDI** | **2002-2006** | **-3.4963 (-4.1989 to -2.7886)** | **<0.001** | **-4.3186 (-4.4717 to -4.1652)** | **<0.001** |
| **Middle SDI** | **2006-2013** | **-5.4896 (-5.7278 to -5.2509)** | **<0.001** | **-4.3186 (-4.4717 to -4.1652)** | **<0.001** |
| **Middle SDI** | **2013-2018** | **-7.697 (-8.1449 to -7.247)** | **<0.001** | **-4.3186 (-4.4717 to -4.1652)** | **<0.001** |
| **Middle SDI** | **2018-2021** | **-2.1869 (-2.9442 to -1.4238)** | **<0.001** | **-4.3186 (-4.4717 to -4.1652)** | **<0.001** |

**Abbreviations: ASPR, Age-standardized prevalence rate; APC, Annual Percent Change; AAPC, Average Annual Percent Change; SDI, Sociodemographic Index.**
