# Supplementary material for: Global trends, inequalities, and pathogen shifts in infectious diarrhea among children under five: a comprehensive analysis of the global burden of disease study 1990–2021
Source: Front Nutr. 2025 Nov 14;12:1679081. doi: 10.3389/fnut.2025.1679081 (PMC12661344; doi:10.3389/fnut.2025.1679081)
Supplement: Supplementary file 5 [file Table_5.docx]

**Table S5. The APC and AAPC in ASMR of infectious diarrhea in children under 5 years for both sexes from 1990 to 2021.**

| **Location** | **Segment** | **APC** | **P Value** | **AAPC (1990-2021)** | **P Value** |
| --- | --- | --- | --- | --- | --- |
| **Global** | **1990-1997** | **-3.1846 (-3.3759 to -2.993)** | **<0.001** | **-5.142 (-5.249 to -5.0348)** | **<0.001** |
| **Global** | **1997-2007** | **-4.0051 (-4.129 to -3.8811)** | **<0.001** | **-5.142 (-5.249 to -5.0348)** | **<0.001** |
| **Global** | **2007-2011** | **-5.7415 (-6.4074 to -5.0708)** | **<0.001** | **-5.142 (-5.249 to -5.0348)** | **<0.001** |
| **Global** | **2011-2021** | **-7.3613 (-7.4976 to -7.2248)** | **<0.001** | **-5.142 (-5.249 to -5.0348)** | **<0.001** |
| **High-middle SDI** | **1990-1999** | **-7.6428 (-7.7979 to -7.4875)** | **<0.001** | **-8.37 (-8.5936 to -8.1459)** | **<0.001** |
| **High-middle SDI** | **1999-2007** | **-10.5405 (-10.7447 to -10.3358)** | **<0.001** | **-8.37 (-8.5936 to -8.1459)** | **<0.001** |
| **High-middle SDI** | **2007-2011** | **-9.241 (-10.0208 to -8.4545)** | **<0.001** | **-8.37 (-8.5936 to -8.1459)** | **<0.001** |
| **High-middle SDI** | **2011-2014** | **-6.0843 (-7.5986 to -4.5452)** | **<0.001** | **-8.37 (-8.5936 to -8.1459)** | **<0.001** |
| **High-middle SDI** | **2014-2019** | **-8.1855 (-8.7061 to -7.662)** | **<0.001** | **-8.37 (-8.5936 to -8.1459)** | **<0.001** |
| **High-middle SDI** | **2019-2021** | **-4.8756 (-6.8957 to -2.8116)** | **<0.001** | **-8.37 (-8.5936 to -8.1459)** | **<0.001** |
| **High SDI** | **1990-1999** | **-6.2883 (-6.863 to -5.7102)** | **<0.001** | **-4.5865 (-4.9596 to -4.2121)** | **<0.001** |
| **High SDI** | **1999-2007** | **-2.1633 (-2.6197 to -1.7048)** | **<0.001** | **-4.5865 (-4.9596 to -4.2121)** | **<0.001** |
| **High SDI** | **2007-2010** | **-6.4998 (-8.7692 to -4.1739)** | **<0.001** | **-4.5865 (-4.9596 to -4.2121)** | **<0.001** |
| **High SDI** | **2010-2014** | **-4.1956 (-5.3277 to -3.0499)** | **<0.001** | **-4.5865 (-4.9596 to -4.2121)** | **<0.001** |
| **High SDI** | **2014-2018** | **-2.1822 (-3.4235 to -0.925)** | **0.002217** | **-4.5865 (-4.9596 to -4.2121)** | **<0.001** |
| **High SDI** | **2018-2021** | **-7.5238 (-9.1033 to -5.9169)** | **<0.001** | **-4.5865 (-4.9596 to -4.2121)** | **<0.001** |
| **Low-middle SDI** | **1990-1995** | **-4.7004 (-5.1741 to -4.2242)** | **<0.001** | **-7.084 (-7.2747 to -6.893)** | **<0.001** |
| **Low-middle SDI** | **1995-2002** | **-6.2591 (-6.6098 to -5.9071)** | **<0.001** | **-7.084 (-7.2747 to -6.893)** | **<0.001** |
| **Low-middle SDI** | **2002-2009** | **-5.6009 (-5.9438 to -5.2567)** | **<0.001** | **-7.084 (-7.2747 to -6.893)** | **<0.001** |
| **Low-middle SDI** | **2009-2017** | **-8.6585 (-8.9747 to -8.3412)** | **<0.001** | **-7.084 (-7.2747 to -6.893)** | **<0.001** |
| **Low-middle SDI** | **2017-2021** | **-10.7866 (-11.7612 to -9.8012)** | **<0.001** | **-7.084 (-7.2747 to -6.893)** | **<0.001** |
| **Low SDI** | **1990-1994** | **-1.7658 (-2.4267 to -1.1005)** | **<0.001** | **-4.812 (-5.0224 to -4.6011)** | **<0.001** |
| **Low SDI** | **1994-2007** | **-3.7535 (-3.8608 to -3.6462)** | **<0.001** | **-4.812 (-5.0224 to -4.6011)** | **<0.001** |
| **Low SDI** | **2007-2010** | **-5.0704 (-6.7204 to -3.3913)** | **<0.001** | **-4.812 (-5.0224 to -4.6011)** | **<0.001** |
| **Low SDI** | **2010-2017** | **-6.5013 (-6.8205 to -6.181)** | **<0.001** | **-4.812 (-5.0224 to -4.6011)** | **<0.001** |
| **Low SDI** | **2017-2021** | **-8 (-8.7699 to -7.2237)** | **<0.001** | **-4.812 (-5.0224 to -4.6011)** | **<0.001** |
| **Middle SDI** | **1990-1997** | **-7.2177 (-7.591 to -6.8429)** | **<0.001** | **-7.2367 (-7.3706 to -7.1026)** | **<0.001** |
| **Middle SDI** | **1997-2005** | **-5.8321 (-6.2094 to -5.4533)** | **<0.001** | **-7.2367 (-7.3706 to -7.1026)** | **<0.001** |
| **Middle SDI** | **2005-2021** | **-7.9393 (-8.0538 to -7.8247)** | **<0.001** | **-7.2367 (-7.3706 to -7.1026)** | **<0.001** |

**Abbreviations: ASMR, Age-standardized mortality rate; APC, Annual Percent Change; AAPC, Average Annual Percent Change; SDI, Sociodemographic Index.**
