# Supplementary material for: Global trends, inequalities, and pathogen shifts in infectious diarrhea among children under five: a comprehensive analysis of the global burden of disease study 1990–2021
Source: Front Nutr. 2025 Nov 14;12:1679081. doi: 10.3389/fnut.2025.1679081 (PMC12661344; doi:10.3389/fnut.2025.1679081)
Supplement: Supplementary file 6 [file Table_6.docx]

**Table S6. The APC and AAPC in ASDR of infectious diarrhea in children under 5 years for both sexes from 1990 to 2021.**

| **Location** | **Segment** | **APC** | **P Value** | **AAPC (1990-2021)** | **P Value** |
| --- | --- | --- | --- | --- | --- |
| **Global** | **1990-1997** | **-3.1612 (-3.35 to -2.9721)** | **<0.001** | **-5.1242 (-5.2298 to -5.0185)** | **<0.001** |
| **Global** | **1997-2007** | **-3.9684 (-4.0919 to -3.8447)** | **<0.001** | **-5.1242 (-5.2298 to -5.0185)** | **<0.001** |
| **Global** | **2007-2011** | **-5.6887 (-6.3433 to -5.0296)** | **<0.001** | **-5.1242 (-5.2298 to -5.0185)** | **<0.001** |
| **Global** | **2011-2021** | **-7.3794 (-7.5144 to -7.2442)** | **<0.001** | **-5.1242 (-5.2298 to -5.0185)** | **<0.001** |
| **High-middle SDI** | **1990-1999** | **-7.2791 (-7.419 to -7.139)** | **<0.001** | **-7.9086 (-8.1022 to -7.7147)** | **<0.001** |
| **High-middle SDI** | **1999-2007** | **-9.6968 (-9.8751 to -9.5182)** | **<0.001** | **-7.9086 (-8.1022 to -7.7147)** | **<0.001** |
| **High-middle SDI** | **2007-2011** | **-8.3982 (-9.0638 to -7.7278)** | **<0.001** | **-7.9086 (-8.1022 to -7.7147)** | **<0.001** |
| **High-middle SDI** | **2011-2014** | **-5.9169 (-7.2597 to -4.5546)** | **<0.001** | **-7.9086 (-8.1022 to -7.7147)** | **<0.001** |
| **High-middle SDI** | **2014-2019** | **-8.3918 (-8.8437 to -7.9376)** | **<0.001** | **-7.9086 (-8.1022 to -7.7147)** | **<0.001** |
| **High-middle SDI** | **2019-2021** | **-4.1992 (-5.8923 to -2.4756)** | **<0.001** | **-7.9086 (-8.1022 to -7.7147)** | **<0.001** |
| **High SDI** | **1990-1995** | **-5.4472 (-5.9683 to -4.9232)** | **<0.001** | **-3.4085 (-3.602 to -3.2146)** | **<0.001** |
| **High SDI** | **1995-1999** | **-3.0491 (-4.07 to -2.0173)** | **<0.001** | **-3.4085 (-3.602 to -3.2146)** | **<0.001** |
| **High SDI** | **1999-2007** | **-0.846 (-1.133 to -0.5582)** | **<0.001** | **-3.4085 (-3.602 to -3.2146)** | **<0.001** |
| **High SDI** | **2007-2016** | **-3.4727 (-3.726 to -3.2188)** | **<0.001** | **-3.4085 (-3.602 to -3.2146)** | **<0.001** |
| **High SDI** | **2016-2021** | **-5.5419 (-6.0703 to -5.0105)** | **<0.001** | **-3.4085 (-3.602 to -3.2146)** | **<0.001** |
| **Low-middle SDI** | **1990-1993** | **-4.1023 (-5.3139 to -2.8752)** | **<0.001** | **-7.0277 (-7.2557 to -6.7991)** | **<0.001** |
| **Low-middle SDI** | **1993-2010** | **-5.879 (-5.9704 to -5.7875)** | **<0.001** | **-7.0277 (-7.2557 to -6.7991)** | **<0.001** |
| **Low-middle SDI** | **2010-2018** | **-8.9349 (-9.3049 to -8.5634)** | **<0.001** | **-7.0277 (-7.2557 to -6.7991)** | **<0.001** |
| **Low-middle SDI** | **2018-2021** | **-11.1444 (-12.9359 to -9.3159)** | **<0.001** | **-7.0277 (-7.2557 to -6.7991)** | **<0.001** |
| **Low SDI** | **1990-1994** | **-1.7564 (-2.413 to -1.0953)** | **<0.001** | **-4.8101 (-5.0193 to -4.6005)** | **<0.001** |
| **Low SDI** | **1994-2007** | **-3.732 (-3.8384 to -3.6255)** | **<0.001** | **-4.8101 (-5.0193 to -4.6005)** | **<0.001** |
| **Low SDI** | **2007-2010** | **-5.0214 (-6.6649 to -3.349)** | **<0.001** | **-4.8101 (-5.0193 to -4.6005)** | **<0.001** |
| **Low SDI** | **2010-2017** | **-6.5218 (-6.8381 to -6.2043)** | **<0.001** | **-4.8101 (-5.0193 to -4.6005)** | **<0.001** |
| **Low SDI** | **2017-2021** | **-8.0619 (-8.8247 to -7.2927)** | **<0.001** | **-4.8101 (-5.0193 to -4.6005)** | **<0.001** |
| **Middle SDI** | **1990-1997** | **-7.0941 (-7.4566 to -6.7302)** | **<0.001** | **-7.1251 (-7.2551 to -6.9949)** | **<0.001** |
| **Middle SDI** | **1997-2005** | **-5.7061 (-6.0756 to -5.3351)** | **<0.001** | **-7.1251 (-7.2551 to -6.9949)** | **<0.001** |
| **Middle SDI** | **2005-2021** | **-7.84 (-7.9487 to -7.7312)** | **<0.001** | **-7.1251 (-7.2551 to -6.9949)** | **<0.001** |

**Abbreviations: ASDR, Age-standardized disability-adjusted life-year rate; APC, Annual Percent Change; AAPC, Average Annual Percent Change; SDI, Sociodemographic Index.**
