# Supplementary material for: Global trends, inequalities, and pathogen shifts in infectious diarrhea among children under five: a comprehensive analysis of the global burden of disease study 1990–2021
Source: Front Nutr. 2025 Nov 14;12:1679081. doi: 10.3389/fnut.2025.1679081 (PMC12661344; doi:10.3389/fnut.2025.1679081)
Supplement: Supplementary file 8 [file Table_8.docx]

**Table S8. The EAPC of ASDR of infectious diarrhea in children under 5 years of age in 2021 by Pathogen.**

| **location** | **Cryptosporidium** | **Adenovirus** | **Enterotoxigenic E coli** | **Aeromonas** | **Shigella** | **Non-typhoidal Salmonella** | **Enteropathogenic E coli** | **Entamoeba** | **Norovirus** | **Cholera** | **Campylobacter** | **Rotavirus** | **Clostridium difficile** |
| --- | --- | --- | --- | --- | --- | --- | --- | --- | --- | --- | --- | --- | --- |
| **Global** | **-4.06 (-4.53 to -3.6)** | **-4.96 (-5.18 to -4.74)** | **-4.68 (-5.02 to -4.34)** | **-5.04 (-5.29 to -4.79)** | **-4.22 (-4.61 to -3.82)** | **-4.66 (-4.94 to -4.38)** | **-4.24 (-4.66 to -3.82)** | **-5.12 (-5.36 to -4.88)** | **-4.97 (-5.21 to -4.72)** | **-5.71 (-6.42 to -4.99)** | **-5.66 (-5.96 to -5.35)** | **-5.12 (-5.53 to -4.7)** | **0.17 (0.06 to 0.27)** |
| **Low SDI** | **-4.41 (-4.8 to -4.02)** | **-4.59 (-4.83 to -4.35)** | **-4.67 (-4.92 to -4.41)** | **-4.47 (-4.77 to -4.18)** | **-4.4 (-4.73 to -4.07)** | **-4.43 (-4.72 to -4.13)** | **-4.34 (-4.68 to -3.99)** | **-4.99 (-5.23 to -4.76)** | **-4.65 (-4.93 to -4.37)** | **-7.33 (-8.12 to -6.54)** | **-5.21 (-5.47 to -4.96)** | **-4.74 (-5.15 to -4.33)** | **0.66 (0.57 to 0.75)** |
| **Low-middle SDI** | **-5.98 (-6.43 to -5.53)** | **-7.01 (-7.15 to -6.87)** | **-6.65 (-7.01 to -6.29)** | **-6.94 (-7.14 to -6.74)** | **-6.25 (-6.63 to -5.87)** | **-6.39 (-6.66 to -6.12)** | **-6.3 (-6.69 to -5.9)** | **-6.71 (-6.95 to -6.48)** | **-6.78 (-6.96 to -6.59)** | **-5.72 (-5.85 to -5.59)** | **-7.19 (-7.48 to -6.9)** | **-7.11 (-7.57 to -6.65)** | **0.72 (0.64 to 0.8)** |
| **Middle SDI** | **-5.93 (-6.3 to -5.56)** | **-6.41 (-6.55 to -6.27)** | **-7.09 (-7.38 to -6.81)** | **-7.25 (-7.33 to -7.16)** | **-5.94 (-6.27 to -5.61)** | **-6.83 (-6.93 to -6.73)** | **-5.62 (-6.03 to -5.22)** | **-7.32 (-7.43 to -7.22)** | **-7.03 (-7.09 to -6.98)** | **-3.59 (-3.96 to -3.22)** | **-6.81 (-7.07 to -6.56)** | **-8 (-8.16 to -7.83)** | **0.48 (0.37 to 0.6)** |
| **High-middle SDI** | **-9.37 (-9.58 to -9.16)** | **-7.39 (-7.59 to -7.2)** | **-7.31 (-7.61 to -7.02)** | **-9.38 (-9.61 to -9.15)** | **-8.37 (-8.49 to -8.25)** | **-8.45 (-8.6 to -8.3)** | **-8.49 (-8.66 to -8.32)** | **-9.95 (-10.22 to -9.68)** | **-8.6 (-8.82 to -8.39)** | **-4.29 (-4.61 to -3.97)** | **-7.51 (-7.71 to -7.31)** | **-9.05 (-9.22 to -8.89)** | **0.84 (0.6 to 1.07)** |
| **High SDI** | **-6.3 (-6.51 to -6.08)** | **-3.8 (-4 to -3.6)** | **-4.82 (-5.02 to -4.62)** | **-5.08 (-5.3 to -4.85)** | **-4.28 (-4.48 to -4.08)** | **-3.87 (-4.09 to -3.65)** | **-3.36 (-3.49 to -3.23)** | **-4.34 (-4.62 to -4.06)** | **-2.41 (-2.65 to -2.18)** | **-3.01 (-3.71 to -2.31)** | **-2.51 (-2.8 to -2.21)** | **-3.87 (-4.15 to -3.6)** | **0.22 (-0.02 to 0.47)** |
| **Western Sub-Saharan Africa** | **-4.58 (-5.1 to -4.06)** | **-4.28 (-4.68 to -3.88)** | **-4.56 (-4.93 to -4.19)** | **-4.51 (-4.91 to -4.1)** | **-4.24 (-4.73 to -3.74)** | **-4.36 (-4.78 to -3.94)** | **-4.17 (-4.64 to -3.7)** | **-4.35 (-4.74 to -3.95)** | **-4.52 (-4.94 to -4.11)** | **-7.98 (-9.12 to -6.83)** | **-4.46 (-4.84 to -4.08)** | **-4.42 (-4.88 to -3.96)** | **0.81 (0.73 to 0.9)** |
| **Southern Sub-Saharan Africa** | **-3.83 (-4.27 to -3.38)** | **-3.59 (-4.09 to -3.09)** | **-3.33 (-3.82 to -2.85)** | **-3.14 (-3.52 to -2.76)** | **-3.36 (-3.89 to -2.82)** | **-3.41 (-3.87 to -2.95)** | **-3.73 (-4.29 to -3.16)** | **-3.53 (-3.98 to -3.08)** | **-3.11 (-3.47 to -2.75)** | **-2.08 (-2.91 to -1.24)** | **-3.91 (-4.47 to -3.34)** | **-5.1 (-5.82 to -4.37)** | **0.6 (0.3 to 0.9)** |
| **Eastern Sub-Saharan Africa** | **-5.58 (-5.74 to -5.41)** | **-5.5 (-5.67 to -5.33)** | **-5.38 (-5.56 to -5.2)** | **-5.46 (-5.6 to -5.32)** | **-5.52 (-5.69 to -5.35)** | **-5.52 (-5.69 to -5.35)** | **-5.45 (-5.65 to -5.26)** | **-5.62 (-5.75 to -5.49)** | **-5.33 (-5.52 to -5.14)** | **-8.1 (-8.55 to -7.64)** | **-5.43 (-5.6 to -5.25)** | **-5.62 (-5.94 to -5.29)** | **0.69 (0.6 to 0.78)** |
| **Central Sub-Saharan Africa** | **-7.08 (-8.03 to -6.13)** | **-6.75 (-7.75 to -5.74)** | **-6.71 (-7.66 to -5.74)** | **-6.8 (-7.76 to -5.84)** | **-6.22 (-7.14 to -5.29)** | **-6.34 (-7.26 to -5.42)** | **-6.66 (-7.61 to -5.7)** | **-6.63 (-7.58 to -5.67)** | **-6.88 (-7.87 to -5.88)** | **-6.8 (-7.44 to -6.16)** | **-6.85 (-7.79 to -5.89)** | **-6.94 (-8.01 to -5.85)** | **0.53 (0.39 to 0.67)** |
| **Southern Latin America** | **-5.69 (-6 to -5.37)** | **-5.42 (-5.76 to -5.07)** | **-5.55 (-5.9 to -5.19)** | **-5.32 (-5.64 to -5)** | **-5.6 (-5.92 to -5.28)** | **-5.66 (-5.98 to -5.33)** | **-5.73 (-6.03 to -5.43)** | **-5.64 (-5.95 to -5.32)** | **-5.18 (-5.52 to -4.83)** |  | **-5.71 (-5.99 to -5.42)** | **-7.08 (-7.46 to -6.7)** | **1.49 (1.22 to 1.77)** |
| **Central Latin America** | **-7.88 (-8.32 to -7.44)** | **-7.52 (-7.94 to -7.09)** | **-7.58 (-7.98 to -7.19)** | **-7.37 (-7.77 to -6.97)** | **-7.14 (-7.59 to -6.69)** | **-8.07 (-8.5 to -7.64)** | **-7.55 (-7.96 to -7.13)** | **-7.89 (-8.29 to -7.48)** | **-6.78 (-7.1 to -6.45)** |  | **-8.26 (-8.71 to -7.81)** | **-9.75 (-10.48 to -9.01)** | **0.62 (0.43 to 0.8)** |
| **Tropical Latin America** | **-11.33 (-11.53 to -11.14)** | **-10.2 (-10.42 to -9.98)** | **-11.37 (-11.57 to -11.17)** | **-11.09 (-11.28 to -10.89)** | **-11.08 (-11.42 to -10.75)** | **-11.46 (-11.73 to -11.19)** | **-11.66 (-11.87 to -11.46)** | **-11.58 (-11.77 to -11.4)** | **-10.71 (-10.87 to -10.54)** |  | **-11.21 (-11.45 to -10.97)** | **-13.19 (-13.71 to -12.66)** | **0.71 (0.33 to 1.09)** |
| **Andean Latin America** | **-9.09 (-9.32 to -8.85)** | **-8.76 (-8.99 to -8.53)** | **-8.69 (-8.95 to -8.42)** | **-9.03 (-9.25 to -8.81)** | **-9.05 (-9.27 to -8.83)** | **-8.82 (-9.04 to -8.61)** | **-9.4 (-9.7 to -9.1)** | **-9.05 (-9.28 to -8.81)** | **-9.1 (-9.33 to -8.87)** |  | **-9.58 (-9.86 to -9.29)** | **-11.35 (-11.94 to -10.76)** | **0.85 (0.65 to 1.04)** |
| **Caribbean** | **-3.1 (-3.58 to -2.62)** | **-3.11 (-3.6 to -2.63)** | **-3.14 (-3.61 to -2.65)** | **-3.1 (-3.57 to -2.63)** | **-3.36 (-3.8 to -2.91)** | **-3.6 (-4.07 to -3.14)** | **-3.12 (-3.6 to -2.63)** | **-3.09 (-3.57 to -2.61)** | **-3.09 (-3.56 to -2.62)** |  | **-3.17 (-3.64 to -2.69)** | **-4.34 (-4.86 to -3.82)** | **1.33 (1.08 to 1.59)** |
| **North Africa and Middle East** | **-7.12 (-7.32 to -6.92)** | **-7.11 (-7.35 to -6.86)** | **-7.04 (-7.3 to -6.78)** | **-7.08 (-7.35 to -6.82)** | **-7.13 (-7.45 to -6.79)** | **-7.15 (-7.43 to -6.88)** | **-7.08 (-7.3 to -6.86)** | **-7.13 (-7.42 to -6.84)** | **-7 (-7.21 to -6.78)** | **-6.06 (-6.47 to -5.64)** | **-6.88 (-7.03 to -6.74)** | **-7.51 (-7.87 to -7.15)** | **0.76 (0.66 to 0.85)** |
| **Oceania** | **-1.68 (-1.92 to -1.44)** | **-1.86 (-2.07 to -1.65)** | **-1.79 (-2.05 to -1.54)** | **-1.79 (-2 to -1.58)** | **-1.71 (-1.92 to -1.5)** | **-1.74 (-1.95 to -1.54)** | **-1.87 (-2.09 to -1.65)** | **-1.67 (-1.89 to -1.45)** | **-1.97 (-2.18 to -1.77)** | **-3.18 (-3.41 to -2.95)** | **-1.65 (-1.84 to -1.46)** | **-1.73 (-1.94 to -1.52)** | **0.22 (0.1 to 0.34)** |
| **Southeast Asia** | **-7.7 (-7.8 to -7.61)** | **-7.53 (-7.66 to -7.4)** | **-8.42 (-8.69 to -8.16)** | **-7.64 (-7.77 to -7.52)** | **-7.6 (-7.72 to -7.48)** | **-7.53 (-7.64 to -7.42)** | **-7.58 (-7.68 to -7.49)** | **-7.64 (-7.74 to -7.54)** | **-7.48 (-7.61 to -7.35)** | **-6.06 (-6.24 to -5.88)** | **-7.39 (-7.47 to -7.31)** | **-8.05 (-8.23 to -7.88)** | **0.3 (0.22 to 0.37)** |
| **South Asia** | **-6.86 (-7.26 to -6.46)** | **-7.26 (-7.48 to -7.04)** | **-6.96 (-7.38 to -6.53)** | **-6.99 (-7.17 to -6.81)** | **-6.89 (-7.23 to -6.54)** | **-7.1 (-7.35 to -6.85)** | **-6.93 (-7.29 to -6.58)** | **-7.26 (-7.5 to -7.02)** | **-7.26 (-7.43 to -7.1)** | **-4.99 (-5.55 to -4.42)** | **-7.1 (-7.43 to -6.77)** | **-7.58 (-8.15 to -7)** | **0.38 (0.28 to 0.48)** |
| **Central Asia** | **-6.81 (-7.09 to -6.53)** | **-8.13 (-8.43 to -7.82)** | **-7.92 (-8.22 to -7.62)** | **-7.8 (-8.12 to -7.47)** | **-6.89 (-7.18 to -6.6)** | **-7.71 (-8.01 to -7.41)** | **-7.7 (-7.98 to -7.42)** | **-7.7 (-7.99 to -7.4)** | **-8.44 (-8.79 to -8.09)** | **-4.6 (-5.08 to -4.12)** | **-8.03 (-8.32 to -7.74)** | **-8.45 (-8.9 to -8)** | **0.65 (0.47 to 0.84)** |
| **East Asia** | **-14.25 (-14.77 to -13.72)** | **-13.42 (-13.95 to -12.88)** | **-13.7 (-14.15 to -13.25)** | **-13.52 (-13.96 to -13.08)** | **-13.28 (-13.74 to -12.82)** | **-13.43 (-13.87 to -12.98)** | **-13.73 (-14.2 to -13.25)** | **-13.48 (-13.94 to -13.02)** | **-13.38 (-13.85 to -12.9)** | **-7.87 (-8.53 to -7.2)** | **-13.41 (-13.99 to -12.82)** | **-13.48 (-13.91 to -13.04)** | **0.58 (0.51 to 0.66)** |
| **Eastern Europe** | **-7.4 (-7.95 to -6.84)** | **-7.57 (-8.1 to -7.03)** | **-7.41 (-7.93 to -6.89)** | **-7.39 (-7.9 to -6.87)** | **-7.35 (-7.86 to -6.84)** | **-7.67 (-8.21 to -7.13)** | **-7.63 (-8.17 to -7.08)** | **-7.73 (-8.28 to -7.18)** | **-6.83 (-7.3 to -6.36)** |  | **-7.34 (-7.86 to -6.82)** | **-8.67 (-9.4 to -7.95)** | **0.98 (0.67 to 1.3)** |
| **Central Europe** | **-2.62 (-3.74 to -1.49)** | **-2.98 (-4.01 to -1.94)** | **-2.65 (-3.71 to -1.59)** | **-2.35 (-3.45 to -1.24)** | **-2.54 (-3.62 to -1.46)** | **-2.58 (-3.68 to -1.47)** | **-2.66 (-3.75 to -1.55)** | **-2.58 (-3.71 to -1.45)** | **-2.32 (-3.3 to -1.34)** |  | **-2.69 (-3.71 to -1.65)** | **-2.42 (-3.54 to -1.3)** | **0.55 (0.22 to 0.89)** |
| **Western Europe** | **0.62 (-0.12 to 1.36)** | **0.38 (-0.35 to 1.11)** | **0.18 (-0.49 to 0.85)** | **0.27 (-0.39 to 0.94)** | **0.33 (-0.32 to 0.98)** | **0.38 (-0.32 to 1.1)** | **0.43 (-0.2 to 1.07)** | **0.32 (-0.36 to 1)** | **0.02 (-0.62 to 0.66)** |  | **0.36 (-0.37 to 1.08)** | **-0.81 (-1.59 to -0.02)** | **1.01 (0.6 to 1.43)** |
| **Australasia** | **-0.46 (-1.45 to 0.54)** | **-0.51 (-1.45 to 0.43)** | **-0.76 (-1.61 to 0.09)** | **-0.76 (-1.68 to 0.16)** | **-0.72 (-1.6 to 0.16)** | **-0.7 (-1.63 to 0.24)** | **-0.63 (-1.53 to 0.28)** | **-0.61 (-1.57 to 0.36)** | **-0.91 (-1.69 to -0.13)** |  | **-0.63 (-1.47 to 0.21)** | **-4.55 (-5.65 to -3.44)** | **0.82 (0.2 to 1.43)** |
| **High-income Asia Pacific** | **-1.06 (-1.42 to -0.71)** | **-0.07 (-0.4 to 0.27)** | **-0.42 (-0.78 to -0.06)** | **-0.75 (-1.12 to -0.38)** | **-0.06 (-0.45 to 0.35)** | **-0.38 (-0.78 to 0.02)** | **-0.8 (-1.15 to -0.45)** | **-0.89 (-1.25 to -0.53)** | **0.35 (0.05 to 0.66)** |  | **0.38 (-0.03 to 0.8)** | **-1.39 (-1.69 to -1.09)** | **-0.54 (-0.66 to -0.41)** |
| **High-income North America** | **-2.34 (-2.87 to -1.8)** | **-3.5 (-4.02 to -2.97)** | **-3.03 (-3.51 to -2.54)** | **-2.47 (-2.97 to -1.97)** | **-2.16 (-2.62 to -1.7)** | **-2.11 (-2.63 to -1.58)** | **-2.69 (-3.09 to -2.28)** | **-2.55 (-3.08 to -2.02)** | **-3.35 (-3.82 to -2.88)** |  | **-3.14 (-3.59 to -2.68)** | **-4.32 (-5.12 to -3.51)** | **0.19 (-0.08 to 0.46)** |

**Abbreviations: ASDR, Age-standardized disability-adjusted life-year rate; SDI, sociodemographic index; GBD, Global Burden of Diseases, Injuries, and Risk Factors Study; EAPC, estimated annual percentage change; UIs, uncertainty intervals; CI, conﬁdence interval.**
